# An examination from 1990 to 2019: investigating the burden of knee dislocation on a global scale

**DOI:** 10.3389/fpubh.2024.1396167

**Published:** 2024-05-09

**Authors:** Cheng Chen, Bing Li, Haichao Zhou, Tianbao Ye, Yunfeng Yang

**Affiliations:** ^1^Department of Orthopedics, Tongji Hospital, School of Medicine, Tongji University, Shanghai, China; ^2^Department of Orthopaedics, Ruijin Hospital, Shanghai Jiao Tong University School of Medicine, Shanghai, China; ^3^Department of Cardiology, Shanghai Sixth People's Hospital Affiliated to Shanghai Jiao Tong University School of Medicine, Shanghai, China

**Keywords:** knee dislocation, epidemiology, incidence, years lived with disability, global burden of disease

## Abstract

**Background:**

The literature on the disease burden of knee dislocation is lacking. The aim of the study is to systematically assess the global burden, trends, causes, and influencing factors of knee dislocation.

**Methods:**

The incidence and years lived with disability (YLDs) of knee dislocation were assessed globally, as well as at the regional and national levels from 1990 to 2019. Subsequent analyses focused on the age and gender distribution related to knee dislocation. An investigation into the main causes of knee dislocation followed. Finally, the Pearson correlation between age-standardized rates and social-demographic index (SDI) was calculated.

**Results:**

Although the age-standardized incidence and YLDs rate of knee dislocation decreased over the past 30 years, the incidence and YLDs number increased. The disease burden remained higher in males compared to females. Males and females showed different patterns of incidence rates in each age group, but their YLDs rates were similar. Over the past 30 years, the disease burden of knee dislocation increased in the older population while declining in the younger population. Falls had consistently emerged as the most important cause for both incidence and YLD rates. Additionally, a positive correlation between SDI and the disease burden of knee dislocation was found.

**Conclusion:**

The disease burden of knee dislocation remains heavy. It is essential to recognize the evolving epidemiology of knee dislocation. Utilizing data-driven assessments can assist in formulating public health policies and strategies to improve overall well-being.

## Introduction

Knee dislocation is a rare but serious injury with disabling potential (1). Knee dislocation accounts for approximately 3–18 per 10,000 trauma cases (2–4). Early complications were found to be as high as 32% by Scarcella et al. (5). Vascular and nerve damage, once they occur, are emergencies and must be diagnosed and treated early to prevent loss of limb function. A recent meta-analysis study showed that 10.7% of knee dislocations resulted in vascular injury, 19.6% in nerve injury, and 2.2% in amputation (6). Additionally, heterotopic ossification is a common issue after knee dislocation, occurring in 26–43% of cases (7–10). Heterotopic ossification may restrict knee joint movement, reduce its function, and in severe cases, require additional surgery. Arthrofibrosis, as another common but easily overlooked complication, occurred in 12.1% of knee dislocation patients (11). Knee dislocation often requires surgical treatment (12), and the optimal management is still controversial (13, 14). A study with a minimum follow-up period of 5 years revealed that most knee dislocation patients had mild functional impairment, with nearly a quarter developing arthritis (15). Long-term follow-up results showed that 27 out of 65 cases (42%) of knee dislocation developed osteoarthritis 10 years after surgery (16). The treatment cost of knee dislocation was high, with an average direct medical cost of $1888, higher than dislocations of the shoulder, elbow, wrist, hip, and ankle joints (17).

The literature on the incidence of knee dislocation is seriously lacking. Previous studies on knee dislocation have been limited to simple descriptive statistics or small sample sizes. It is important to note that knee dislocation can spontaneously reduce or reduce easily with minimal assistance. Clinicians often find it difficult to determine if a knee is dislocated based solely on physical examination. Therefore, the incidence of knee dislocation is often underestimated. The annual incidence rate of knee dislocation in Taiwan between 2000 and 2005 was estimated to be 1.4 per 100,000 people (95% CI 0.7–2.1) (17). A large-scale survey in mainland China estimated the incidence rate of knee dislocation in 2014 to be 3 per 100,000 people (95% CI 2–5) (18). Although few studies involved a larger sample size, they did not provide specific incidence rates (3, 4, 19, 20). In recent years, the epidemiological characteristics of knee dislocation may have shifted due to technological and socioeconomic advancements, as well as alterations in lifestyle and production practices. Previously reported knee dislocations were mostly due to high-energy injuries, but in recent years, knee dislocation caused by low-energy incidents is receiving more attention. These low-speed accidents are more common in the obese population (21–23). Therefore, there is an urgent need to update the global epidemiological trends of knee dislocation, which is crucial for developing targeted health policies and resource allocation.

The Global Burden of Disease (GBD) 2019 systematically examines 369 diseases and injuries, as well as 87 risk factors, spanning 204 countries and territories over the period from 1990 to 2019 (24). The GBD 2019 adheres to the guidelines for Accurate and Transparent Health Estimation Reporting for Population Health Research, ensuring methodological rigor and transparency in its analyses. The GBD study provides a global health data resource used for disease burden assessment, aiming to help governments, policymakers, and researchers better understand the global burden of disease (25–27). The GBD 2019 provides a platform for cross-national and interdisciplinary collaboration, facilitating knowledge sharing and cooperation in the global health field, and driving improvements in global health and the reduction of health inequalities. This enables them to develop more effective health policies and interventions. This study is based on GBD 2019 data, aiming to systematically assess the global burden, trends, causes, and influencing factors of knee dislocation to help formulate more rational policies and demonstrate the necessity of such policies.

## Methods

The modeling process of the GBD 2019 involves a standardized and rigorous approach to quantifying the burden of diseases and injuries. The final outputs of the GBD study included detailed burden estimates for each specific disease and injury category, stratified by age, gender, year, and location, providing valuable insights for public health policy and resource allocation decisions. Notably, GBD 2019 divided 204 countries and territories into 21 GBD regions^1^.

The social-demographic index (SDI) is used to assess the development status related to health outcomes. SDI is a composite index calculated from three indicators: total fertility rate of the population under 25 years old, average educational attainment of the population aged 15 and above, and *per capita* lagged income distribution. The range of SDI values is 0 to 1. A value of 1 represents the highest level of development related to health. The SDI offers a multidimensional perspective on social-demographic development that goes beyond traditional indicators. This allows researchers and policymakers to assess the overall well-being and quality of life of populations, identify disparities and inequalities, and track progress over time. The SDI also enables comparisons across regions and countries, facilitating the identification of best practices and the formulation of targeted interventions to improve health outcomes and social welfare. SDI data, as well as population data, is sourced from the GBD website^2^.

From GBD 2019, estimates of knee dislocation data from 1990 to 2019 were collected. The years lived with disability (YLDs) is a measure of the quality of life, referring to the duration of healthy life compromised by knee dislocation-induced disability. YLDs quantifies the number of years that individuals live with a disability, taking into account the severity and duration of the disability. This measure helps to capture the non-fatal consequences of diseases and injuries, which are often overlooked when focusing solely on mortality rates. ‘Crude’ refers to data that has not undergone age standardization. The methods for age standardization are provided by GBD. The uncertainty interval (UI) denotes the actual probability distribution surrounding the accurate parameter value, derived from 1,000 sampling iterations conducted during the estimation phase. The 95% UI was determined based on the 2.5th and 97.5th percentile of the sampled outcomes.

### Statistical analysis

The burden of knee dislocation was evaluated on a global scale, as well as at the GBD regional and national levels. Incidence and YLDs were directly sourced from the GBD results tool.^3^ The estimated annual percentage change (EAPC) with 95% confidence intervals (CI) for the period from 1990 to 2019 was computed (28). A positive EAPC signifies an upward trend, while a negative EAPC indicates a downward trend. Subsequent analyses delved into the age and gender profiles associated with knee dislocation. Following this, an exploration of the primary etiological factors contributing to knee dislocation was undertaken. Lastly, the Pearson correlation between age-standardized rates and SDI was determined. Statistical significance was set at *p* < 0.05. All statistical analyses and data visualizations were performed using R software (version 4.2.2).

## Results

### The burden of knee dislocation at the global level

**Figure 1:**
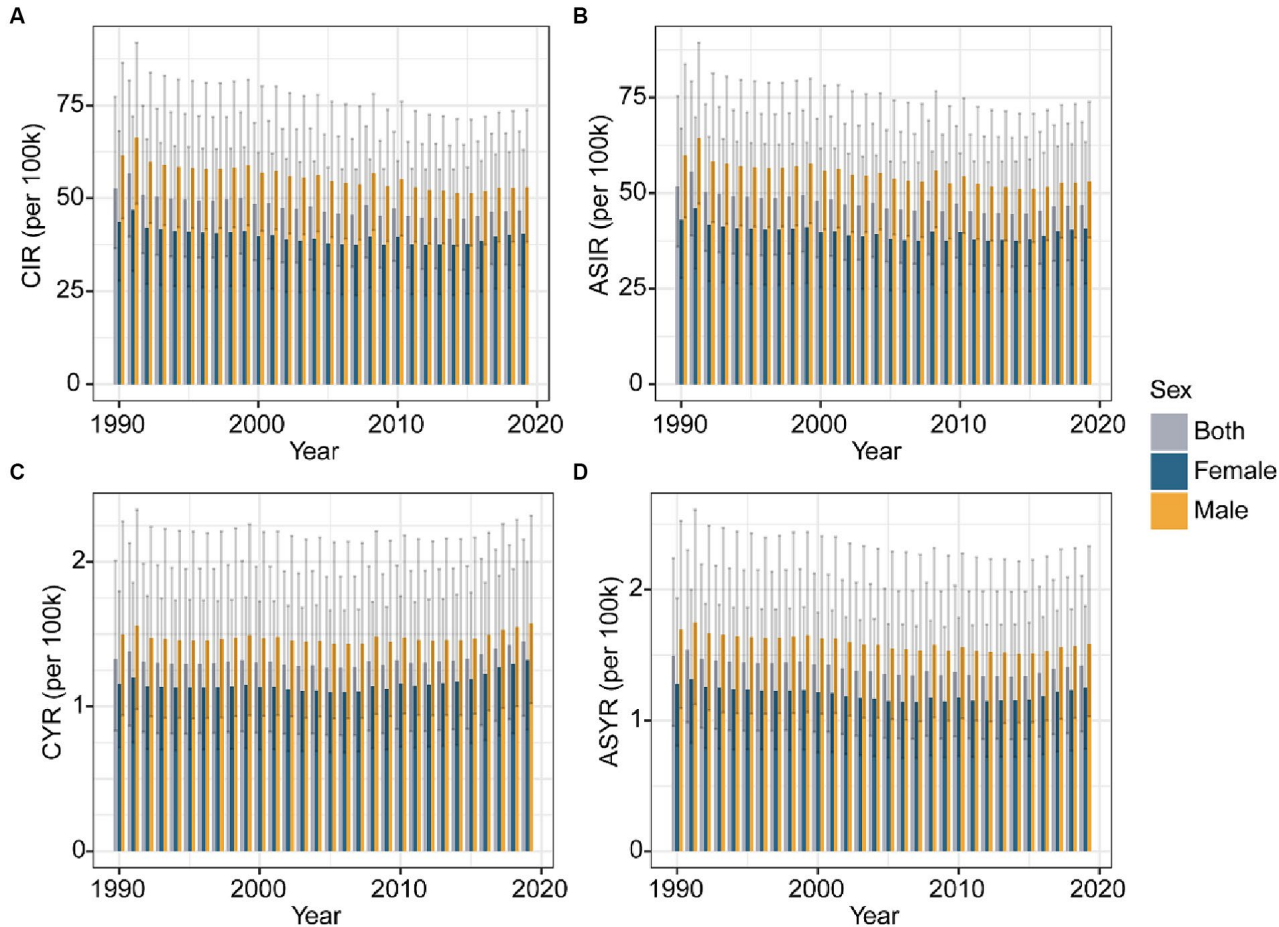
Global trends of CIR **(A)** and ASIR **(B)** of knee dislocation by gender from 1990 to 2019. Global trends of CYR **(C)** and ASYR **(D)** of knee dislocation by gender from 1990 to 2019. CIR, crude incidence rate; ASIR, age-standardized incidence rate; YLDs, years lived with disability; CYR, crude YLDs rate; ASYR, age-standardized YLDs rate.

From 1990 to 2019, the total global incidence number of knee dislocation increased from 2.82 million cases (95% UI 1.96 to 4.13) to 3.62 million cases (95% UI 2.48 to 5.26). In contrast, both the age-standardized and crude incidence rates slightly decreased over these 30 years. The crude incidence rate decreased from 52.65 per 100,000 people (95% UI 36.55 to 77.28) in 1990 to 46.75 per 100,000 people (95% UI 32.03 to 68.00) in 2019, with an EAPC of −0.53 (95% CI -0.66 to −0.41) % per year. The age-standardized incidence rate decreased from 51.74 per 100,000 people (95% UI 35.98 to 75.32) in 1990 to 46.99 per 100,000 people (95% UI 32.33 to 68.57) in 2019, with an EAPC of −0.45 (95% CI -0.58 to −0.33) % per year (Supplementary Table S1). Over these 30 years, both the crude and age-standardized incidence rates were higher in males than in females (Figures 1A,B).

From 1990 to 2019, the total global YLDs number of knee dislocation increased from 71.01 thousand cases (95% UI 44.62 to 107.51) to 112.10 thousand cases (95% UI 72.37 to 166.56). Similarly, the crude YLDs rate slightly increased over these 30 years. The crude YLDs rate increased from 1.33 per 100,000 people (95% UI 0.83 to 2.01) in 1990 to 1.45 per 100,000 people (95% UI 0.94 to 2.15) in 2019, with an EAPC of 0.15 (95% CI 0.03 to 0.28) % per year. In contrast, the age-standardized YLDs rate slightly decreased over the 30 years. The age-standardized YLDs rate decreased from 1.49 per 100,000 people (95% UI 0.96 to 2.24) in 1990 to 1.42 per 100,000 people (95% UI 0.91 to 2.11) in 2019, with an EAPC of −0.33 (95% CI -0.43 to −0.23) % per year (Supplementary Table S2). Throughout these three decades, both the crude and age-standardized YLDs rates were higher in males than in females (Figures 1C,D).

### The burden of knee dislocation at the GBD regional level

As shown in Supplementary Table S1, in 2019, the age-standardized incidence rate of knee dislocation was highest in Australasia [132.82 per 100,000 population (95% UI 85 to 208.48)], Central Europe [108.5 (95% UI 71.21 to 163.34)], and Eastern Europe [96.01 (95% UI 63.82 to 141.76)]. In contrast, the lowest age-standardized incidence rates were observed in Central Sub-Saharan Africa [22.61 (95% UI 15.9 to 31.62)], Oceania [29.83 (95% UI 19.36 to 46.41)], and Western Sub-Saharan Africa [32.07 (95% UI 22.43 to 45.15)]. Furthermore, apart from 7 GBD regions (Caribbean, Central Latin America, Oceania, Australasia, Western Sub-Saharan Africa, Andean Latin America, and Central Sub-Saharan Africa), most regions showed a decreasing trend. Among them, the highest increasing trend was observed in Caribbean [EAPC 0.86 (95% CI -0.75 to 2.5) % per year], while the highest decreasing trend was seen in High-income North America [EAPC -0.83 (95% CI -1.2 to −0.47) % per year].

Supplementary Table S2 summarizes the age-standardized YLDs rates of knee dislocation in different regions. Australasia [2.78 (95% UI 1.64 to 4.51)], High-income North America [2.53(95% UI 1.66 to 3.78)], and Central Europe [2.42 (95% UI 1.44 to 3.85)] had the highest age-standardized YLDs rates per 100,000 population. In contrast, the lowest age-standardized YLDs rates were observed in Central Sub-Saharan Africa [0.62 (95% UI 0.39 to 0.92)], Oceania [0.83 (95% UI 0.51 to 1.26)], and Andean Latin America [0.91 (95% UI 0.56 to 1.39)]. Additionally, the largest decreasing trend was observed in Eastern Europe [EAPC −0.66 (95% CI −0.83 to −0.5) % per year], while the largest increasing trend was seen in Caribbean [EAPC 1.48 (95% CI 0.36 to 2.62) % per year].

### The burden of knee dislocation at the national level

Supplementary Table S3 showed that in 2019, the countries and territories with the highest age-standardized incidence rates per 100,000 population were New Zealand [151.31 (95% UI 98.99 to 237.03)], Australia [129.4 (95% UI 82.6 to 202.79)], and Slovenia [128.27 (95% UI 82.59 to 193.28)]. In contrast, the countries with the lowest age-standardized incidence rates were Democratic People’s Republic of Korea [16.05 (95% UI 11.05 to 22.96)], Taiwan (Province of China) [18.60 (95% UI 12.48 to 27.96)], and Kiribati [20.47 (95% UI 13.78 to 31.22)]. Additionally, Taiwan (Province of China) had the highest decreasing trend [EAPC −1.93 (95% CI -2.36 to −1.51) % per year], while Belgium had the highest increasing trend [EAPC 1.16 (95% CI 0.66 to 1.66) % per year].

Supplementary Table S4 showed that in 2019, the countries and territories with the highest age-standardized YLDs rates per 100,000 population were Greenland [3.33 (95% UI 2.19 to 4.89)], New Zealand [3.15 (95% UI 1.86 to 5.07)], and Slovenia [2.9 (95% UI 1.7 to 4.61)]. In contrast, the countries with the lowest age-standardized YLDs rates were Democratic People’s Republic of Korea [0.45 (95% UI 0.28 to 0.67)], Kiribati [0.53 (95% UI 0.32 to 0.81)], and Taiwan (Province of China) [0.54 (95% UI 0.34 to 0.82)]. Additionally, Armenia had the highest decreasing trend [EAPC −1.94 (95% CI −2.15 to −1.72) % per year], while Haiti had the highest increasing trend [EAPC 4.13 (95% CI 1.9 to 6.41) % per year].

**Figure 2:**
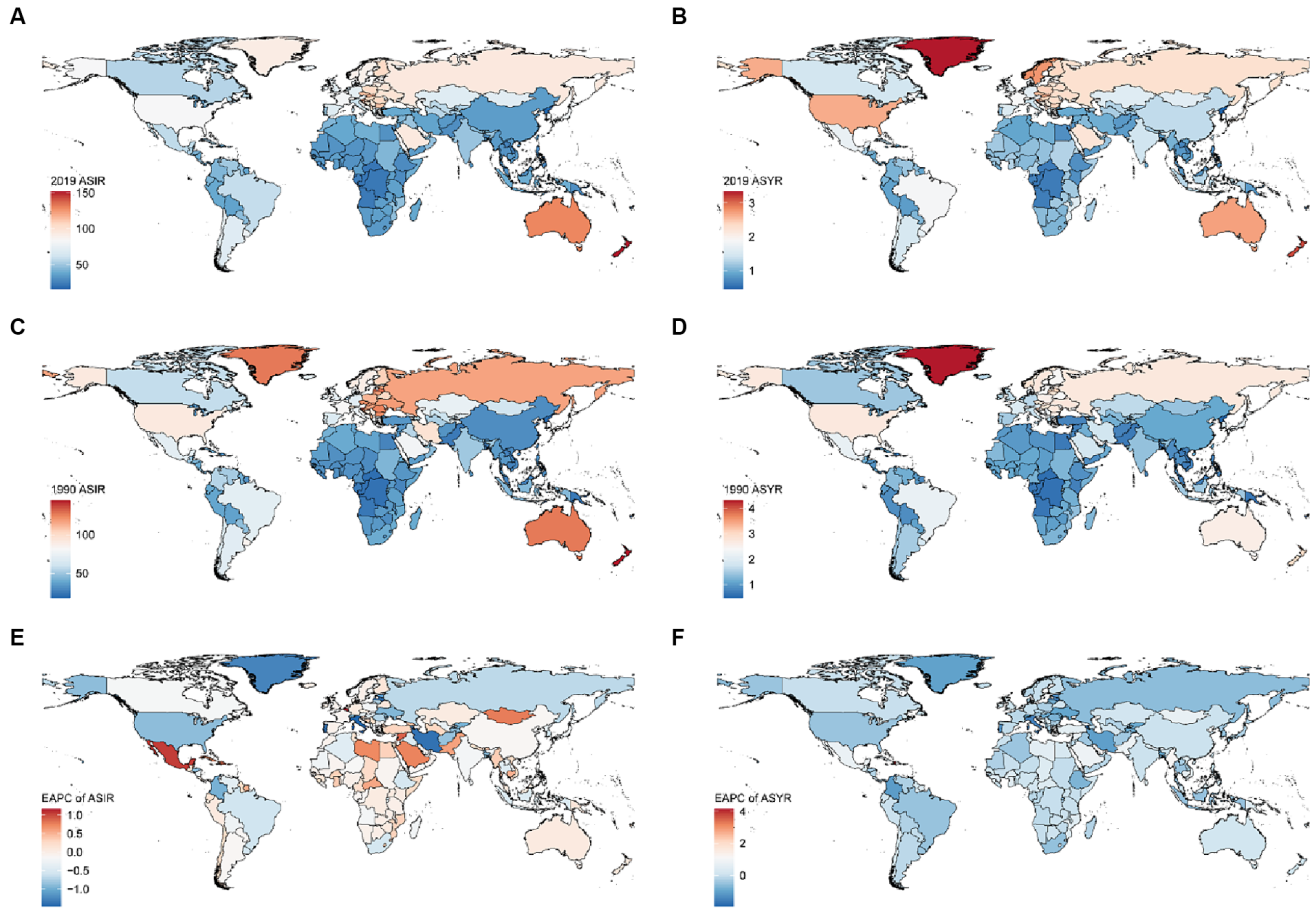
ASIR **(A)** and ASYR **(B)** maps of knee dislocation by country in 2019. ASIR **(C)** and ASYR **(D)** maps of knee dislocation by country in 1990. EPAC maps of ASIR **(E)** and ASYR **(F)** of knee dislocation per 100 k population by country from 1990 to 2019. ASIR, age-standardized incidence rate; YLDs, years lived with disability; ASYR, age-standardized YLDs rate; EAPC, estimated annual percentage change.

The further visualization of the results is presented in the form of a map, where colors correspond to the values of the parameter under study (Figure 2).

### Age and gender patterns of knee dislocation

**Figure 3:**
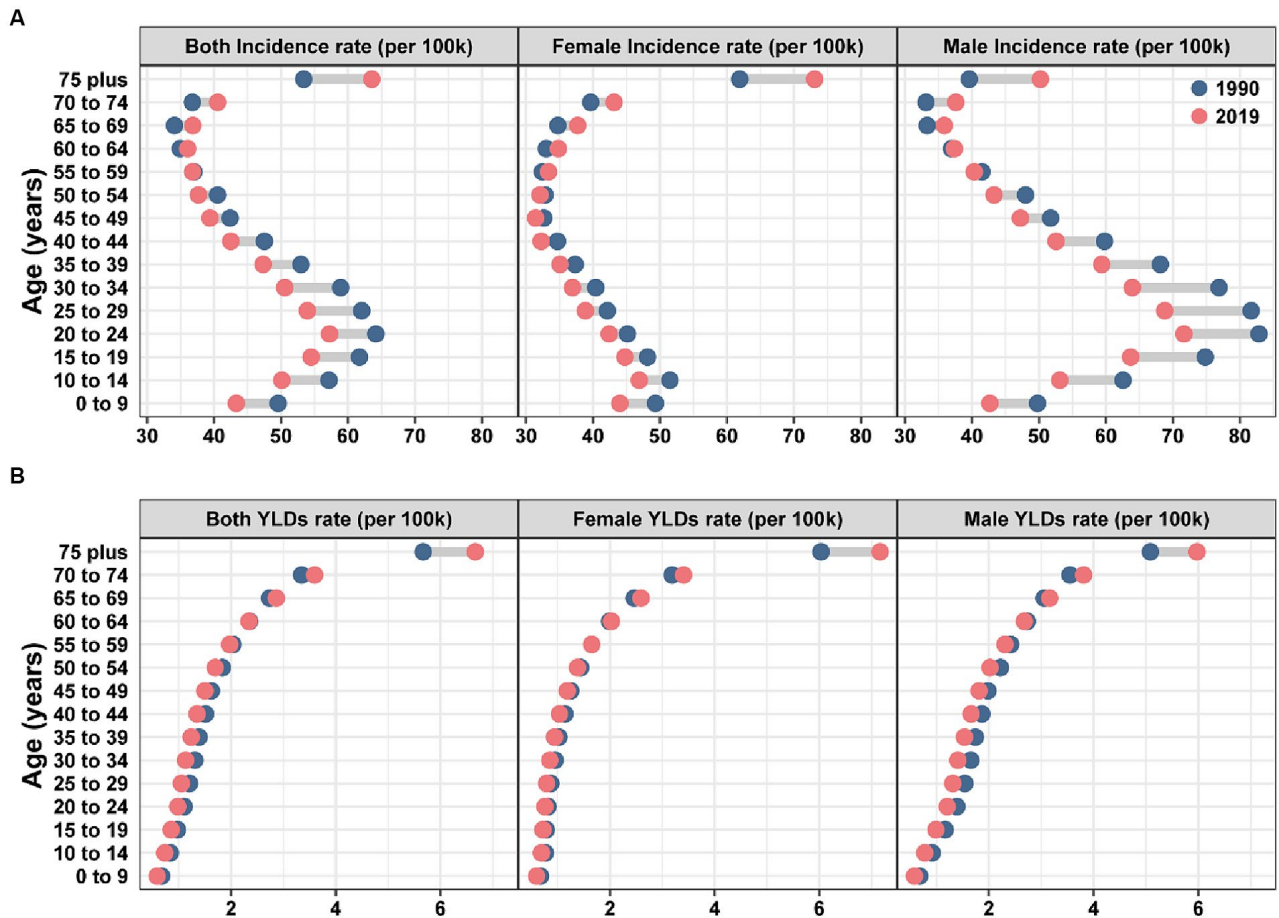
Global incidence **(A)** and YLDs **(B)** rates of knee dislocation by age and gender in 1990 and 2019. YLDs, years lived with disability.

Figure 3 showed the burden of knee dislocation in different genders and age groups from 1990 to 2019. The incidence rate in males peaked in the 20–24 age group, then decreased, and rose again around the age of 70. The incidence rate in females started to decline at the age of 10–14, began to rise around 60 years old, and eventually peaked in the age group of 75 plus, significantly higher than other age groups (Figure 3A). However, the trends of YLDs rate were different from the incidence rate (Figure 3B). With increasing age, the YLDs rate increased, and this trend was the same for different genders. A comparison from 1990 to 2019 showed that both the incidence rate and YLDs rate of knee dislocation in young people decreased overall, while the total burden of knee dislocation in older people increased.

### Leading causes of knee dislocation

**Figure 4:**
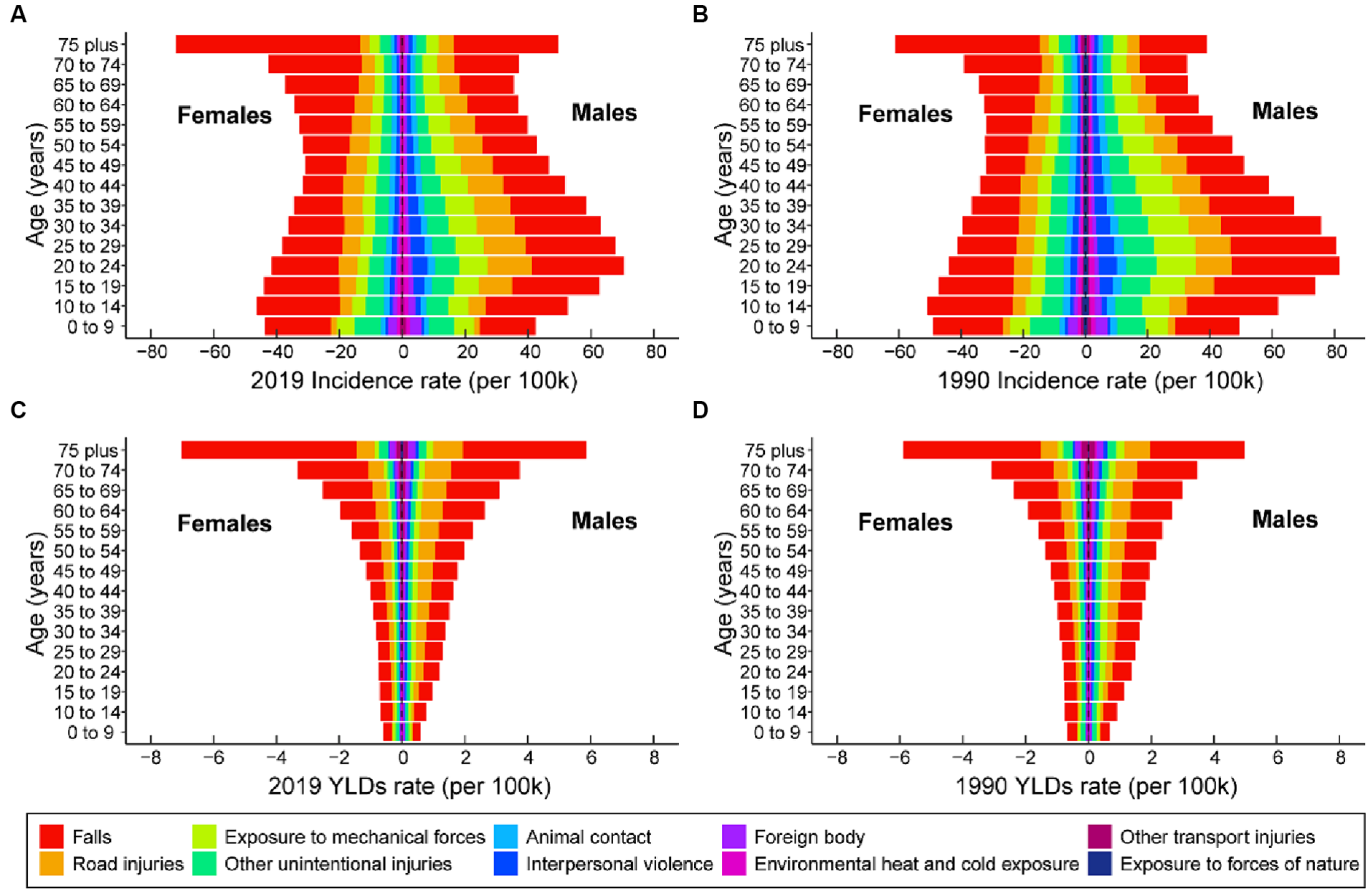
The 10 leading causes of knee dislocation incidence rate, categorized by age and gender, for the years 2019 **(A)** and 1990 **(B)**. The 10 leading causes of knee dislocation YLDs rates, categorized by age and gender, for the years 2019 **(C)** and 1990 **(D)**. YLDs, years lived with disability.

Figure 4 showed the incidence rate and YLDs rate of knee dislocation for the 10 main causes classified by gender and age. For the overall population, the top 4 main causes of incidence rate and YLDs rate in 1990 and 2019 were the same, namely falls, road injuries, exposure to mechanical forces, and other unintentional injuries. Among these, falls had consistently been the most important cause for both incidence and YLDs rate. The age-standardized incidence rate of knee dislocation caused by falls for overall population was 22.97 (95% UI 11.83 to 43.08) in 1990 and 22.16 (95% UI 11.54 to 40.93) in 2019. The age-standardized YLDs rate of knee dislocation caused by falls for the overall population was 0.75 (95% UI 0.44 to 1.20) in 1990 and 0.75 (95% UI 0.45 to 1.18) in 2019. To note, falls were the primary cause of knee dislocation in older women and a major contributor to the high YLDs rate in the older population.

### Correlation between age-standardized rate and SDI

**Figure 5:**
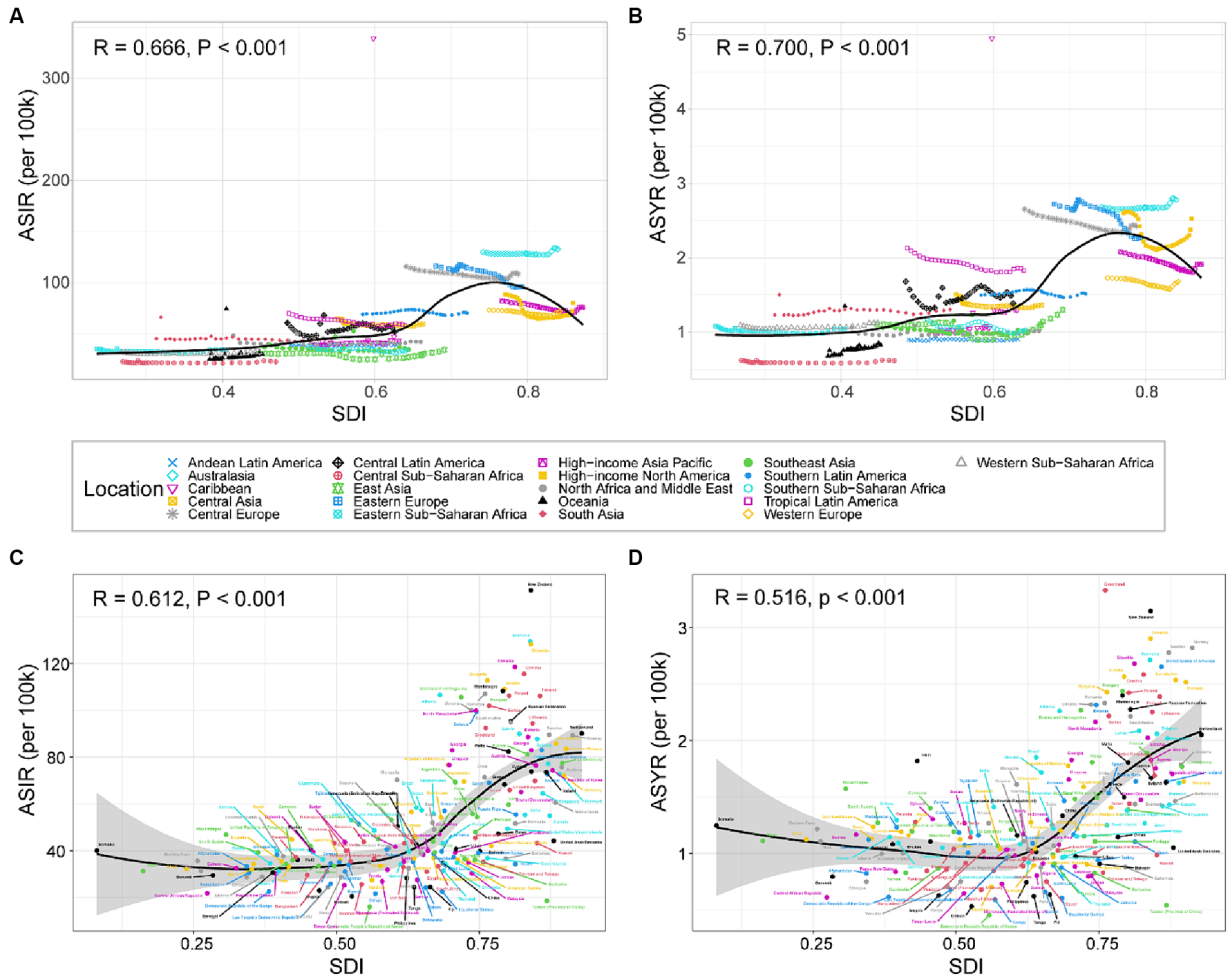
ASIR **(A)** and ASYR **(B)** of knee dislocation across 21 GBD regions by SDI from 1990 to 2019. ASIR **(C)** and ASYR **(D)** of knee dislocation for 204 countries and territories by SDI in 2019. Trendlines are depicted by black lines, with R- and *p*-values calculated using Pearson’s correlation analysis. ASIR, age-standardized incidence rate; YLDs, years lived with disability; ASYR, age-standardized YLDs rate; SDI, sociodemographic index.

Overall, there was a positive correlation between the age-standardized incidence and YLDs rate of knee dislocation and SDI (Figure 5). From 1990 to 2019, with the increase in SDI, the age-standardized incidence (Figure 5A) and YLDs (Figure 5B) rate of knee dislocation in the 21 GBD regions showed an S-shaped trend. The high age-standardized incidence rate of Australasia, Eastern Europe, and Central Europe caused an inflection point in the fitted curve, followed by a decrease in the curve as SDI increased. On the other hand, Australasia, Eastern Europe, Central Europe, and High-income North America had high age-standardized YLDs rate, resulting in the inflection point of the fitted curve. It is worth noting that there was a clear outlier, which was the Caribbean in 2010, where the significantly high incidence and YLDs rate might be attributed to the 2010 Haiti earthquake. At the national level in 2019, when SDI was around 0.6, the curves of age-standardized incidence and YLDs rate became steeper, showing a more significant increase (Figures 5C,D). It is noteworthy that some countries and regions, such as New Zealand and Slovenia, had actual age-standardized incidence and YLDs rates significantly higher than expected.

## Discussion

This study comprehensively explored the changes in the global burden of knee dislocation over the past 30 years and its influencing factors. Through this study, researchers and decision-makers can gain an in-depth understanding of the epidemiology of knee dislocation, as well as the trend changes. This study provides scientific evidence for formulating health policies, optimizing resource allocation, and guiding public health interventions.

Although the age-standardized incidence and YLDs rate of knee dislocation decreased over the past 30 years, the total global incidence and YLDs number increased. This indicates that knee dislocation remained a significant burden worldwide. It is worth noting that the disease burden in males continued to be higher than in females. The study also analyzed the disease burden of knee dislocation divided by age and gender. Males and females showed different patterns of incidence rates in each age group, but their patterns of YLDs rates by age were similar. We observed that over the past 30 years, the disease burden of knee dislocation increased in the older population while decreasing in the younger population. This suggests that with the increasing aging population, the epidemiological pattern of knee dislocation was changing. The top 4 main causes of incidence and YLDs rate in 1990 and 2019 were the same, namely falls, road injuries, exposure to mechanical forces, and other unintentional injuries. Among these, falls had consistently been the most important cause for both incidence and YLDs rate. Additionally, we found a positive correlation between SDI and the disease burden of knee dislocation.

The results showed that the burden of knee dislocation in males continued to be higher than in females, consistent with previous literature (3, 29–31). Stewart et al.’s (20) findings are similar to ours, but it is worth noting that in knee dislocation caused by ultra-low energy injuries, females accounted for a higher percentage (64%) than males (36%). Previous studies showed that young adult males were a high-risk group for knee dislocation, and we had similar findings. We found that the incidence rate in males peaked in the 20–24 age group, then decreased, and rose again around the age of 70. However, females exhibited a different epidemiological pattern. The incidence rate in females started to rise around the age of 60, and ultimately peaked in the age group of 75 and above, significantly higher than in other age groups. The higher incidence rate in young males may be related to their greater involvement in high-risk occupations and activities. Older individuals, especially females, are another major risk group. Frailty is one of the most important health issues in the older population. Progressive deterioration of age-related physiological systems leads to extreme vulnerability to stressors and increases the risk of a range of adverse outcomes (32).

Our study indicated that the most common cause of knee dislocation was falls, which corresponded with previous reports (30). With the aging population, there come enormous challenges (33, 34). In addition, the health life expectancy may continue to increase (35). The burden of falls is huge, and the older population is considered to be the most concentrated. This renders falls among the older population a critical public health concern (36, 37). To reduce the incidence of falls and related injuries among the older population, collaboration between communities, care facilities, and hospitals is needed to implement comprehensive system measures, such as regular fall risk assessments and personalized intervention plans (38–40).

Another focus group for falls is the population of obese/overweight individuals. Research shows that from 2000 to 2012, the proportion of obese and morbidly obese patients among knee dislocation patients in the United States increased (41). Obesity has become a serious public health issue, with a noticeable increase in the prevalence of overweight and obesity (42, 43). The study revealed that ultra-low velocity knee dislocation occurred more frequently in patients with higher BMI, with falls being a common mechanism of injury (23). Based on a study of the American College of Surgeons National Trauma Data Bank from 2010 to 2012, it was found that 456 out of 1,324 (34.4%) knee dislocations were due to low-energy or ultra-low-energy injuries, with obesity accounting for 18.4% of knee dislocation in low-energy and ultra-low-energy injuries (20). A study conducted in a level 1 trauma center in Finland from 2000 to 2007 found that out of 24 cases of knee dislocations, 11 cases (46%) had a body mass index greater than 25, and were due to low-energy trauma (9 cases were due to falls, 2 cases were due to non-contact sports) (21). Similarly, Vaidya et al. introduced 19 cases of low-velocity knee dislocation in obese and morbidly obese patients (22).

Given that the disease burden of knee dislocation has not lessened, its rehabilitation is also a point that needs attention. Loss of motion is not uncommon after surgery for multiple knee ligament injuries (44). Unfortunately, the rehabilitation of knee dislocation remains full of unknowns (45). The early rehabilitation after multiligamentous reconstruction of the knee joint may have a slightly superior but not significantly better effect, compared with late knee rehabilitation (46). It is necessary to conduct more large-scale high-level research to explore the optimal rehabilitation program, improving patient prognosis and reducing disability. There is a pressing need to increase investment in rehabilitation in healthcare and improve the quality of rehabilitation services. Implementing a healthcare rehabilitation plan and targeted intervention programs can lead to benefits for individuals with knee dislocation.

Furthermore, our study systematically investigated the disease burden of knee dislocation in different regions and countries worldwide. At the national and regional levels, the disease burden of knee dislocation was not evenly distributed. The level of social-demographic development may be a crucial factor contributing to this unevenness. The SDI provides a comprehensive tool for assessing the level of social-demographic development, aiding researchers in quantifying and understanding the disparities in social-demographic development among different regions or countries (47–49). We found a positive correlation between the disease burden of knee dislocation and SDI. Countries with low SDI tend to have inadequate healthcare services and low healthcare coverage rates, resulting in barriers for patients to access medical care, potential misdiagnoses, and underestimation of the disease burden related to knee dislocation. In contrast, countries with high SDI offer extensive access to high-quality healthcare services, which likely provides a more accurate representation of the actual disease burden. Additionally, these countries, characterized by an aging population, face a more substantial healthcare burden.

### Limitations of this study

This study has some limitations. Firstly, GBD 2019 relies on diverse data sources. The collection and reporting standards of these data may vary, affecting the homogeneity of the data. Besides, the GBD 2019 uses models and estimation methods to assess disease burden. Inferring and estimating based on existing data and models may lead to bias and uncertainty in the results. Furthermore, the study does not involve subnational and subtype levels of knee dislocation. Despite the growing attention on low-energy knee dislocation (more common in obese individuals), GBD 2019 does not cover corresponding disaggregated data. In addition, GBD 2019 has limited research on the economic and psychological impacts when considering disease burden.

## Conclusion

The disease burden of knee dislocation remains heavy. It is essential to recognize the evolving epidemiology of knee dislocation. Utilizing data-driven assessments can assist in formulating public health policies and strategies to improve overall well-being.

## Data availability statement

The original contributions presented in the study are included in the article/Supplementary material, further inquiries can be directed to the corresponding authors.

## Ethics statement

Ethical approval was not required for the study involving humans in accordance with the local legislation and institutional requirements. Written informed consent to participate in this study was not required from the participants or the participants’ legal guardians/next of kin in accordance with the national legislation and the institutional requirements.

## Author contributions

CC: Conceptualization, Writing – original draft. BL: Writing – original draft, Investigation. HZ: Writing – review & editing, Validation, Supervision. TY: Writing – review & editing, Conceptualization, Formal analysis, Visualization, Data curation, Methodology. YY: Writing – review & editing, Funding acquisition, Project administration, Validation.

## Footnotes

^1^https://ghdx.healthdata.org/countries

^2^https://ghdx.healthdata.org/gbd-2019

^3^https://vizhub.healthdata.org/gbd-results/
