# Supplementary material for: An examination from 1990 to 2019: investigating the burden of knee dislocation on a global scale
Source: Front Public Health. 2024 May 9;12:1396167. doi: 10.3389/fpubh.2024.1396167 (PMC11111932; doi:10.3389/fpubh.2024.1396167)
Supplement: Supplementary file 1 [file Data_Sheet_1.docx]

**An Examination from 1990 to 2019:**

**Investigating the Burden of Knee Dislocation on a Global Scale**

**Supplementary Material**

**Contents**

**Table S1………………………………………………………………………………………. 2**

**Table S2……………………………………………………………………………….……… 7**

**Table S3………………………………………………………………………………………. 12**

**Table S4………………………………………………………………………………………. 53**

Table S1. Incidence of knee dislocation in 1990 and 2019 for both sexes in globe and 21 GBD regions, with EAPC from 1990 and 2019.

| **Location** | **Number** | | | **CR, per 100k** | | | **ASR, per 100k** | | |  |
| --- | --- | --- | --- | --- | --- | --- | --- | --- | --- | --- |
|  | **Number in 1990 (95% UI)** | **Number in 2019 (95% UI)** | **Number change rate (95% UI)** | **CR in 1990 (95% UI)** | **CR in 2019 (95% UI)** | **EAPC of CR, % per year (95% CI)** | **ASR in 1990 (95% UI)** | **ASR in 2019 (95% UI)** | **EAPC of ASR, % per year (95% CI)** |  |
| Global | 2816621 (1955406 to 4134109) | 3617356 (2478381 to 5261844) | 0.28 (0.23 to 0.34) | 52.65 (36.55 to 77.28) | 46.75 (32.03 to 68) | -0.53 (-0.66 to -0.41) | 51.74 (35.98 to 75.32) | 46.99 (32.33 to 68.57) | -0.45 (-0.58 to -0.33) |  |
| Andean Latin America | | 16092 (11733 to 22128) | 25516 (17915 to 36255) | 0.59 (0.44 to 0.72) | 42.15 (30.73 to 57.96) | 40.12 (28.17 to 57.01) | -0.2 (-0.26 to -0.14) | 38.56 (28.1 to 53.16) | 39.41 (27.66 to 55.9) | 0.04 (-0.02 to 0.1) |
| Australasia | 25136 (16736 to 38197) | 33700 (22289 to 51436) | 0.34 (0.27 to 0.42) | 123.96 (82.53 to 188.37) | 115.95 (76.69 to 176.98) | -0.25 (-0.32 to -0.18) | 129.93 (85.06 to 199.36) | 132.82 (85 to 208.48) | 0.05 (0 to 0.11) |  |
| Caribbean | 14540 (10715 to 20014) | 20058 (14438 to 28097) | 0.38 (0.29 to 0.46) | 41.22 (30.38 to 56.74) | 42.52 (30.61 to 59.57) | 0.65 (-0.96 to 2.28) | 39.28 (28.86 to 54.02) | 43.15 (31.08 to 60.32) | 0.86 (-0.75 to 2.5) |  |
| Central Asia | 48310 (32688 to 70605) | 56892 (38135 to 83200) | 0.18 (0.14 to 0.22) | 69.75 (47.19 to 101.93) | 60.83 (40.77 to 88.95) | -0.48 (-0.53 to -0.43) | 64.92 (43.99 to 94.16) | 59.45 (39.88 to 87.17) | -0.31 (-0.39 to -0.23) |  |
| Central Europe | 138438 (93388 to 205071) | 106718 (70493 to 158509) | -0.23 (-0.28 to -0.19) | 112.58 (75.95 to 166.77) | 93.43 (61.72 to 138.77) | -0.7 (-0.76 to -0.65) | 115.82 (77.98 to 171.66) | 108.5 (71.21 to 163.34) | -0.26 (-0.34 to -0.17) |  |
| Central Latin America | 108717 (74777 to 161993) | 128361 (87539 to 192073) | 0.18 (0.13 to 0.23) | 66.24 (45.56 to 98.7) | 51.34 (35.01 to 76.82) | -0.05 (-0.39 to 0.29) | 60.6 (42.05 to 89.82) | 51.77 (35.35 to 77.64) | 0.25 (-0.08 to 0.59) |  |
| Central Sub-Saharan Africa | 13767 (9658 to 18938) | 32013 (22309 to 45105) | 1.33 (1.22 to 1.44) | 24.8 (17.4 to 34.11) | 24.34 (16.96 to 34.29) | -0.04 (-0.16 to 0.09) | 22.7 (15.96 to 31.63) | 22.61 (15.9 to 31.62) | 0.01 (-0.11 to 0.13) |  |
| East Asia | 379444 (260796 to 595707) | 518529 (355386 to 789131) | 0.37 (0.21 to 0.56) | 30.97 (21.29 to 48.62) | 35.22 (24.14 to 53.6) | -0.07 (-0.45 to 0.31) | 30.63 (21.07 to 47.81) | 34.2 (23.53 to 52.08) | -0.15 (-0.51 to 0.22) |  |
| Eastern Europe | 255790 (172559 to 373957) | 183927 (123240 to 271288) | -0.28 (-0.31 to -0.25) | 112.93 (76.18 to 165.1) | 87.6 (58.69 to 129.2) | -0.86 (-1.1 to -0.63) | 116.45 (78.56 to 170.33) | 96.01 (63.82 to 141.76) | -0.71 (-0.86 to -0.55) |  |
| Eastern Sub-Saharan Africa | 70461 (49252 to 98167) | 149195 (103652 to 212218) | 1.12 (1.04 to 1.21) | 37.05 (25.9 to 51.62) | 36.23 (25.17 to 51.54) | -0.08 (-0.23 to 0.07) | 35.14 (25.08 to 49.93) | 34.09 (24.12 to 48.45) | -0.12 (-0.26 to 0.01) |  |
| High-income Asia Pacific | 138593 (93157 to 206558) | 122570 (84479 to 181089) | -0.12 (-0.16 to -0.07) | 79.88 (53.69 to 119.05) | 65.44 (45.11 to 96.69) | -0.82 (-0.92 to -0.72) | 82.19 (54.93 to 122.06) | 75.85 (50.2 to 114.76) | -0.4 (-0.52 to -0.29) |  |
| High-income North America | 247288 (167944 to 368504) | 298548 (208228 to 438209) | 0.21 (0.12 to 0.29) | 88.03 (59.78 to 131.18) | 81.89 (57.12 to 120.2) | -0.58 (-0.89 to -0.26) | 88.36 (59.56 to 132.97) | 80.28 (55.41 to 119.7) | -0.83 (-1.2 to -0.47) |  |
| North Africa and Middle East | 177713 (125264 to 247100) | 266911 (180699 to 397386) | 0.5 (0.26 to 0.71) | 51.51 (36.31 to 71.62) | 43.85 (29.69 to 65.28) | -0.24 (-0.44 to -0.04) | 48.28 (34.36 to 67.9) | 42 (28.45 to 62.02) | -0.1 (-0.33 to 0.14) |  |
| Oceania | 1708 (1152 to 2548) | 4109 (2661 to 6445) | 1.41 (1.22 to 1.58) | 26.4 (17.81 to 39.38) | 30.95 (20.05 to 48.55) | 0.09 (-0.69 to 0.88) | 25.17 (17.21 to 37.31) | 29.83 (19.36 to 46.41) | 0.12 (-0.68 to 0.93) |  |
| South Asia | 505438 (342290 to 750772) | 818131 (545943 to 1206142) | 0.62 (0.51 to 0.75) | 46.05 (31.18 to 68.4) | 45.32 (30.24 to 66.81) | -0.37 (-0.69 to -0.06) | 45.29 (30.99 to 66.62) | 44.94 (30.03 to 66.21) | -0.33 (-0.62 to -0.04) |  |
| Southeast Asia | 181942 (127947 to 261662) | 231387 (159648 to 332756) | 0.27 (0.2 to 0.34) | 38.98 (27.41 to 56.06) | 34.34 (23.69 to 49.39) | -0.39 (-0.8 to 0.02) | 37.04 (26.34 to 53.04) | 34.56 (23.77 to 49.65) | -0.2 (-0.62 to 0.22) |  |
| Southern Latin America | 34743 (23557 to 51957) | 44460 (29887 to 66358) | 0.28 (0.23 to 0.33) | 70.13 (47.55 to 104.87) | 66.6 (44.77 to 99.41) | -0.3 (-0.42 to -0.19) | 68.99 (46.8 to 103.07) | 70.23 (46.93 to 106.57) | -0.05 (-0.16 to 0.05) |  |
| Southern Sub-Saharan Africa | 20809 (14337 to 29200) | 27229 (19262 to 37729) | 0.31 (0.24 to 0.39) | 39.64 (27.31 to 55.63) | 34.65 (24.51 to 48.02) | -0.45 (-0.57 to -0.34) | 37.82 (26.33 to 53.39) | 33.94 (23.94 to 46.91) | -0.39 (-0.51 to -0.28) |  |
| Tropical Latin America | 112119 (75884 to 167445) | 128118 (87139 to 192590) | 0.14 (0.08 to 0.21) | 73.34 (49.64 to 109.53) | 57.3 (38.97 to 86.13) | -0.82 (-0.88 to -0.75) | 69.91 (47.91 to 104.27) | 58.02 (39.26 to 87.75) | -0.58 (-0.65 to -0.51) |  |
| Western Europe | 261061 (172296 to 398082) | 270773 (173343 to 428946) | 0.04 (-0.03 to 0.1) | 67.88 (44.8 to 103.51) | 62.06 (39.73 to 98.31) | -0.49 (-0.58 to -0.4) | 73.21 (47.47 to 114.01) | 72 (44.53 to 118.37) | -0.22 (-0.3 to -0.14) |  |
| Western Sub-Saharan Africa | 64512 (44620 to 90926) | 150211 (103725 to 211281) | 1.33 (1.25 to 1.42) | 33.5 (23.17 to 47.21) | 32.92 (22.73 to 46.3) | -0.04 (-0.14 to 0.06) | 31.83 (22.1 to 45.47) | 32.07 (22.43 to 45.15) | 0.05 (-0.04 to 0.14) |  |

CR, crude rate; ASR, age-standardized rate; EAPC, estimated annual percentage change; UI, uncertainty interval; CI, confidence interval.

Table S2. YLDs of knee dislocation in 1990 and 2019 for both sexes in globe and 21 GBD regions, with EAPC from 1990 and 2019.

| **Location** | **Number** | | | **CR, per 100k** | | | **ASR, per 100k** | | |
| --- | --- | --- | --- | --- | --- | --- | --- | --- | --- |
|  | **Number in 1990 (95% UI)** | **Number in 2019 (95% UI)** | **Number change rate (95% UI)** | **CR in 1990 (95% UI)** | **CR in 2019 (95% UI)** | **EAPC of CR, % per year (95% CI)** | **ASR in 1990 (95% UI)** | **ASR in 2019 (95% UI)** | **EAPC of ASR, % per year (95% CI)** |
| Global | 71010 (44616 to 107506) | 112101 (72367 to 166557) | 0.58 (0.5 to 0.66) | 1.33 (0.83 to 2.01) | 1.45 (0.94 to 2.15) | 0.15 (0.03 to 0.28) | 1.49 (0.96 to 2.24) | 1.42 (0.91 to 2.11) | -0.33 (-0.43 to -0.23) |
| Andean Latin America | 314 (192 to 487) | 566 (351 to 873) | 0.8 (0.69 to 0.92) | 0.82 (0.5 to 1.28) | 0.89 (0.55 to 1.37) | 0.26 (0.22 to 0.3) | 0.9 (0.56 to 1.36) | 0.91 (0.56 to 1.39) | 0 (-0.04 to 0.04) |
| Australasia | 547 (330 to 867) | 871 (535 to 1356) | 0.59 (0.45 to 0.78) | 2.7 (1.63 to 4.28) | 3 (1.84 to 4.67) | 0.37 (0.31 to 0.43) | 2.69 (1.6 to 4.31) | 2.78 (1.64 to 4.51) | 0.11 (0.06 to 0.16) |
| Caribbean | 329 (208 to 506) | 632 (400 to 920) | 0.92 (0.65 to 1.48) | 0.93 (0.59 to 1.43) | 1.34 (0.85 to 1.95) | 1.87 (0.74 to 3.01) | 1.01 (0.64 to 1.52) | 1.3 (0.81 to 1.91) | 1.48 (0.36 to 2.62) |
| Central Asia | 989 (587 to 1550) | 1244 (748 to 1953) | 0.26 (0.2 to 0.32) | 1.43 (0.85 to 2.24) | 1.33 (0.8 to 2.09) | -0.22 (-0.29 to -0.16) | 1.5 (0.92 to 2.36) | 1.36 (0.83 to 2.12) | -0.31 (-0.39 to -0.23) |
| Central Europe | 3375 (2109 to 5278) | 3131 (1956 to 4763) | -0.07 (-0.12 to -0.01) | 2.74 (1.72 to 4.29) | 2.74 (1.71 to 4.17) | -0.04 (-0.1 to 0.03) | 2.65 (1.64 to 4.17) | 2.42 (1.44 to 3.85) | -0.36 (-0.44 to -0.28) |
| Central Latin America | 2369 (1448 to 3742) | 3416 (2155 to 5207) | 0.44 (0.36 to 0.54) | 1.44 (0.88 to 2.28) | 1.37 (0.86 to 2.08) | 0.72 (0.43 to 1.01) | 1.68 (1.08 to 2.53) | 1.39 (0.87 to 2.11) | 0.18 (-0.09 to 0.44) |
| Central Sub-Saharan Africa | 275 (168 to 427) | 649 (392 to 1004) | 1.36 (1.27 to 1.45) | 0.5 (0.3 to 0.77) | 0.49 (0.3 to 0.76) | -0.03 (-0.15 to 0.08) | 0.62 (0.39 to 0.93) | 0.62 (0.39 to 0.92) | -0.03 (-0.12 to 0.06) |
| East Asia | 11628 (7330 to 17695) | 23102 (15229 to 33558) | 0.99 (0.8 to 1.18) | 0.95 (0.6 to 1.44) | 1.57 (1.03 to 2.28) | 1.2 (0.76 to 1.64) | 1.1 (0.71 to 1.64) | 1.3 (0.85 to 1.92) | 0.01 (-0.39 to 0.41) |
| Eastern Europe | 6499 (4044 to 9923) | 5426 (3394 to 8199) | -0.17 (-0.21 to -0.11) | 2.87 (1.79 to 4.38) | 2.58 (1.62 to 3.9) | -0.37 (-0.58 to -0.15) | 2.72 (1.67 to 4.22) | 2.27 (1.38 to 3.53) | -0.66 (-0.83 to -0.5) |
| Eastern Sub-Saharan Africa | 1523 (941 to 2343) | 3285 (2012 to 5110) | 1.16 (1.09 to 1.23) | 0.8 (0.49 to 1.23) | 0.8 (0.49 to 1.24) | -0.05 (-0.19 to 0.09) | 1.08 (0.69 to 1.59) | 1.04 (0.67 to 1.53) | -0.16 (-0.26 to -0.07) |
| High-income Asia Pacific | 3779 (2392 to 5772) | 4878 (3170 to 7109) | 0.29 (0.19 to 0.41) | 2.18 (1.38 to 3.33) | 2.6 (1.69 to 3.8) | 0.53 (0.45 to 0.6) | 2.08 (1.3 to 3.21) | 1.91 (1.18 to 3) | -0.41 (-0.5 to -0.32) |
| High-income North America | 8067 (5253 to 12031) | 12333 (8214 to 17731) | 0.53 (0.43 to 0.63) | 2.87 (1.87 to 4.28) | 3.38 (2.25 to 4.86) | 0.32 (0.04 to 0.6) | 2.6 (1.68 to 3.94) | 2.53 (1.66 to 3.78) | -0.46 (-0.75 to -0.17) |
| North Africa and Middle East | 3283 (1932 to 5245) | 5924 (3602 to 9365) | 0.8 (0.63 to 0.96) | 0.95 (0.56 to 1.52) | 0.97 (0.59 to 1.54) | 0.28 (0.14 to 0.41) | 1.06 (0.65 to 1.64) | 1.01 (0.63 to 1.57) | 0.04 (-0.09 to 0.16) |
| Oceania | 36 (21 to 57) | 92 (54 to 145) | 1.57 (1.43 to 1.75) | 0.55 (0.32 to 0.88) | 0.69 (0.4 to 1.09) | 0.45 (-0.11 to 1.02) | 0.68 (0.42 to 1.03) | 0.83 (0.51 to 1.26) | 0.4 (-0.08 to 0.88) |
| South Asia | 10950 (6547 to 17026) | 21239 (13186 to 32006) | 0.94 (0.82 to 1.07) | 1 (0.6 to 1.55) | 1.18 (0.73 to 1.77) | 0.34 (0.14 to 0.55) | 1.23 (0.77 to 1.86) | 1.3 (0.81 to 1.93) | 0.01 (-0.15 to 0.18) |
| Southeast Asia | 4160 (2622 to 6382) | 6568 (4201 to 9773) | 0.58 (0.48 to 0.7) | 0.89 (0.56 to 1.37) | 0.97 (0.62 to 1.45) | 0.31 (0.09 to 0.54) | 1.06 (0.68 to 1.6) | 1.01 (0.65 to 1.5) | -0.2 (-0.4 to 0.01) |
| Southern Latin America | 732 (438 to 1163) | 1039 (637 to 1615) | 0.42 (0.33 to 0.54) | 1.48 (0.88 to 2.35) | 1.56 (0.95 to 2.42) | 0.08 (-0.01 to 0.16) | 1.5 (0.91 to 2.36) | 1.51 (0.91 to 2.4) | -0.07 (-0.15 to 0.02) |
| Southern Sub-Saharan Africa | 506 (322 to 768) | 735 (473 to 1100) | 0.45 (0.37 to 0.54) | 0.96 (0.61 to 1.46) | 0.94 (0.6 to 1.4) | -0.03 (-0.17 to 0.1) | 1.18 (0.76 to 1.73) | 1.03 (0.67 to 1.53) | -0.38 (-0.52 to -0.24) |
| Tropical Latin America | 2798 (1746 to 4357) | 4271 (2746 to 6444) | 0.53 (0.41 to 0.66) | 1.83 (1.14 to 2.85) | 1.91 (1.23 to 2.88) | 0.17 (0.1 to 0.25) | 2.13 (1.35 to 3.21) | 1.83 (1.18 to 2.75) | -0.48 (-0.55 to -0.41) |
| Western Europe | 7305 (4514 to 11136) | 9058 (5684 to 13466) | 0.24 (0.18 to 0.31) | 1.9 (1.17 to 2.9) | 2.08 (1.3 to 3.09) | 0.13 (0.05 to 0.21) | 1.73 (1.03 to 2.72) | 1.68 (0.98 to 2.67) | -0.26 (-0.34 to -0.19) |
| Western Sub-Saharan Africa | 1546 (977 to 2383) | 3641 (2327 to 5617) | 1.36 (1.3 to 1.42) | 0.8 (0.51 to 1.24) | 0.8 (0.51 to 1.23) | -0.02 (-0.12 to 0.09) | 1.09 (0.72 to 1.6) | 1.12 (0.74 to 1.65) | 0.13 (0.06 to 0.21) |

YLDs, years lived with disability; CR, crude rate; ASR, age-standardized rate; EAPC, estimated annual percentage change; UI, uncertainty interval; CI, confidence interval.

Table S3. Incidence of knee dislocation in 1990 and 2019 for both sexes in 204 countries and territories, with EAPC from 1990 and 2019.

| **Location** | **Number** | | | **CR, per 100k** | | | **ASR, per 100k** | | |
| --- | --- | --- | --- | --- | --- | --- | --- | --- | --- |
|  | **Number in 1990 (95% UI)** | **Number in 2019 (95% UI)** | **Number change rate (95% UI)** | **CR in 1990 (95% UI)** | **CR in 2019 (95% UI)** | **EAPC of CR, % per year (95% CI)** | **ASR in 1990 (95% UI)** | **ASR in 2019 (95% UI)** | **EAPC of ASR, % per year (95% CI)** |
| Afghanistan | 4011 (2824 to 5713) | 13807 (9688 to 19882) | 2.44 (2.16 to 2.71) | 35.12 (24.73 to 50.03) | 36.07 (25.31 to 51.94) | -0.76 (-1.71 to 0.2) | 32.89 (23.25 to 46.79) | 33.28 (23.8 to 47.13) | -0.84 (-1.88 to 0.2) |
| Albania | 4046 (2670 to 6230) | 2587 (1705 to 3926) | -0.36 (-0.43 to -0.27) | 122.25 (80.66 to 188.21) | 95.09 (62.69 to 144.31) | -1.2 (-1.35 to -1.05) | 113.91 (74.54 to 176.82) | 106.59 (69.93 to 163.28) | -0.56 (-0.69 to -0.42) |
| Algeria | 11214 (7572 to 16288) | 16257 (10815 to 24203) | 0.45 (0.35 to 0.54) | 44.36 (29.95 to 64.42) | 38.85 (25.84 to 57.84) | -0.75 (-1.07 to -0.43) | 39.85 (27.06 to 56.88) | 38.3 (25.66 to 56.95) | -0.42 (-0.73 to -0.1) |
| American Samoa | 19 (13 to 27) | 20 (14 to 30) | 0.07 (-0.01 to 0.15) | 38.42 (26.11 to 56.3) | 35.81 (24.43 to 54.74) | -0.11 (-1.16 to 0.95) | 37.32 (25.64 to 54.47) | 36.06 (24.7 to 54.53) | 0.05 (-0.99 to 1.09) |
| Andorra | 36 (23 to 57) | 53 (33 to 84) | 0.47 (0.39 to 0.56) | 66.34 (42.25 to 104.77) | 63.66 (40.21 to 101.29) | -0.13 (-0.17 to -0.08) | 70.76 (44.48 to 112.64) | 75.74 (46.31 to 124.09) | 0.19 (0.13 to 0.25) |
| Angola | 2704 (1909 to 3727) | 7486 (5224 to 10544) | 1.77 (1.6 to 1.96) | 26.2 (18.5 to 36.12) | 24.84 (17.33 to 34.99) | -0.23 (-0.3 to -0.16) | 24.1 (16.89 to 33.33) | 22.94 (15.97 to 31.91) | -0.2 (-0.26 to -0.14) |
| Antigua and Barbuda | 26 (18 to 38) | 38 (26 to 57) | 0.47 (0.36 to 0.58) | 42.63 (30.13 to 62.35) | 43.14 (29.42 to 64.04) | -0.19 (-0.48 to 0.11) | 40.76 (28.69 to 59.84) | 45.43 (31.18 to 67.24) | 0.13 (-0.2 to 0.46) |
| Argentina | 23074 (15555 to 34393) | 30024 (20205 to 44993) | 0.3 (0.26 to 0.35) | 69.67 (46.96 to 103.84) | 66.55 (44.78 to 99.73) | -0.28 (-0.36 to -0.21) | 69.05 (46.67 to 102.67) | 69.27 (46.44 to 104.67) | -0.12 (-0.2 to -0.05) |
| Armenia | 2762 (1894 to 4031) | 1541 (1054 to 2269) | -0.44 (-0.47 to -0.41) | 80.9 (55.47 to 118.04) | 51.04 (34.91 to 75.14) | -1.81 (-2.03 to -1.59) | 77.81 (53.33 to 113.45) | 57.02 (38.68 to 84.67) | -1.27 (-1.52 to -1.02) |
| Australia | 20334 (13463 to 31030) | 27742 (18269 to 42357) | 0.36 (0.29 to 0.44) | 120.6 (79.85 to 184.04) | 112.92 (74.36 to 172.41) | -0.24 (-0.33 to -0.16) | 126.93 (82.96 to 195.62) | 129.4 (82.6 to 202.79) | 0.05 (-0.02 to 0.12) |
| Austria | 6055 (3896 to 9396) | 5840 (3702 to 9344) | -0.04 (-0.1 to 0.02) | 77.94 (50.15 to 120.95) | 65.5 (41.53 to 104.8) | -0.73 (-0.78 to -0.69) | 82.49 (52.26 to 130.55) | 76.41 (47.01 to 124.7) | -0.39 (-0.44 to -0.33) |
| Azerbaijan | 4412 (3026 to 6373) | 5444 (3703 to 8051) | 0.23 (0.17 to 0.29) | 60.18 (41.27 to 86.94) | 52.96 (36.03 to 78.33) | -0.44 (-0.6 to -0.28) | 55.34 (38.1 to 80.08) | 54.3 (36.54 to 80.41) | -0.05 (-0.26 to 0.16) |
| Bahamas | 88 (64 to 122) | 132 (94 to 185) | 0.5 (0.39 to 0.62) | 34.44 (25.04 to 47.65) | 35.1 (25.06 to 49.17) | 0.16 (-0.04 to 0.35) | 32.04 (23.39 to 44.2) | 36.57 (26.12 to 51.57) | 0.52 (0.33 to 0.71) |
| Bahrain | 194 (134 to 279) | 530 (356 to 782) | 1.73 (1.51 to 1.95) | 38.19 (26.45 to 54.84) | 36.73 (24.7 to 54.21) | 0.08 (-0.06 to 0.22) | 34.12 (23.75 to 48.36) | 39.76 (26.71 to 59.25) | 0.62 (0.48 to 0.76) |
| Bangladesh | 32187 (22526 to 45359) | 51442 (34779 to 74799) | 0.6 (0.43 to 0.75) | 29.51 (20.66 to 41.59) | 32.3 (21.84 to 46.97) | -0.99 (-2.63 to 0.68) | 27.44 (19.16 to 38.91) | 31.14 (21.19 to 45.28) | -0.89 (-2.52 to 0.78) |
| Barbados | 81 (59 to 113) | 93 (66 to 132) | 0.15 (0.07 to 0.23) | 31.8 (23.19 to 44.44) | 31.09 (22.22 to 44.21) | -0.11 (-0.17 to -0.05) | 31.82 (23.22 to 44.62) | 36.18 (25.78 to 51.52) | 0.41 (0.35 to 0.47) |
| Belarus | 10398 (6973 to 15344) | 8552 (5598 to 13070) | -0.18 (-0.23 to -0.12) | 99.31 (66.59 to 146.54) | 90.01 (58.92 to 137.57) | -0.04 (-0.33 to 0.24) | 102.85 (68.93 to 152.07) | 99.43 (64.86 to 151.1) | 0.11 (-0.09 to 0.31) |
| Belgium | 7209 (4568 to 11366) | 9042 (5548 to 14844) | 0.25 (0.17 to 0.33) | 72.23 (45.77 to 113.9) | 79.18 (48.58 to 130) | 0.95 (0.55 to 1.36) | 79.42 (49.55 to 126.79) | 87.77 (52.85 to 146.06) | 1.16 (0.66 to 1.66) |
| Belize | 72 (52 to 100) | 181 (128 to 257) | 1.53 (1.36 to 1.7) | 38.48 (27.83 to 53.95) | 44.06 (31.17 to 62.65) | 0.34 (0.04 to 0.64) | 34.14 (24.69 to 47.85) | 41.72 (29.61 to 58.92) | 0.52 (0.21 to 0.84) |
| Benin | 1629 (1130 to 2290) | 4108 (2840 to 5847) | 1.52 (1.41 to 1.64) | 33.56 (23.28 to 47.18) | 32.43 (22.42 to 46.17) | -0.15 (-0.23 to -0.08) | 31.93 (22.13 to 44.85) | 31.92 (22.12 to 44.86) | -0.04 (-0.09 to 0.01) |
| Bermuda | 25 (17 to 36) | 28 (19 to 41) | 0.1 (0.01 to 0.21) | 42.3 (29.28 to 60.85) | 43.32 (30.1 to 63.78) | 0.07 (-0.07 to 0.2) | 44.25 (30.76 to 64.42) | 51.6 (35.43 to 77.34) | 0.52 (0.39 to 0.65) |
| Bhutan | 220 (152 to 310) | 318 (216 to 452) | 0.45 (0.32 to 0.6) | 35.92 (24.87 to 50.71) | 42.2 (28.64 to 59.88) | 0.36 (-0.13 to 0.85) | 33.96 (23.34 to 47.84) | 40.77 (27.69 to 58.36) | 0.39 (-0.1 to 0.87) |
| Bolivia (Plurinational State of) | 2621 (1892 to 3624) | 4566 (3252 to 6569) | 0.74 (0.59 to 0.88) | 40.82 (29.47 to 56.44) | 38.01 (27.08 to 54.69) | -0.45 (-0.53 to -0.38) | 37.35 (27.13 to 52.05) | 36.72 (25.96 to 52.63) | -0.26 (-0.35 to -0.18) |
| Bosnia and Herzegovina | 4468 (2985 to 6643) | 2862 (1900 to 4328) | -0.36 (-0.4 to -0.32) | 98.47 (65.77 to 146.4) | 86.72 (57.58 to 131.16) | -0.22 (-0.35 to -0.1) | 97.79 (65.46 to 146.37) | 105.63 (68.9 to 159.69) | 0.4 (0.29 to 0.52) |
| Botswana | 484 (332 to 688) | 846 (588 to 1196) | 0.75 (0.61 to 0.93) | 37.2 (25.52 to 52.85) | 36.17 (25.13 to 51.16) | -0.15 (-0.22 to -0.09) | 34.14 (23.82 to 48.35) | 35.28 (24.74 to 49.6) | 0 (-0.07 to 0.07) |
| Brazil | 110001 (74571 to 164651) | 124808 (84989 to 187777) | 0.13 (0.07 to 0.2) | 73.91 (50.1 to 110.63) | 57.6 (39.23 to 86.67) | -0.82 (-0.89 to -0.76) | 70.51 (48.31 to 105.22) | 58.38 (39.5 to 88.54) | -0.59 (-0.65 to -0.52) |
| Brunei Darussalam | 199 (134 to 294) | 290 (194 to 431) | 0.46 (0.38 to 0.53) | 76.92 (51.91 to 113.7) | 66.23 (44.33 to 98.52) | -0.54 (-0.61 to -0.46) | 70.15 (47.82 to 103.28) | 65.13 (43.62 to 96.28) | -0.31 (-0.39 to -0.23) |
| Bulgaria | 9713 (6531 to 14356) | 6243 (4122 to 9539) | -0.36 (-0.41 to -0.31) | 111.88 (75.24 to 165.37) | 90.03 (59.44 to 137.55) | -0.78 (-0.81 to -0.74) | 122.79 (82.44 to 180.62) | 112.91 (74.04 to 171.25) | -0.3 (-0.36 to -0.23) |
| Burkina Faso | 3001 (2083 to 4227) | 8076 (5637 to 11287) | 1.69 (1.45 to 2.16) | 31.4 (21.79 to 44.22) | 35.59 (24.84 to 49.74) | 0.21 (0.03 to 0.39) | 30.46 (21.07 to 43.03) | 35.68 (24.97 to 49.87) | 0.34 (0.16 to 0.51) |
| Burundi | 1767 (1225 to 2497) | 3799 (2632 to 5529) | 1.15 (1.02 to 1.29) | 31.73 (22 to 44.82) | 31.83 (22.05 to 46.33) | -0.23 (-0.94 to 0.48) | 30.22 (20.9 to 42.4) | 29.44 (20.47 to 41.79) | -0.37 (-1.1 to 0.36) |
| Cabo Verde | 125 (86 to 175) | 199 (140 to 279) | 0.6 (0.46 to 0.75) | 35.54 (24.48 to 49.87) | 35.38 (24.81 to 49.48) | 0.06 (-0.03 to 0.14) | 32.72 (22.99 to 45.45) | 35.43 (25.11 to 49.41) | 0.33 (0.26 to 0.39) |
| Cambodia | 3016 (2060 to 4463) | 5691 (3709 to 8815) | 0.89 (0.72 to 1.05) | 29.07 (19.85 to 43.02) | 34.28 (22.34 to 53.09) | 0.39 (0.23 to 0.54) | 27.24 (18.73 to 39.55) | 33.56 (21.99 to 51.67) | 0.52 (0.35 to 0.69) |
| Cameroon | 3489 (2391 to 4960) | 10072 (6925 to 14481) | 1.89 (1.73 to 2.08) | 33.58 (23.01 to 47.74) | 34.61 (23.8 to 49.76) | 0.15 (0.05 to 0.25) | 32.79 (22.55 to 46.72) | 34.63 (23.97 to 49.17) | 0.22 (0.14 to 0.3) |
| Canada | 15756 (10916 to 22910) | 20354 (13996 to 29855) | 0.29 (0.21 to 0.38) | 57.81 (40.05 to 84.05) | 55.74 (38.33 to 81.75) | -0.12 (-0.19 to -0.05) | 57.61 (39.95 to 84.34) | 54.95 (37.8 to 81.52) | -0.19 (-0.25 to -0.13) |
| Central African Republic | 628 (445 to 864) | 1225 (880 to 1696) | 0.95 (0.88 to 1.05) | 22.88 (16.22 to 31.5) | 23.11 (16.61 to 31.99) | 0.54 (0.15 to 0.94) | 21.41 (15.2 to 29.4) | 21.81 (15.65 to 29.85) | 0.57 (0.18 to 0.96) |
| Chad | 1921 (1329 to 2692) | 5398 (3718 to 7591) | 1.81 (1.7 to 1.94) | 31.9 (22.06 to 44.71) | 32.92 (22.67 to 46.29) | 0.12 (0.07 to 0.18) | 30.09 (20.78 to 42.64) | 32.2 (22.26 to 45.07) | 0.26 (0.21 to 0.31) |
| Chile | 9060 (6113 to 13741) | 12001 (8009 to 18301) | 0.32 (0.26 to 0.38) | 68.22 (46.03 to 103.47) | 65.95 (44.01 to 100.57) | -0.23 (-0.48 to 0.01) | 65.36 (44.23 to 98.53) | 71.4 (47.3 to 110.11) | 0.28 (0.02 to 0.54) |
| China | 369419 (253237 to 580003) | 509932 (348574 to 776777) | 0.38 (0.23 to 0.57) | 31.21 (21.39 to 49) | 35.85 (24.51 to 54.61) | -0.04 (-0.42 to 0.34) | 30.89 (21.27 to 48.21) | 34.82 (23.93 to 53.15) | -0.12 (-0.49 to 0.25) |
| Colombia | 19473 (13125 to 28542) | 19658 (13246 to 29532) | 0.01 (-0.09 to 0.07) | 59.83 (40.32 to 87.69) | 41.14 (27.72 to 61.81) | -1.49 (-1.61 to -1.37) | 54.51 (37.12 to 79.18) | 43.72 (29.31 to 66.56) | -0.97 (-1.06 to -0.87) |
| Comoros | 206 (142 to 294) | 304 (209 to 435) | 0.48 (0.4 to 0.56) | 44.24 (30.48 to 63.21) | 42.62 (29.32 to 60.86) | -0.24 (-0.36 to -0.12) | 42.75 (29.3 to 61.34) | 41.97 (28.86 to 60.12) | -0.18 (-0.3 to -0.06) |
| Congo | 587 (413 to 810) | 1196 (836 to 1701) | 1.04 (0.95 to 1.13) | 24.01 (16.9 to 33.15) | 22.72 (15.88 to 32.3) | -0.16 (-0.28 to -0.03) | 22.4 (15.73 to 31.18) | 21.6 (15.08 to 30.38) | -0.11 (-0.24 to 0.01) |
| Cook Islands | 7 (5 to 10) | 6 (4 to 10) | -0.1 (-0.21 to 0) | 36.92 (25.92 to 54.56) | 35.11 (24.04 to 54.42) | -0.49 (-1.55 to 0.59) | 37.14 (26.33 to 54.06) | 36.81 (25.07 to 57.48) | -0.28 (-1.45 to 0.9) |
| Costa Rica | 1541 (1018 to 2408) | 2070 (1391 to 3147) | 0.34 (0.27 to 0.42) | 50.69 (33.5 to 79.2) | 43.88 (29.49 to 66.73) | -0.53 (-0.64 to -0.42) | 46.01 (30.82 to 70.61) | 47.13 (31.34 to 73.17) | 0.1 (-0.01 to 0.21) |
| Côte d'Ivoire | 4124 (2855 to 5805) | 8550 (5896 to 12054) | 1.07 (1 to 1.16) | 33.73 (23.35 to 47.48) | 32.67 (22.53 to 46.06) | -0.12 (-0.2 to -0.03) | 33.26 (23.2 to 47.04) | 32.79 (22.8 to 46.05) | -0.08 (-0.17 to 0.01) |
| Croatia | 5517 (3718 to 8011) | 4124 (2688 to 6226) | -0.25 (-0.36 to -0.15) | 112.57 (75.87 to 163.46) | 97.09 (63.29 to 146.56) | -0.34 (-0.42 to -0.26) | 118.12 (79.26 to 170.44) | 109.13 (69.71 to 165.84) | -0.07 (-0.15 to 0.01) |
| Cuba | 4712 (3408 to 6471) | 5470 (3840 to 7832) | 0.16 (0.05 to 0.27) | 43.5 (31.47 to 59.74) | 48.15 (33.81 to 68.95) | 0.39 (0.32 to 0.46) | 42.58 (30.82 to 58.65) | 51.95 (36.61 to 73.91) | 0.73 (0.67 to 0.79) |
| Cyprus | 513 (331 to 804) | 836 (528 to 1340) | 0.63 (0.51 to 0.74) | 65.9 (42.56 to 103.31) | 63.62 (40.22 to 101.99) | -0.13 (-0.21 to -0.06) | 67.45 (43.37 to 105.85) | 73.83 (45.15 to 121.6) | 0.31 (0.26 to 0.36) |
| Czechia | 12260 (8274 to 18079) | 10382 (6906 to 15273) | -0.15 (-0.2 to -0.11) | 119.06 (80.35 to 175.57) | 97.54 (64.89 to 143.5) | -0.56 (-0.65 to -0.47) | 122.31 (82.2 to 179.5) | 115.59 (75.77 to 172.54) | 0.08 (-0.01 to 0.17) |
| Democratic People's Republic of Korea | 4077 (2691 to 6242) | 4199 (2916 to 6007) | 0.03 (-0.09 to 0.19) | 19.36 (12.78 to 29.65) | 16.01 (11.12 to 22.9) | -0.44 (-0.68 to -0.19) | 18.96 (12.51 to 29.4) | 16.05 (11.05 to 22.96) | -0.38 (-0.62 to -0.14) |
| Democratic Republic of the Congo | 9480 (6625 to 13114) | 21322 (15017 to 29613) | 1.25 (1.15 to 1.36) | 24.57 (17.17 to 33.98) | 24.32 (17.13 to 33.78) | -0.02 (-0.18 to 0.15) | 22.37 (15.71 to 31.24) | 22.58 (15.93 to 31.75) | 0.04 (-0.12 to 0.2) |
| Denmark | 3481 (2289 to 5369) | 3544 (2235 to 5661) | 0.02 (-0.05 to 0.09) | 67.67 (44.49 to 104.36) | 61.08 (38.52 to 97.55) | -0.49 (-0.58 to -0.4) | 71.65 (46.08 to 113.73) | 72.76 (44.32 to 122) | -0.01 (-0.11 to 0.09) |
| Djibouti | 197 (137 to 280) | 477 (334 to 687) | 1.42 (1.31 to 1.55) | 40.52 (28.17 to 57.6) | 39.68 (27.81 to 57.09) | -0.3 (-0.63 to 0.04) | 39.04 (27.17 to 55.29) | 39.45 (27.7 to 56.36) | -0.23 (-0.59 to 0.14) |
| Dominica | 25 (18 to 34) | 23 (17 to 32) | -0.07 (-0.12 to -0.01) | 33.5 (24.73 to 45.64) | 33.69 (24.22 to 47.15) | 0.66 (0.05 to 1.26) | 31.91 (23.55 to 43.56) | 35.84 (25.81 to 50.22) | 0.93 (0.4 to 1.46) |
| Dominican Republic | 2559 (1887 to 3536) | 4580 (3281 to 6361) | 0.79 (0.67 to 0.9) | 35.52 (26.2 to 49.1) | 42.09 (30.15 to 58.45) | 0.6 (0.39 to 0.82) | 32.05 (23.59 to 44.24) | 41.14 (29.35 to 57.05) | 0.87 (0.64 to 1.09) |
| Ecuador | 4306 (3075 to 6060) | 7710 (5345 to 11072) | 0.79 (0.66 to 0.91) | 42.95 (30.67 to 60.44) | 43.83 (30.39 to 62.95) | -0.02 (-0.18 to 0.14) | 39.38 (28.24 to 54.61) | 42.72 (29.67 to 60.91) | 0.19 (0.03 to 0.35) |
| Egypt | 17365 (12057 to 24826) | 32761 (21999 to 48913) | 0.89 (0.73 to 1.04) | 31.18 (21.65 to 44.57) | 33.07 (22.21 to 49.37) | 0.19 (0 to 0.38) | 28.07 (19.58 to 40.01) | 31.05 (20.98 to 45.94) | 0.33 (0.13 to 0.53) |
| El Salvador | 2586 (1765 to 3831) | 2831 (1900 to 4203) | 0.09 (0.01 to 0.18) | 49.09 (33.5 to 72.74) | 45.25 (30.37 to 67.19) | -0.42 (-0.75 to -0.08) | 43.12 (29.75 to 62.98) | 44.99 (30.08 to 66.61) | 0.01 (-0.34 to 0.36) |
| Equatorial Guinea | 106 (76 to 146) | 349 (244 to 493) | 2.28 (1.96 to 2.6) | 24.75 (17.67 to 33.9) | 24.58 (17.18 to 34.73) | 0.06 (-0.05 to 0.16) | 22.68 (16.09 to 31.29) | 22.55 (15.71 to 31.69) | 0.04 (-0.08 to 0.16) |
| Eritrea | 1195 (832 to 1716) | 2705 (1878 to 3929) | 1.26 (1.16 to 1.38) | 39.82 (27.71 to 57.16) | 40.3 (27.98 to 58.54) | 0.02 (-0.04 to 0.09) | 39.3 (27.32 to 56.85) | 39.81 (27.76 to 57.37) | 0.01 (-0.05 to 0.06) |
| Estonia | 1794 (1211 to 2649) | 1001 (667 to 1492) | -0.44 (-0.48 to -0.41) | 114.37 (77.22 to 168.84) | 76.26 (50.82 to 113.7) | -1.55 (-1.66 to -1.44) | 118.45 (79.62 to 173.48) | 88.64 (58.51 to 131.06) | -1.16 (-1.26 to -1.06) |
| Eswatini | 278 (192 to 392) | 401 (278 to 565) | 0.44 (0.36 to 0.54) | 34.43 (23.76 to 48.61) | 35.07 (24.34 to 49.43) | 0.14 (0.1 to 0.19) | 31.65 (22.08 to 44.48) | 33.4 (23.41 to 47.06) | 0.23 (0.16 to 0.3) |
| Ethiopia | 17956 (12707 to 25031) | 33372 (23348 to 47709) | 0.86 (0.73 to 1) | 34.94 (24.73 to 48.71) | 31.02 (21.7 to 44.34) | -0.39 (-0.63 to -0.14) | 33.49 (23.64 to 46.07) | 28.7 (20.33 to 40.63) | -0.54 (-0.78 to -0.31) |
| Fiji | 185 (126 to 275) | 226 (148 to 347) | 0.22 (0.14 to 0.3) | 24.32 (16.54 to 36.17) | 24.76 (16.26 to 38.1) | 0.04 (-0.29 to 0.38) | 22.5 (15.55 to 33.15) | 24.46 (16.1 to 37.67) | 0.27 (-0.07 to 0.61) |
| Finland | 4810 (3023 to 7714) | 4910 (3007 to 8115) | 0.02 (-0.03 to 0.07) | 96 (60.34 to 153.97) | 88.71 (54.34 to 146.63) | -0.22 (-0.59 to 0.15) | 104.82 (64.6 to 170.7) | 106.15 (62.31 to 181.11) | 0.13 (-0.24 to 0.5) |
| France | 45390 (29319 to 70553) | 49052 (30875 to 79024) | 0.08 (0.01 to 0.14) | 78.57 (50.75 to 122.13) | 74.09 (46.64 to 119.36) | -0.26 (-0.3 to -0.23) | 81.86 (52.22 to 129.12) | 81.19 (49.55 to 133.81) | -0.09 (-0.13 to -0.05) |
| Gabon | 262 (184 to 362) | 435 (303 to 614) | 0.66 (0.59 to 0.74) | 26.38 (18.54 to 36.47) | 24.88 (17.34 to 35.1) | -0.21 (-0.29 to -0.12) | 24.7 (17.33 to 34.36) | 24.19 (16.82 to 33.76) | -0.1 (-0.18 to -0.01) |
| Gambia | 318 (220 to 450) | 716 (496 to 1012) | 1.25 (1.15 to 1.35) | 32.08 (22.21 to 45.32) | 31.87 (22.06 to 45.07) | 0.04 (-0.09 to 0.17) | 30.08 (20.94 to 42.52) | 31.17 (21.75 to 44.26) | 0.16 (0.04 to 0.29) |
| Georgia | 4141 (2766 to 6096) | 2664 (1767 to 3995) | -0.36 (-0.39 to -0.33) | 75.18 (50.22 to 110.66) | 72.68 (48.21 to 109) | 0.06 (-0.17 to 0.3) | 76.83 (51.45 to 112.69) | 82.95 (55.1 to 125.59) | 0.43 (0.18 to 0.69) |
| Germany | 49509 (31903 to 76436) | 51037 (32520 to 80896) | 0.03 (-0.03 to 0.08) | 61.93 (39.91 to 95.61) | 60.1 (38.3 to 95.27) | -0.27 (-0.42 to -0.11) | 69.11 (44.04 to 110.19) | 72.06 (44 to 118.55) | 0.04 (-0.08 to 0.16) |
| Ghana | 4944 (3382 to 7111) | 11167 (7640 to 16235) | 1.26 (1.13 to 1.42) | 32.92 (22.52 to 47.36) | 35.41 (24.23 to 51.48) | 0.31 (0.22 to 0.39) | 31.66 (21.77 to 45.06) | 35.08 (23.96 to 50.31) | 0.37 (0.29 to 0.45) |
| Greece | 6780 (4333 to 10523) | 5452 (3481 to 8564) | -0.2 (-0.24 to -0.15) | 65.26 (41.7 to 101.29) | 52.74 (33.67 to 82.84) | -0.88 (-0.95 to -0.8) | 72.33 (45.78 to 113.81) | 68.31 (42.35 to 112.5) | -0.3 (-0.35 to -0.24) |
| Greenland | 72 (49 to 106) | 53 (36 to 78) | -0.27 (-0.32 to -0.21) | 129.58 (87.75 to 191.42) | 94.19 (64.28 to 139.25) | -1.18 (-1.28 to -1.08) | 126.97 (87.14 to 186.83) | 92.49 (63.2 to 137.66) | -1.27 (-1.34 to -1.2) |
| Grenada | 33 (24 to 46) | 44 (31 to 63) | 0.35 (0.24 to 0.45) | 38.02 (27.57 to 54.06) | 42.58 (30.24 to 61.11) | 0.34 (0.05 to 0.62) | 35.94 (25.98 to 50.73) | 43.92 (31.27 to 63.25) | 0.66 (0.37 to 0.94) |
| Guam | 48 (33 to 71) | 57 (39 to 88) | 0.18 (0.1 to 0.28) | 35.4 (24.07 to 52.16) | 33.57 (22.84 to 51.59) | -0.33 (-0.45 to -0.21) | 33.69 (23.06 to 49.57) | 34.29 (23.23 to 52.75) | -0.08 (-0.19 to 0.03) |
| Guatemala | 4624 (3165 to 6874) | 10066 (6839 to 15209) | 1.18 (0.98 to 1.4) | 58.04 (39.73 to 86.29) | 56.62 (38.47 to 85.56) | -0.05 (-0.15 to 0.05) | 49.8 (34.65 to 71.95) | 53.07 (36.07 to 79.48) | 0.25 (0.15 to 0.36) |
| Guinea | 2101 (1455 to 2961) | 4144 (2864 to 5917) | 0.97 (0.86 to 1.09) | 33.97 (23.52 to 47.86) | 32.77 (22.65 to 46.8) | -0.08 (-0.26 to 0.1) | 31.76 (21.82 to 45.09) | 32.58 (22.61 to 45.96) | 0.15 (-0.04 to 0.34) |
| Guinea-Bissau | 373 (255 to 530) | 658 (450 to 945) | 0.77 (0.68 to 0.85) | 37.03 (25.32 to 52.63) | 34.63 (23.68 to 49.69) | -0.25 (-0.3 to -0.2) | 35.87 (24.65 to 50.64) | 34.3 (23.42 to 48.76) | -0.17 (-0.21 to -0.13) |
| Guyana | 349 (258 to 481) | 369 (267 to 513) | 0.06 (-0.03 to 0.13) | 45.35 (33.57 to 62.47) | 47.86 (34.61 to 66.54) | 0.01 (-0.11 to 0.12) | 41.28 (30.65 to 56.56) | 46.82 (33.84 to 65.25) | 0.27 (0.15 to 0.38) |
| Haiti | 2535 (1842 to 3594) | 4753 (3419 to 6786) | 0.88 (0.77 to 0.99) | 39.88 (28.98 to 56.55) | 38.33 (27.57 to 54.72) | 0.54 (-2.19 to 3.36) | 36.81 (26.67 to 52.52) | 36.04 (25.99 to 51.18) | 0.62 (-2.18 to 3.51) |
| Honduras | 2432 (1680 to 3547) | 4570 (3130 to 6733) | 0.88 (0.75 to 1.03) | 51.64 (35.69 to 75.32) | 46.56 (31.89 to 68.6) | -1.17 (-2.6 to 0.29) | 43.58 (30.5 to 63.02) | 43.21 (29.85 to 63.79) | -0.87 (-2.37 to 0.66) |
| Hungary | 11983 (8006 to 17792) | 8884 (5800 to 13395) | -0.26 (-0.3 to -0.22) | 115.31 (77.04 to 171.19) | 91.83 (59.95 to 138.46) | -1.05 (-1.15 to -0.94) | 117.92 (78.54 to 173.87) | 108.29 (69.86 to 165.1) | -0.49 (-0.61 to -0.37) |
| Iceland | 174 (112 to 274) | 223 (140 to 358) | 0.28 (0.2 to 0.35) | 68.53 (43.91 to 108) | 64.6 (40.52 to 103.92) | -0.32 (-0.46 to -0.19) | 69.66 (44.4 to 111.29) | 72.69 (44.35 to 120.24) | 0.03 (-0.1 to 0.15) |
| India | 435232 (291627 to 653461) | 682151 (454212 to 1010654) | 0.57 (0.46 to 0.7) | 50.87 (34.09 to 76.38) | 49.05 (32.66 to 72.67) | -0.21 (-0.31 to -0.12) | 50.25 (34.23 to 74.95) | 48.64 (32.54 to 72.06) | -0.2 (-0.3 to -0.11) |
| Indonesia | 87027 (58684 to 126605) | 101804 (68572 to 149097) | 0.17 (0.12 to 0.22) | 46.95 (31.66 to 68.3) | 39.24 (26.43 to 57.46) | -0.67 (-0.98 to -0.37) | 44.82 (30.93 to 64.71) | 39.96 (27.16 to 58.68) | -0.48 (-0.79 to -0.16) |
| Iran (Islamic Republic of) | 55090 (35546 to 82513) | 31041 (21179 to 44880) | -0.44 (-0.64 to -0.2) | 94.11 (60.72 to 140.95) | 36.82 (25.12 to 53.24) | -1.62 (-2.19 to -1.06) | 96.51 (60.66 to 150.96) | 36.91 (25.29 to 53.78) | -1.39 (-2.05 to -0.72) |
| Iraq | 10375 (7038 to 15135) | 22663 (15510 to 33392) | 1.18 (1.01 to 1.34) | 58.96 (40 to 86.01) | 53.81 (36.82 to 79.28) | -0.39 (-0.45 to -0.33) | 54.96 (37.46 to 80.62) | 49.04 (33.66 to 72.09) | -0.48 (-0.54 to -0.42) |
| Ireland | 2476 (1594 to 3919) | 3168 (1980 to 5184) | 0.28 (0.19 to 0.37) | 68.75 (44.25 to 108.83) | 64.51 (40.33 to 105.57) | -0.28 (-0.36 to -0.2) | 70.07 (45.19 to 111.64) | 73.56 (45.01 to 124.52) | 0.08 (0 to 0.16) |
| Israel | 3220 (2088 to 5157) | 5977 (3688 to 9845) | 0.86 (0.69 to 1) | 64.89 (42.08 to 103.92) | 64.2 (39.61 to 105.76) | -0.04 (-0.13 to 0.04) | 62.89 (40.96 to 100.19) | 65.92 (40.56 to 108.74) | 0.16 (0.08 to 0.24) |
| Italy | 38486 (25342 to 59329) | 29760 (18988 to 46921) | -0.23 (-0.29 to -0.17) | 67.76 (44.62 to 104.46) | 49.34 (31.48 to 77.8) | -1.93 (-2.3 to -1.56) | 72.24 (46.86 to 112.49) | 59.72 (37.18 to 97.5) | -1.47 (-1.85 to -1.09) |
| Jamaica | 983 (700 to 1409) | 1091 (767 to 1578) | 0.11 (0.04 to 0.19) | 41.57 (29.61 to 59.63) | 38.8 (27.3 to 56.16) | -0.38 (-0.48 to -0.28) | 38.59 (27.37 to 55.97) | 39.7 (28.03 to 57.58) | -0.06 (-0.17 to 0.04) |
| Japan | 97666 (65314 to 146773) | 84057 (57647 to 123770) | -0.14 (-0.18 to -0.08) | 77.59 (51.89 to 116.61) | 65.78 (45.11 to 96.86) | -0.64 (-0.76 to -0.52) | 82.52 (54.85 to 123.12) | 77.2 (51.06 to 117.93) | -0.28 (-0.41 to -0.15) |
| Jordan | 1491 (1024 to 2176) | 4420 (2908 to 6709) | 1.96 (1.73 to 2.2) | 39.53 (27.14 to 57.66) | 37.98 (24.99 to 57.65) | -0.26 (-0.36 to -0.17) | 33.96 (23.54 to 48.67) | 34.91 (23.29 to 52.77) | -0.03 (-0.14 to 0.07) |
| Kazakhstan | 12514 (8387 to 18297) | 12698 (8450 to 18630) | 0.01 (-0.03 to 0.06) | 76.45 (51.24 to 111.79) | 69.04 (45.94 to 101.3) | -0.13 (-0.24 to -0.02) | 72.93 (49.21 to 106.7) | 69.56 (46.04 to 102.24) | 0.04 (-0.04 to 0.13) |
| Kenya | 7238 (5083 to 10250) | 15262 (10717 to 21758) | 1.11 (1.04 to 1.18) | 31.21 (21.91 to 44.19) | 30.39 (21.34 to 43.32) | -0.07 (-0.21 to 0.07) | 28.42 (20.14 to 39.62) | 28.66 (20.35 to 40.46) | 0.03 (-0.09 to 0.15) |
| Kiribati | 15 (10 to 22) | 26 (17 to 40) | 0.77 (0.64 to 0.91) | 19.9 (13.7 to 29.45) | 22.01 (14.67 to 33.84) | 0.38 (0.19 to 0.58) | 18.35 (12.78 to 26.8) | 20.47 (13.78 to 31.22) | 0.37 (0.18 to 0.57) |
| Kuwait | 828 (548 to 1237) | 1942 (1266 to 2961) | 1.34 (1.21 to 1.48) | 47.08 (31.17 to 70.31) | 43.88 (28.6 to 66.88) | -0.22 (-0.35 to -0.09) | 41.43 (27.75 to 61.4) | 43.27 (28.22 to 66.38) | 0.08 (0.01 to 0.16) |
| Kyrgyzstan | 3200 (2136 to 4820) | 3655 (2457 to 5379) | 0.14 (0.06 to 0.22) | 71.72 (47.87 to 108.02) | 55.92 (37.59 to 82.31) | -1.06 (-1.21 to -0.91) | 66.67 (44.51 to 99.1) | 53.14 (35.75 to 78.04) | -0.96 (-1.13 to -0.78) |
| Lao People's Democratic Republic | 1194 (851 to 1683) | 1937 (1336 to 2772) | 0.62 (0.52 to 0.73) | 28.76 (20.5 to 40.53) | 27.06 (18.67 to 38.73) | -0.31 (-0.43 to -0.2) | 27.15 (19.26 to 37.84) | 25.86 (17.81 to 37.05) | -0.29 (-0.42 to -0.16) |
| Latvia | 3347 (2219 to 5001) | 1495 (1006 to 2242) | -0.55 (-0.58 to -0.53) | 125.88 (83.48 to 188.09) | 78.07 (52.54 to 117.08) | -1.78 (-1.93 to -1.62) | 129.52 (85.76 to 193.37) | 89.94 (59.85 to 132.69) | -1.41 (-1.53 to -1.28) |
| Lebanon | 1285 (890 to 1853) | 2003 (1338 to 3076) | 0.56 (0.36 to 0.75) | 39.24 (27.18 to 56.58) | 38.68 (25.85 to 59.41) | -0.07 (-0.29 to 0.14) | 37.11 (25.87 to 53.29) | 39.28 (26.38 to 60.21) | 0.13 (-0.09 to 0.35) |
| Lesotho | 637 (446 to 893) | 782 (547 to 1087) | 0.23 (0.14 to 0.33) | 35.25 (24.7 to 49.39) | 37.38 (26.16 to 51.97) | 0.35 (0.22 to 0.47) | 32.79 (23.13 to 45.06) | 35.98 (25.56 to 49.83) | 0.45 (0.33 to 0.57) |
| Liberia | 624 (432 to 880) | 1399 (968 to 1977) | 1.24 (1.13 to 1.35) | 31.78 (21.99 to 44.78) | 29.2 (20.2 to 41.27) | -0.31 (-0.45 to -0.16) | 29.82 (20.57 to 42.22) | 28.85 (19.98 to 41.08) | -0.1 (-0.23 to 0.03) |
| Libya | 1754 (1197 to 2526) | 2864 (1965 to 4174) | 0.63 (0.52 to 0.77) | 41.4 (28.26 to 59.62) | 42.51 (29.18 to 61.97) | 0.61 (0.01 to 1.2) | 37.92 (25.96 to 53.81) | 40.72 (28.12 to 59.46) | 0.72 (0.13 to 1.31) |
| Lithuania | 4306 (2868 to 6357) | 2371 (1580 to 3559) | -0.45 (-0.48 to -0.42) | 117.21 (78.07 to 173.05) | 84.85 (56.53 to 127.36) | -1.06 (-1.19 to -0.93) | 118.25 (78.69 to 174.38) | 94.4 (62.18 to 139.09) | -0.74 (-0.84 to -0.65) |
| Luxembourg | 300 (197 to 456) | 417 (262 to 667) | 0.39 (0.26 to 0.5) | 78.69 (51.7 to 119.62) | 67.43 (42.31 to 107.81) | -0.73 (-0.79 to -0.66) | 85.39 (55.29 to 132.47) | 77.43 (47.43 to 127.62) | -0.54 (-0.63 to -0.45) |
| Madagascar | 5078 (3485 to 7315) | 10782 (7396 to 15596) | 1.12 (1.03 to 1.24) | 42.49 (29.16 to 61.2) | 40.4 (27.71 to 58.43) | -0.25 (-0.37 to -0.12) | 39.95 (27.57 to 57.62) | 38.25 (26.42 to 55.72) | -0.23 (-0.34 to -0.12) |
| Malawi | 3403 (2315 to 4870) | 6847 (4687 to 9937) | 1.01 (0.91 to 1.13) | 35.61 (24.23 to 50.96) | 37.13 (25.41 to 53.88) | 0.02 (-0.17 to 0.2) | 33.36 (22.84 to 48.53) | 34.61 (23.94 to 49.59) | -0.04 (-0.22 to 0.14) |
| Malaysia | 5131 (3531 to 7276) | 9562 (6507 to 13614) | 0.86 (0.74 to 0.99) | 29.06 (20 to 41.21) | 30.55 (20.79 to 43.49) | 0.02 (-0.12 to 0.17) | 27.8 (19.31 to 39.24) | 30.33 (20.7 to 43.56) | 0.12 (-0.01 to 0.25) |
| Maldives | 63 (43 to 90) | 138 (95 to 203) | 1.2 (0.98 to 1.42) | 28.34 (19.47 to 40.67) | 27.74 (18.97 to 40.69) | -0.27 (-1.13 to 0.6) | 26.44 (18.44 to 37.32) | 27.47 (18.76 to 40.86) | -0.07 (-0.96 to 0.83) |
| Mali | 2907 (2011 to 4083) | 7068 (4890 to 9853) | 1.43 (1.31 to 1.55) | 33.52 (23.19 to 47.08) | 32.25 (22.31 to 44.95) | -0.2 (-0.31 to -0.09) | 31.76 (22.01 to 44.99) | 31.44 (22.17 to 43.76) | -0.08 (-0.18 to 0.01) |
| Malta | 260 (162 to 414) | 286 (178 to 463) | 0.1 (0.04 to 0.16) | 70.01 (43.76 to 111.68) | 65.18 (40.41 to 105.32) | -0.01 (-0.08 to 0.07) | 75.02 (46.03 to 121.05) | 82.38 (49.07 to 139.43) | 0.69 (0.59 to 0.8) |
| Marshall Islands | 11 (8 to 17) | 15 (10 to 23) | 0.35 (0.26 to 0.45) | 24.57 (17.08 to 36.15) | 26.66 (17.76 to 40.22) | 0.28 (0.21 to 0.36) | 23.39 (16.51 to 33.83) | 25.6 (17.24 to 38.32) | 0.29 (0.2 to 0.39) |
| Mauritania | 771 (527 to 1112) | 1368 (935 to 1960) | 0.77 (0.7 to 0.86) | 37.31 (25.5 to 53.8) | 34.08 (23.3 to 48.84) | -0.3 (-0.45 to -0.16) | 36.05 (24.55 to 51.81) | 33.51 (22.92 to 47.77) | -0.25 (-0.41 to -0.1) |
| Mauritius | 363 (252 to 528) | 443 (307 to 647) | 0.22 (0.12 to 0.33) | 33.01 (22.88 to 47.99) | 34.71 (24.02 to 50.67) | 0.18 (0.13 to 0.24) | 31.92 (22.22 to 45.9) | 37.25 (25.73 to 54.47) | 0.55 (0.49 to 0.61) |
| Mexico | 64104 (43279 to 97348) | 71138 (48468 to 107388) | 0.11 (0.06 to 0.17) | 74.99 (50.63 to 113.87) | 56.94 (38.79 to 85.95) | 0.87 (0.34 to 1.4) | 69.97 (47.89 to 104.88) | 57.33 (38.96 to 86.19) | 1.05 (0.55 to 1.54) |
| Micronesia (Federated States of) | 26 (18 to 38) | 28 (19 to 44) | 0.11 (0.01 to 0.2) | 24.63 (17.28 to 36.12) | 27.82 (18.48 to 42.94) | 0.4 (-0.36 to 1.16) | 23.59 (16.6 to 33.74) | 26.88 (18.12 to 40.98) | 0.42 (-0.35 to 1.2) |
| Monaco | 19 (13 to 30) | 26 (18 to 40) | 0.37 (0.3 to 0.43) | 63.48 (43.15 to 97.61) | 70.34 (47.48 to 107.54) | 0.39 (0.34 to 0.44) | 74.39 (48.8 to 117.31) | 83.63 (53.69 to 134.8) | 0.45 (0.38 to 0.52) |
| Mongolia | 1435 (1002 to 2079) | 2446 (1641 to 3621) | 0.7 (0.59 to 0.82) | 66.64 (46.52 to 96.54) | 72.2 (48.43 to 106.9) | 0.56 (0.41 to 0.71) | 60.74 (42.64 to 88.61) | 70.26 (46.71 to 104.04) | 0.78 (0.69 to 0.88) |
| Montenegro | 670 (447 to 984) | 596 (395 to 884) | -0.11 (-0.15 to -0.07) | 107.02 (71.49 to 157.22) | 96.08 (63.67 to 142.54) | -0.41 (-0.48 to -0.33) | 106.84 (71.29 to 157.29) | 108.31 (70.37 to 162.65) | 0 (-0.08 to 0.09) |
| Morocco | 11823 (8036 to 17141) | 15966 (10417 to 23852) | 0.35 (0.24 to 0.46) | 46.74 (31.77 to 67.76) | 44.41 (28.97 to 66.34) | -0.37 (-0.5 to -0.23) | 42.1 (28.8 to 60.82) | 43.74 (28.71 to 65.19) | -0.06 (-0.2 to 0.09) |
| Mozambique | 5505 (3743 to 8043) | 14210 (9856 to 20830) | 1.58 (1.38 to 1.88) | 42.12 (28.64 to 61.53) | 48.12 (33.38 to 70.54) | 0.25 (0.08 to 0.42) | 40.31 (27.51 to 59.26) | 46.9 (32.64 to 67.46) | 0.33 (0.17 to 0.48) |
| Myanmar | 15893 (11000 to 22921) | 18636 (12363 to 27674) | 0.17 (0.06 to 0.28) | 38.67 (26.76 to 55.76) | 34.08 (22.61 to 50.61) | 0.08 (-1.53 to 1.71) | 35.55 (24.74 to 50.98) | 33.92 (22.5 to 50.28) | 0.36 (-1.27 to 2.02) |
| Namibia | 507 (351 to 713) | 858 (597 to 1211) | 0.69 (0.6 to 0.8) | 35.94 (24.87 to 50.56) | 35.71 (24.83 to 50.41) | -0.05 (-0.16 to 0.06) | 34.02 (23.85 to 47.69) | 34.21 (23.9 to 48.19) | -0.04 (-0.16 to 0.08) |
| Nauru | 3 (2 to 5) | 4 (3 to 6) | 0.17 (0.09 to 0.27) | 32.31 (21.97 to 47.93) | 36.83 (24.86 to 55.95) | 0.41 (0.35 to 0.48) | 31.62 (21.63 to 46.22) | 35.39 (24.32 to 53.06) | 0.33 (0.27 to 0.4) |
| Nepal | 8764 (5776 to 13528) | 14231 (9146 to 22007) | 0.62 (0.54 to 0.7) | 44.86 (29.57 to 69.25) | 46.79 (30.07 to 72.35) | 0.27 (-0.31 to 0.86) | 43.1 (28.67 to 65.48) | 44.83 (28.98 to 69.17) | 0.26 (-0.32 to 0.85) |
| Netherlands | 7624 (5070 to 11588) | 9638 (6238 to 15140) | 0.26 (0.17 to 0.36) | 51.09 (33.97 to 77.65) | 56.17 (36.36 to 88.24) | 0.09 (-0.19 to 0.38) | 54.97 (36.34 to 86.13) | 60.58 (38.4 to 96.75) | 0.08 (-0.13 to 0.3) |
| New Zealand | 4802 (3230 to 7250) | 5959 (3964 to 9117) | 0.24 (0.15 to 0.35) | 140.51 (94.51 to 212.14) | 132.54 (88.17 to 202.81) | -0.22 (-0.28 to -0.16) | 144.43 (96.47 to 219.14) | 151.31 (98.99 to 237.03) | 0.12 (0.03 to 0.21) |
| Nicaragua | 1900 (1291 to 2838) | 2724 (1811 to 4208) | 0.43 (0.33 to 0.53) | 48.88 (33.22 to 73.01) | 41.84 (27.81 to 64.64) | -0.84 (-1.42 to -0.26) | 40.17 (27.79 to 58.98) | 39.95 (26.71 to 61.63) | -0.38 (-1.15 to 0.39) |
| Niger | 2758 (1916 to 3859) | 7463 (5181 to 10472) | 1.71 (1.6 to 1.81) | 34.37 (23.88 to 48.1) | 32.04 (22.24 to 44.95) | -0.34 (-0.46 to -0.21) | 32.24 (22.65 to 45.33) | 31.29 (21.91 to 43.87) | -0.15 (-0.26 to -0.05) |
| Nigeria | 30444 (20960 to 42922) | 69789 (47903 to 97916) | 1.29 (1.2 to 1.38) | 33.76 (23.24 to 47.59) | 32.49 (22.3 to 45.58) | -0.09 (-0.21 to 0.04) | 31.9 (22.19 to 44.83) | 31.11 (21.79 to 43.81) | -0.03 (-0.15 to 0.1) |
| Niue | 1 (0 to 1) | 1 (0 to 1) | -0.22 (-0.28 to -0.15) | 30.79 (21.04 to 45.5) | 33.61 (23.04 to 51.19) | 0.25 (-0.52 to 1.03) | 31.16 (21.33 to 45.88) | 34.86 (23.71 to 53.89) | 0.33 (-0.44 to 1.12) |
| North Macedonia | 2063 (1396 to 3014) | 1876 (1260 to 2812) | -0.09 (-0.25 to 0.04) | 102.36 (69.28 to 149.55) | 87.14 (58.52 to 130.61) | -0.64 (-0.72 to -0.56) | 101.48 (68.81 to 148.06) | 99.88 (65.78 to 149.67) | -0.16 (-0.25 to -0.06) |
| Northern Mariana Islands | 20 (14 to 30) | 17 (12 to 25) | -0.17 (-0.25 to -0.08) | 44.79 (30.47 to 66.36) | 39.64 (27.4 to 59.97) | -0.65 (-0.86 to -0.44) | 42.28 (28.94 to 62.15) | 42.08 (28.99 to 64.03) | -0.09 (-0.27 to 0.08) |
| Norway | 3871 (2650 to 5850) | 4678 (3152 to 7153) | 0.21 (0.14 to 0.27) | 91.14 (62.4 to 137.74) | 87.46 (58.92 to 133.74) | -0.26 (-0.33 to -0.2) | 91.14 (61.38 to 138.79) | 89.31 (58.98 to 140.96) | -0.18 (-0.26 to -0.09) |
| Oman | 955 (648 to 1380) | 2318 (1542 to 3408) | 1.43 (1.2 to 1.67) | 49.15 (33.34 to 70.99) | 50.57 (33.64 to 74.35) | 0.09 (-0.02 to 0.2) | 46.33 (31.73 to 66.44) | 47.17 (31.25 to 68.9) | -0.05 (-0.14 to 0.05) |
| Pakistan | 29035 (20354 to 41108) | 69989 (47541 to 102257) | 1.41 (1.23 to 1.6) | 25.73 (18.04 to 36.43) | 31.24 (21.22 to 45.64) | 0.67 (0.26 to 1.09) | 25 (17.43 to 35.26) | 29.65 (20.18 to 42.86) | 0.59 (0.15 to 1.03) |
| Palau | 8 (6 to 12) | 10 (7 to 15) | 0.19 (0.12 to 0.26) | 53.14 (36.37 to 78.36) | 53.99 (37.49 to 80.76) | -0.07 (-0.13 to -0.01) | 51.56 (35.8 to 75.16) | 55.78 (38.15 to 84.41) | 0.17 (0.13 to 0.2) |
| Palestine | 907 (609 to 1359) | 2256 (1520 to 3350) | 1.49 (1.36 to 1.66) | 43.82 (29.42 to 65.63) | 45.52 (30.66 to 67.59) | 0.09 (-0.16 to 0.35) | 38.24 (25.89 to 56.11) | 40.57 (27.75 to 60.42) | 0.15 (-0.12 to 0.43) |
| Panama | 1137 (780 to 1684) | 1784 (1203 to 2729) | 0.57 (0.49 to 0.65) | 47.6 (32.66 to 70.5) | 42.89 (28.92 to 65.6) | -0.43 (-0.52 to -0.34) | 43.6 (30.16 to 64.19) | 43.23 (29.04 to 66.05) | -0.11 (-0.2 to -0.02) |
| Papua New Guinea | 1046 (692 to 1581) | 3087 (1987 to 4818) | 1.95 (1.74 to 2.14) | 25.6 (16.93 to 38.67) | 31.28 (20.13 to 48.83) | 0.1 (-0.87 to 1.09) | 24.44 (16.54 to 36.76) | 30.13 (19.52 to 47.13) | 0.12 (-0.89 to 1.13) |
| Paraguay | 2118 (1408 to 3257) | 3310 (2232 to 4985) | 0.56 (0.49 to 0.64) | 52.36 (34.8 to 80.52) | 47.76 (32.2 to 71.94) | -0.34 (-0.43 to -0.25) | 47.08 (32.06 to 70.5) | 45.86 (30.9 to 69.32) | -0.12 (-0.23 to -0.02) |
| Peru | 9165 (6703 to 12443) | 13240 (9281 to 18866) | 0.44 (0.29 to 0.59) | 42.18 (30.85 to 57.26) | 38.95 (27.3 to 55.5) | -0.23 (-0.32 to -0.15) | 38.55 (28.4 to 52.71) | 38.77 (27.21 to 55.55) | 0.06 (-0.03 to 0.15) |
| Philippines | 21704 (15451 to 30673) | 32704 (22763 to 47952) | 0.51 (0.26 to 0.67) | 34.29 (24.41 to 48.46) | 29.16 (20.3 to 42.76) | -0.3 (-0.78 to 0.18) | 32.51 (23.36 to 45.89) | 28.26 (19.77 to 41.12) | -0.19 (-0.69 to 0.32) |
| Poland | 41989 (28173 to 61657) | 36047 (23717 to 53889) | -0.14 (-0.21 to -0.08) | 110.04 (73.83 to 161.58) | 93.79 (61.71 to 140.21) | -0.58 (-0.65 to -0.52) | 112.24 (75.57 to 164.91) | 106.22 (69.2 to 159.95) | -0.23 (-0.35 to -0.11) |
| Portugal | 6175 (4073 to 9199) | 4268 (2816 to 6467) | -0.31 (-0.36 to -0.26) | 60.91 (40.18 to 90.75) | 40.07 (26.44 to 60.71) | -1.85 (-1.98 to -1.72) | 64.17 (42.18 to 96.49) | 47.95 (31.12 to 75.97) | -1.39 (-1.51 to -1.26) |
| Puerto Rico | 1864 (1295 to 2692) | 1746 (1221 to 2560) | -0.06 (-0.15 to 0.03) | 51.58 (35.85 to 74.5) | 49.59 (34.67 to 72.7) | 0.31 (-0.22 to 0.84) | 51.58 (35.86 to 74.65) | 55.45 (38.14 to 80.31) | 0.68 (0.16 to 1.2) |
| Qatar | 291 (198 to 426) | 1851 (1218 to 2861) | 5.36 (4.75 to 5.93) | 65.44 (44.46 to 95.6) | 64.63 (42.54 to 99.87) | 0.35 (0.15 to 0.55) | 56.38 (38.52 to 81.06) | 55.33 (36.46 to 83.82) | 0.07 (-0.04 to 0.19) |
| Republic of Korea | 38504 (26053 to 56669) | 35049 (23543 to 52549) | -0.09 (-0.15 to -0.02) | 86.84 (58.76 to 127.81) | 65.64 (44.09 to 98.41) | -1.26 (-1.37 to -1.16) | 81.49 (55.25 to 120.04) | 74.33 (49.16 to 112.99) | -0.61 (-0.73 to -0.5) |
| Republic of Moldova | 4238 (2894 to 6028) | 2516 (1695 to 3644) | -0.41 (-0.45 to -0.37) | 95.32 (65.1 to 135.58) | 68.23 (45.96 to 98.8) | -1.26 (-1.34 to -1.17) | 95.48 (65.31 to 135.66) | 76.74 (51.43 to 111.92) | -0.88 (-1.01 to -0.74) |
| Romania | 28362 (18787 to 42455) | 17446 (11520 to 26304) | -0.38 (-0.42 to -0.35) | 121.22 (80.3 to 181.46) | 90.69 (59.89 to 136.74) | -1.23 (-1.34 to -1.11) | 124.87 (82.67 to 186.63) | 107.1 (70.14 to 161.1) | -0.71 (-0.84 to -0.58) |
| Russian Federation | 172403 (116227 to 248708) | 129043 (86340 to 190486) | -0.25 (-0.3 to -0.2) | 114.16 (76.96 to 164.68) | 87.95 (58.85 to 129.83) | -0.82 (-1.14 to -0.5) | 116.99 (79.06 to 169.63) | 95.38 (63.5 to 141.15) | -0.68 (-0.91 to -0.45) |
| Rwanda | 3072 (2112 to 4404) | 4890 (3323 to 7129) | 0.59 (0.49 to 0.71) | 42.83 (29.44 to 61.4) | 38.54 (26.19 to 56.19) | -0.51 (-0.74 to -0.29) | 41.81 (28.51 to 60.03) | 37.16 (25.48 to 53.99) | -0.59 (-0.83 to -0.36) |
| Saint Kitts and Nevis | 21 (15 to 32) | 31 (21 to 45) | 0.43 (0.32 to 0.53) | 51.98 (35.64 to 77.18) | 51.5 (35.53 to 76.05) | -0.17 (-0.34 to 0) | 50.82 (34.93 to 75.6) | 54.4 (37.51 to 80.23) | 0.1 (-0.06 to 0.27) |
| Saint Lucia | 50 (36 to 70) | 63 (45 to 89) | 0.25 (0.15 to 0.36) | 36.39 (26.47 to 50.94) | 35.89 (25.56 to 50.82) | -0.02 (-0.2 to 0.17) | 33.36 (24.29 to 46.47) | 38.71 (27.7 to 54.78) | 0.54 (0.37 to 0.71) |
| Saint Vincent and the Grenadines | 41 (30 to 58) | 43 (31 to 61) | 0.04 (-0.04 to 0.11) | 37.67 (27.65 to 52.35) | 37.98 (27.3 to 54.01) | 0.02 (-0.22 to 0.26) | 34.56 (25.3 to 47.93) | 39.68 (28.45 to 56.22) | 0.48 (0.24 to 0.71) |
| Samoa | 49 (34 to 71) | 60 (40 to 94) | 0.23 (-0.01 to 0.44) | 29.88 (20.94 to 43.1) | 28.57 (18.74 to 44.49) | 0.41 (-1.03 to 1.88) | 28.42 (20.21 to 40.37) | 27.49 (18.25 to 41.9) | 0.51 (-0.96 to 1.99) |
| San Marino | 18 (12 to 28) | 25 (17 to 40) | 0.43 (0.34 to 0.52) | 75.24 (50.06 to 117.1) | 76.68 (51.59 to 119.53) | 0.08 (0.02 to 0.14) | 80.42 (52.54 to 126.05) | 85.88 (55.41 to 137.19) | 0.23 (0.17 to 0.3) |
| Sao Tome and Principe | 47 (32 to 67) | 81 (56 to 115) | 0.71 (0.6 to 0.82) | 38.75 (26.59 to 55.1) | 39.25 (27.28 to 56.19) | 0 (-0.06 to 0.06) | 35.49 (24.78 to 50.18) | 38.82 (27.2 to 55.74) | 0.29 (0.23 to 0.36) |
| Saudi Arabia | 13769 (8889 to 20865) | 38845 (24085 to 61395) | 1.82 (1.44 to 2.15) | 85.81 (55.4 to 130.04) | 108.71 (67.4 to 171.82) | 1.01 (0.85 to 1.17) | 78.45 (51.3 to 118.9) | 94.93 (59.07 to 150.56) | 0.75 (0.61 to 0.89) |
| Senegal | 2451 (1690 to 3477) | 4710 (3253 to 6733) | 0.92 (0.83 to 1.01) | 32.17 (22.18 to 45.63) | 31.12 (21.49 to 44.49) | -0.12 (-0.23 to -0.01) | 30.44 (20.97 to 43.13) | 30.69 (21.17 to 43.48) | 0.01 (-0.08 to 0.1) |
| Serbia | 8412 (5706 to 12213) | 7614 (5019 to 11516) | -0.09 (-0.18 to -0.02) | 89.51 (60.72 to 129.96) | 87.04 (57.38 to 131.66) | -0.12 (-0.22 to -0.01) | 94.64 (64.01 to 138.13) | 101.94 (66.05 to 153.56) | 0.28 (0.16 to 0.39) |
| Seychelles | 27 (19 to 40) | 36 (25 to 53) | 0.33 (0.24 to 0.42) | 37.6 (25.86 to 54.14) | 35.63 (24.5 to 51.95) | -0.35 (-0.52 to -0.18) | 36.82 (25.4 to 52.96) | 36.44 (24.97 to 52.77) | -0.21 (-0.39 to -0.02) |
| Sierra Leone | 1201 (832 to 1703) | 2550 (1750 to 3637) | 1.12 (0.99 to 1.26) | 32.88 (22.79 to 46.64) | 30.77 (21.13 to 43.9) | 0.05 (-0.44 to 0.54) | 30.8 (21.09 to 43.42) | 30.58 (21.23 to 43.19) | 0.3 (-0.2 to 0.81) |
| Singapore | 2225 (1510 to 3344) | 3175 (2120 to 4791) | 0.43 (0.36 to 0.51) | 73 (49.54 to 109.73) | 56.02 (37.41 to 84.54) | -1.04 (-1.14 to -0.95) | 68.83 (46.6 to 103.65) | 64.48 (42.56 to 97.76) | -0.38 (-0.47 to -0.29) |
| Slovakia | 6350 (4210 to 9485) | 5748 (3769 to 8610) | -0.09 (-0.15 to -0.05) | 120.2 (79.7 to 179.54) | 105.71 (69.32 to 158.35) | -0.52 (-0.57 to -0.47) | 121.15 (80.52 to 180.92) | 118.52 (76.99 to 179.21) | -0.14 (-0.23 to -0.05) |
| Slovenia | 2604 (1732 to 3890) | 2309 (1522 to 3466) | -0.11 (-0.18 to -0.05) | 132.12 (87.87 to 197.34) | 111.32 (73.4 to 167.11) | -0.23 (-0.39 to -0.07) | 134.66 (89.51 to 200.21) | 128.27 (82.59 to 193.28) | 0.27 (0.09 to 0.44) |
| Solomon Islands | 113 (73 to 179) | 252 (154 to 417) | 1.23 (1.04 to 1.4) | 33.15 (21.33 to 52.58) | 38.42 (23.53 to 63.57) | 0.55 (0.22 to 0.87) | 32.83 (21.22 to 51.75) | 37.6 (23.25 to 62.21) | 0.53 (0.2 to 0.87) |
| Somalia | 2808 (1946 to 4007) | 8283 (5660 to 11923) | 1.95 (1.82 to 2.1) | 39.29 (27.23 to 56.07) | 40.72 (27.82 to 58.61) | 0.28 (-0.04 to 0.61) | 38.84 (27.06 to 56.1) | 40.03 (27.6 to 57.72) | 0.27 (-0.07 to 0.62) |
| South Africa | 15486 (10622 to 22364) | 18687 (12915 to 26206) | 0.21 (0.15 to 0.26) | 42.05 (28.84 to 60.72) | 33.62 (23.23 to 47.14) | -0.64 (-0.78 to -0.51) | 40.21 (27.75 to 57.9) | 33.36 (23.03 to 46.65) | -0.55 (-0.68 to -0.41) |
| South Sudan | 2456 (1695 to 3503) | 3767 (2587 to 5397) | 0.53 (0.47 to 0.61) | 41.92 (28.93 to 59.78) | 40.58 (27.87 to 58.14) | 0.38 (0.01 to 0.75) | 40.86 (28.39 to 58.01) | 39.31 (27.2 to 56.36) | 0.37 (0 to 0.75) |
| Spain | 24438 (16218 to 36304) | 25440 (15982 to 40852) | 0.04 (-0.08 to 0.16) | 63.01 (41.82 to 93.61) | 55.28 (34.73 to 88.77) | -0.65 (-0.71 to -0.59) | 66.57 (44.03 to 100.67) | 69.04 (41.76 to 113.56) | -0.04 (-0.1 to 0.02) |
| Sri Lanka | 6286 (4449 to 8902) | 7399 (4970 to 11008) | 0.18 (0.05 to 0.3) | 36.5 (25.84 to 51.69) | 33.85 (22.74 to 50.37) | -0.54 (-1.79 to 0.73) | 35.1 (24.96 to 49.71) | 34.53 (23.27 to 51.3) | -0.3 (-1.56 to 0.98) |
| Sudan | 9586 (6375 to 14039) | 19272 (13314 to 27924) | 1.01 (0.82 to 1.22) | 47.46 (31.56 to 69.51) | 47.23 (32.63 to 68.43) | -0.11 (-0.27 to 0.05) | 43.66 (29.46 to 64.06) | 43.59 (30.39 to 62.47) | -0.09 (-0.25 to 0.08) |
| Suriname | 132 (97 to 180) | 206 (149 to 286) | 0.56 (0.47 to 0.66) | 34.12 (25.1 to 46.54) | 35.79 (25.95 to 49.71) | 0.2 (0.16 to 0.23) | 31.68 (23.36 to 43.26) | 36.7 (26.6 to 50.86) | 0.53 (0.5 to 0.57) |
| Sweden | 7294 (4965 to 11116) | 8835 (5952 to 13512) | 0.21 (0.15 to 0.28) | 84.93 (57.81 to 129.42) | 86.43 (58.22 to 132.18) | 0 (-0.08 to 0.08) | 88.76 (59 to 137.71) | 89.61 (58.85 to 141.66) | -0.02 (-0.14 to 0.1) |
| Switzerland | 6626 (4263 to 10317) | 6766 (4247 to 10841) | 0.02 (-0.05 to 0.09) | 96.51 (62.09 to 150.28) | 77.1 (48.4 to 123.54) | -1.21 (-1.4 to -1.02) | 102.42 (64.76 to 161.9) | 90.17 (54.23 to 149.71) | -0.88 (-1.12 to -0.65) |
| Syrian Arab Republic | 5012 (3386 to 7479) | 6326 (4096 to 10112) | 0.26 (0.13 to 0.39) | 38.87 (26.26 to 58) | 43.65 (28.27 to 69.78) | 0.42 (0.2 to 0.64) | 33.44 (22.84 to 49.34) | 43.24 (28.13 to 67.76) | 0.9 (0.58 to 1.23) |
| Taiwan (Province of China) | 5948 (3885 to 9332) | 4398 (2996 to 6545) | -0.26 (-0.33 to -0.18) | 29.16 (19.05 to 45.75) | 18.62 (12.68 to 27.71) | -2.06 (-2.48 to -1.65) | 28.12 (18.47 to 43.67) | 18.6 (12.48 to 27.96) | -1.93 (-2.36 to -1.51) |
| Tajikistan | 3672 (2432 to 5469) | 5312 (3567 to 7849) | 0.45 (0.36 to 0.54) | 68.3 (45.25 to 101.74) | 55.96 (37.57 to 82.69) | -0.93 (-1.18 to -0.67) | 61.93 (41.75 to 90.95) | 51.32 (34.56 to 75.28) | -0.88 (-1.18 to -0.58) |
| Thailand | 20314 (14211 to 28741) | 20941 (14268 to 30146) | 0.03 (-0.07 to 0.14) | 35.72 (24.99 to 50.53) | 29.87 (20.35 to 43) | -0.81 (-1.04 to -0.58) | 33.84 (23.72 to 47.73) | 31.49 (21.44 to 45.59) | -0.45 (-0.68 to -0.22) |
| Timor-Leste | 205 (146 to 290) | 356 (246 to 519) | 0.73 (0.59 to 0.89) | 26.22 (18.65 to 37.01) | 26.67 (18.4 to 38.86) | 0 (-0.15 to 0.15) | 24.41 (17.38 to 34.19) | 25.23 (17.46 to 36.31) | 0.06 (-0.07 to 0.18) |
| Togo | 1282 (882 to 1836) | 2693 (1852 to 3864) | 1.1 (1.01 to 1.2) | 35 (24.08 to 50.12) | 34 (23.38 to 48.78) | -0.07 (-0.2 to 0.07) | 32.92 (22.7 to 46.52) | 33.23 (23.05 to 47.07) | 0.05 (-0.07 to 0.18) |
| Tokelau | 0 (0 to 1) | 0 (0 to 1) | -0.08 (-0.16 to 0) | 29.23 (19.96 to 43.09) | 32 (21.8 to 48.91) | 0.29 (0.2 to 0.39) | 29.26 (20.08 to 43.44) | 32.54 (22.17 to 49.55) | 0.33 (0.23 to 0.44) |
| Tonga | 24 (17 to 35) | 26 (17 to 41) | 0.09 (-0.03 to 0.2) | 24.55 (17.17 to 35.87) | 25.21 (16.37 to 40.45) | 0.21 (-0.17 to 0.6) | 23.42 (16.65 to 33.93) | 24.36 (15.99 to 38.24) | 0.26 (-0.14 to 0.66) |
| Trinidad and Tobago | 410 (296 to 568) | 442 (317 to 618) | 0.08 (0.02 to 0.14) | 34.07 (24.59 to 47.19) | 31.88 (22.85 to 44.54) | -0.13 (-0.2 to -0.05) | 32 (23.16 to 44.06) | 35.22 (25.24 to 49.78) | 0.44 (0.38 to 0.5) |
| Tunisia | 3460 (2369 to 4960) | 4399 (2973 to 6338) | 0.27 (0.18 to 0.37) | 41 (28.08 to 58.77) | 38.01 (25.7 to 54.77) | -0.19 (-0.29 to -0.09) | 37.89 (26.17 to 54.35) | 39.28 (26.41 to 56.93) | 0.2 (0.1 to 0.29) |
| Turkey | 21916 (15253 to 31456) | 31048 (20694 to 46541) | 0.42 (0.25 to 0.59) | 36.66 (25.52 to 52.63) | 38.16 (25.43 to 57.2) | -0.05 (-0.85 to 0.75) | 33.67 (23.49 to 48.06) | 39.04 (25.87 to 59) | 0.31 (-0.51 to 1.13) |
| Turkmenistan | 2188 (1487 to 3212) | 2925 (1992 to 4353) | 0.34 (0.26 to 0.42) | 59.04 (40.14 to 86.68) | 57.54 (39.19 to 85.64) | -0.05 (-0.12 to 0.03) | 52.31 (35.66 to 76.79) | 55.79 (37.76 to 82.62) | 0.3 (0.18 to 0.42) |
| Tuvalu | 3 (2 to 4) | 4 (3 to 6) | 0.42 (0.3 to 0.54) | 28.77 (19.69 to 42.06) | 32.24 (21.97 to 48.49) | 0.35 (0.23 to 0.46) | 28.56 (19.63 to 41.96) | 31.87 (21.85 to 47.75) | 0.31 (0.21 to 0.41) |
| Uganda | 6519 (4437 to 9260) | 15951 (10915 to 22967) | 1.45 (1.34 to 1.55) | 37.65 (25.62 to 53.48) | 38.79 (26.55 to 55.86) | 0.11 (-0.02 to 0.24) | 34.6 (23.82 to 49.52) | 35.46 (24.62 to 51.77) | 0.06 (-0.05 to 0.17) |
| Ukraine | 59303 (38921 to 91158) | 38948 (25927 to 58929) | -0.34 (-0.37 to -0.31) | 112.61 (73.91 to 173.1) | 88.43 (58.87 to 133.8) | -1.13 (-1.29 to -0.97) | 118.85 (78.05 to 182.85) | 100 (66.46 to 150.3) | -0.93 (-1.14 to -0.73) |
| United Arab Emirates | 931 (634 to 1341) | 4156 (2789 to 6176) | 3.46 (3.15 to 3.81) | 49.74 (33.88 to 71.65) | 44.97 (30.17 to 66.82) | -0.25 (-0.44 to -0.06) | 45.09 (30.54 to 64.5) | 44.2 (29.68 to 64.91) | -0.2 (-0.26 to -0.14) |
| United Kingdom | 36084 (24094 to 55013) | 41265 (26548 to 64632) | 0.14 (0.05 to 0.21) | 62.78 (41.92 to 95.72) | 61.39 (39.49 to 96.15) | -0.1 (-0.19 to -0.01) | 69.99 (46.26 to 109.83) | 69.82 (44.16 to 114.08) | -0.05 (-0.16 to 0.06) |
| United Republic of Tanzania | 10143 (6995 to 14491) | 21750 (14909 to 31464) | 1.14 (1.06 to 1.24) | 39.16 (27.01 to 55.95) | 38.34 (26.28 to 55.46) | -0.02 (-0.15 to 0.11) | 36.51 (25.37 to 52.82) | 35.9 (24.81 to 51.97) | -0.02 (-0.14 to 0.1) |
| United States of America | 231454 (156889 to 346697) | 278136 (193522 to 404513) | 0.2 (0.11 to 0.29) | 91.27 (61.86 to 136.71) | 84.8 (59 to 123.34) | -0.61 (-0.94 to -0.27) | 91.65 (61.63 to 139.12) | 83.05 (57.01 to 123.4) | -0.88 (-1.27 to -0.48) |
| United States Virgin Islands | 50 (35 to 72) | 46 (32 to 67) | -0.08 (-0.16 to -0.02) | 47.39 (33.01 to 68.05) | 44.24 (31.02 to 64.14) | -0.33 (-0.48 to -0.18) | 47.12 (32.97 to 67.14) | 49.36 (34.27 to 71.33) | 0.06 (-0.09 to 0.21) |
| Uruguay | 2607 (1723 to 4028) | 2433 (1628 to 3654) | -0.07 (-0.15 to 0.02) | 83.03 (54.89 to 128.29) | 70.82 (47.38 to 106.35) | -0.74 (-0.83 to -0.64) | 85.43 (56.43 to 131.49) | 76.63 (50.9 to 115.51) | -0.56 (-0.67 to -0.46) |
| Uzbekistan | 13986 (9455 to 20635) | 20207 (13693 to 29536) | 0.44 (0.37 to 0.52) | 66.77 (45.14 to 98.51) | 60 (40.66 to 87.7) | -0.52 (-0.62 to -0.43) | 59.54 (40.94 to 86.92) | 56.55 (38.27 to 82.39) | -0.33 (-0.4 to -0.25) |
| Vanuatu | 36 (25 to 53) | 76 (50 to 116) | 1.12 (0.96 to 1.27) | 23.66 (16.27 to 34.84) | 25.79 (17.03 to 39.38) | -0.4 (-1.7 to 0.92) | 22.27 (15.52 to 32.4) | 24.4 (16.37 to 36.79) | -0.41 (-1.76 to 0.96) |
| Venezuela (Bolivarian Republic of) | 10920 (7372 to 16150) | 13521 (9145 to 20198) | 0.24 (0.17 to 0.31) | 57.99 (39.15 to 85.77) | 48.17 (32.58 to 71.96) | -0.92 (-1.9 to 0.07) | 51.41 (34.88 to 75.75) | 50.36 (33.92 to 75.96) | -0.38 (-1.39 to 0.63) |
| Viet Nam | 20476 (14434 to 28912) | 31436 (21547 to 45666) | 0.54 (0.39 to 0.69) | 30.14 (21.25 to 42.55) | 32.62 (22.36 to 47.38) | 0.17 (0.04 to 0.31) | 29.23 (20.71 to 41.53) | 32.99 (22.71 to 48.06) | 0.28 (0.13 to 0.43) |
| Yemen | 5336 (3712 to 7691) | 11916 (8107 to 17332) | 1.23 (1.06 to 1.4) | 38.87 (27.04 to 56.02) | 37.82 (25.74 to 55.02) | -0.1 (-0.2 to 0.01) | 35.4 (24.51 to 49.92) | 34.33 (23.46 to 49.73) | -0.09 (-0.21 to 0.02) |
| Zambia | 2865 (1957 to 4062) | 6677 (4557 to 9685) | 1.33 (1.2 to 1.48) | 36.07 (24.64 to 51.13) | 36.61 (24.98 to 53.1) | 0.01 (-0.09 to 0.11) | 34.5 (23.91 to 49.75) | 35.13 (24.72 to 50.24) | 0 (-0.09 to 0.09) |
| Zimbabwe | 3417 (2345 to 4791) | 5656 (3963 to 7871) | 0.66 (0.45 to 1.11) | 33.06 (22.69 to 46.34) | 37.68 (26.4 to 52.44) | -0.02 (-0.23 to 0.18) | 30.58 (21.4 to 42.78) | 36.29 (25.59 to 50.54) | 0.05 (-0.15 to 0.26) |

CR, crude rate; ASR, age-standardized rate; EAPC, estimated annual percentage change; UI, uncertainty interval; CI, confidence interval.

Table S4. YLDs of knee dislocation in 1990 and 2019 for both sexes in 204 countries and territories, with EAPC from 1990 and 2019.

| Location | **Number** | | | **CR, per 100k** | | | **ASR, per 100k** | | |
| --- | --- | --- | --- | --- | --- | --- | --- | --- | --- |
|  | **Number in 1990 (95% UI)** | **Number in 2019 (95% UI)** | **Number change rate (95% UI)** | **CR in 1990 (95% UI)** | **CR in 2019 (95% UI)** | **EAPC of CR, % per year (95% CI)** | **ASR in 1990 (95% UI)** | **ASR in 2019 (95% UI)** | **EAPC of ASR, % per year (95% CI)** |
| Afghanistan | 79 (48 to 126) | 260 (156 to 412) | 2.29 (2.03 to 2.59) | 0.69 (0.42 to 1.1) | 0.68 (0.41 to 1.08) | -0.64 (-1.38 to 0.11) | 0.78 (0.48 to 1.18) | 0.82 (0.52 to 1.23) | -0.39 (-1.08 to 0.3) |
| Albania | 76 (43 to 124) | 65 (40 to 101) | -0.15 (-0.26 to -0.01) | 2.3 (1.31 to 3.74) | 2.38 (1.45 to 3.71) | -0.12 (-0.24 to 0) | 2.38 (1.4 to 3.8) | 2.26 (1.32 to 3.6) | -0.44 (-0.57 to -0.32) |
| Algeria | 219 (128 to 345) | 370 (222 to 574) | 0.69 (0.57 to 0.84) | 0.87 (0.51 to 1.36) | 0.88 (0.53 to 1.37) | -0.13 (-0.34 to 0.09) | 0.98 (0.59 to 1.49) | 0.91 (0.56 to 1.41) | -0.45 (-0.63 to -0.26) |
| American Samoa | 0 (0 to 1) | 1 (0 to 1) | 0.44 (0.24 to 0.69) | 0.89 (0.56 to 1.37) | 1.12 (0.71 to 1.68) | 0.99 (0.34 to 1.64) | 1.13 (0.73 to 1.71) | 1.22 (0.79 to 1.78) | 0.44 (-0.13 to 1.01) |
| Andorra | 1 (1 to 1) | 2 (1 to 3) | 0.95 (0.71 to 1.31) | 1.64 (0.96 to 2.6) | 2.08 (1.29 to 3.17) | 0.84 (0.78 to 0.9) | 1.7 (1 to 2.71) | 1.79 (1.03 to 2.87) | 0.12 (0.07 to 0.17) |
| Angola | 53 (32 to 83) | 148 (90 to 230) | 1.78 (1.64 to 1.95) | 0.52 (0.31 to 0.8) | 0.49 (0.3 to 0.76) | -0.21 (-0.28 to -0.14) | 0.65 (0.42 to 0.97) | 0.63 (0.4 to 0.94) | -0.12 (-0.17 to -0.08) |
| Antigua and Barbuda | 1 (0 to 1) | 1 (1 to 1) | 0.68 (0.55 to 0.83) | 0.89 (0.54 to 1.37) | 1.03 (0.64 to 1.6) | 0.32 (0.11 to 0.53) | 0.9 (0.55 to 1.39) | 1 (0.61 to 1.56) | 0.18 (-0.07 to 0.42) |
| Argentina | 488 (291 to 781) | 682 (417 to 1063) | 0.4 (0.28 to 0.54) | 1.47 (0.88 to 2.36) | 1.51 (0.92 to 2.36) | -0.02 (-0.08 to 0.05) | 1.49 (0.89 to 2.38) | 1.48 (0.89 to 2.34) | -0.13 (-0.19 to -0.07) |
| Armenia | 80 (47 to 123) | 47 (30 to 71) | -0.41 (-0.52 to -0.29) | 2.34 (1.39 to 3.61) | 1.56 (0.99 to 2.34) | -1.52 (-1.69 to -1.34) | 2.46 (1.46 to 3.78) | 1.47 (0.91 to 2.22) | -1.94 (-2.15 to -1.72) |
| Australia | 442 (265 to 700) | 719 (440 to 1119) | 0.63 (0.46 to 0.85) | 2.62 (1.57 to 4.15) | 2.93 (1.79 to 4.56) | 0.39 (0.32 to 0.46) | 2.61 (1.54 to 4.2) | 2.71 (1.59 to 4.43) | 0.12 (0.06 to 0.19) |
| Austria | 165 (100 to 254) | 189 (117 to 285) | 0.14 (0.01 to 0.31) | 2.12 (1.28 to 3.27) | 2.12 (1.31 to 3.19) | -0.07 (-0.12 to -0.03) | 1.91 (1.12 to 3.05) | 1.75 (1.03 to 2.8) | -0.4 (-0.44 to -0.36) |
| Azerbaijan | 89 (53 to 142) | 124 (74 to 193) | 0.39 (0.27 to 0.54) | 1.22 (0.72 to 1.94) | 1.2 (0.72 to 1.88) | 0 (-0.2 to 0.19) | 1.25 (0.76 to 1.97) | 1.2 (0.72 to 1.88) | -0.11 (-0.32 to 0.1) |
| Bahamas | 2 (1 to 3) | 3 (2 to 5) | 0.78 (0.65 to 0.95) | 0.73 (0.45 to 1.1) | 0.89 (0.56 to 1.33) | 0.69 (0.57 to 0.81) | 0.79 (0.5 to 1.18) | 0.88 (0.56 to 1.33) | 0.38 (0.26 to 0.49) |
| Bahrain | 4 (2 to 6) | 13 (8 to 21) | 2.41 (2.19 to 2.65) | 0.75 (0.45 to 1.19) | 0.91 (0.55 to 1.42) | 0.71 (0.6 to 0.82) | 0.82 (0.5 to 1.25) | 0.91 (0.55 to 1.44) | 0.39 (0.28 to 0.49) |
| Bangladesh | 673 (417 to 1038) | 1317 (801 to 1975) | 0.96 (0.78 to 1.17) | 0.62 (0.38 to 0.95) | 0.83 (0.5 to 1.24) | -0.38 (-1.65 to 0.9) | 0.75 (0.48 to 1.11) | 0.86 (0.52 to 1.27) | -0.92 (-1.99 to 0.15) |
| Barbados | 2 (1 to 3) | 3 (2 to 4) | 0.37 (0.25 to 0.51) | 0.76 (0.48 to 1.17) | 0.89 (0.56 to 1.35) | 0.48 (0.42 to 0.55) | 0.74 (0.46 to 1.14) | 0.83 (0.51 to 1.28) | 0.33 (0.27 to 0.38) |
| Belarus | 263 (160 to 401) | 251 (156 to 387) | -0.04 (-0.16 to 0.11) | 2.51 (1.53 to 3.83) | 2.64 (1.64 to 4.08) | 0.44 (0.17 to 0.72) | 2.39 (1.44 to 3.73) | 2.31 (1.38 to 3.63) | 0.1 (-0.12 to 0.32) |
| Belgium | 200 (121 to 310) | 295 (180 to 452) | 0.48 (0.32 to 0.67) | 2.01 (1.21 to 3.1) | 2.59 (1.58 to 3.96) | 1.32 (1 to 1.65) | 1.84 (1.06 to 2.96) | 2.06 (1.17 to 3.34) | 1.02 (0.6 to 1.43) |
| Belize | 1 (1 to 2) | 4 (2 to 6) | 1.8 (1.62 to 2.02) | 0.73 (0.44 to 1.14) | 0.93 (0.57 to 1.45) | 0.69 (0.47 to 0.91) | 0.81 (0.5 to 1.23) | 0.98 (0.61 to 1.51) | 0.52 (0.3 to 0.74) |
| Benin | 38 (24 to 60) | 98 (62 to 153) | 1.58 (1.39 to 1.8) | 0.79 (0.5 to 1.23) | 0.78 (0.49 to 1.2) | -0.08 (-0.16 to 0) | 1.12 (0.71 to 1.66) | 1.13 (0.73 to 1.67) | 0.03 (-0.01 to 0.07) |
| Bermuda | 1 (0 to 1) | 1 (1 to 2) | 0.51 (0.28 to 0.83) | 1.29 (0.8 to 1.94) | 1.81 (1.16 to 2.62) | 1.16 (1.02 to 1.29) | 1.26 (0.79 to 1.92) | 1.44 (0.9 to 2.17) | 0.43 (0.33 to 0.52) |
| Bhutan | 4 (3 to 7) | 8 (5 to 12) | 0.76 (0.58 to 0.96) | 0.72 (0.44 to 1.12) | 1.03 (0.64 to 1.57) | 1.05 (0.73 to 1.37) | 0.9 (0.55 to 1.35) | 1.1 (0.69 to 1.67) | 0.59 (0.32 to 0.85) |
| Bolivia (Plurinational State of) | 50 (30 to 78) | 96 (59 to 152) | 0.92 (0.78 to 1.07) | 0.78 (0.47 to 1.21) | 0.8 (0.49 to 1.26) | -0.07 (-0.13 to 0) | 0.87 (0.55 to 1.33) | 0.85 (0.52 to 1.34) | -0.25 (-0.32 to -0.18) |
| Bosnia and Herzegovina | 96 (57 to 153) | 79 (49 to 121) | -0.17 (-0.28 to -0.03) | 2.12 (1.26 to 3.37) | 2.4 (1.47 to 3.68) | 0.56 (0.46 to 0.67) | 2.12 (1.28 to 3.36) | 2.27 (1.35 to 3.57) | 0.36 (0.25 to 0.46) |
| Botswana | 11 (7 to 17) | 22 (14 to 34) | 1.06 (0.86 to 1.29) | 0.83 (0.52 to 1.28) | 0.95 (0.59 to 1.43) | 0.36 (0.31 to 0.42) | 1.04 (0.66 to 1.55) | 1.09 (0.7 to 1.6) | 0.05 (-0.01 to 0.11) |
| Brazil | 2758 (1724 to 4291) | 4200 (2702 to 6342) | 0.52 (0.41 to 0.66) | 1.85 (1.16 to 2.88) | 1.94 (1.25 to 2.93) | 0.18 (0.11 to 0.25) | 2.16 (1.37 to 3.25) | 1.85 (1.19 to 2.78) | -0.49 (-0.55 to -0.42) |
| Brunei Darussalam | 4 (2 to 6) | 6 (4 to 10) | 0.66 (0.54 to 0.82) | 1.44 (0.83 to 2.32) | 1.41 (0.83 to 2.24) | -0.11 (-0.22 to -0.01) | 1.61 (0.96 to 2.52) | 1.47 (0.89 to 2.31) | -0.37 (-0.46 to -0.29) |
| Bulgaria | 242 (149 to 379) | 184 (113 to 282) | -0.24 (-0.33 to -0.12) | 2.78 (1.72 to 4.37) | 2.65 (1.63 to 4.07) | -0.16 (-0.21 to -0.11) | 2.66 (1.61 to 4.23) | 2.43 (1.41 to 3.87) | -0.31 (-0.38 to -0.24) |
| Burkina Faso | 72 (45 to 110) | 190 (120 to 286) | 1.62 (1.36 to 1.94) | 0.76 (0.47 to 1.15) | 0.84 (0.53 to 1.26) | 0.24 (0.06 to 0.42) | 1.08 (0.7 to 1.57) | 1.22 (0.8 to 1.8) | 0.37 (0.22 to 0.53) |
| Burundi | 36 (22 to 55) | 76 (46 to 117) | 1.11 (0.97 to 1.26) | 0.64 (0.39 to 0.99) | 0.63 (0.39 to 0.98) | -0.29 (-0.81 to 0.24) | 0.82 (0.52 to 1.24) | 0.79 (0.49 to 1.18) | -0.34 (-0.76 to 0.09) |
| Cabo Verde | 3 (2 to 5) | 6 (4 to 9) | 0.89 (0.67 to 1.17) | 0.88 (0.56 to 1.34) | 1.04 (0.67 to 1.56) | 0.66 (0.53 to 0.8) | 1.03 (0.66 to 1.52) | 1.14 (0.74 to 1.71) | 0.41 (0.35 to 0.46) |
| Cambodia | 59 (34 to 94) | 130 (77 to 204) | 1.19 (1.03 to 1.38) | 0.57 (0.33 to 0.91) | 0.78 (0.46 to 1.23) | 0.97 (0.83 to 1.11) | 0.71 (0.43 to 1.09) | 0.87 (0.53 to 1.33) | 0.55 (0.43 to 0.67) |
| Cameroon | 88 (56 to 133) | 254 (160 to 382) | 1.89 (1.6 to 2.2) | 0.85 (0.54 to 1.28) | 0.87 (0.55 to 1.31) | 0.13 (0.04 to 0.23) | 1.22 (0.8 to 1.76) | 1.26 (0.82 to 1.86) | 0.16 (0.11 to 0.22) |
| Canada | 419 (257 to 641) | 703 (453 to 1036) | 0.68 (0.47 to 0.95) | 1.54 (0.94 to 2.35) | 1.93 (1.24 to 2.84) | 0.84 (0.79 to 0.89) | 1.44 (0.87 to 2.23) | 1.43 (0.89 to 2.2) | -0.01 (-0.05 to 0.02) |
| Central African Republic | 13 (8 to 20) | 26 (16 to 39) | 1.02 (0.92 to 1.13) | 0.47 (0.29 to 0.72) | 0.49 (0.3 to 0.74) | 0.5 (0.22 to 0.78) | 0.59 (0.37 to 0.88) | 0.61 (0.4 to 0.88) | 0.4 (0.2 to 0.61) |
| Chad | 45 (28 to 69) | 121 (76 to 190) | 1.69 (1.45 to 1.93) | 0.75 (0.47 to 1.15) | 0.74 (0.46 to 1.16) | -0.02 (-0.07 to 0.03) | 1.01 (0.66 to 1.49) | 1.12 (0.72 to 1.64) | 0.41 (0.37 to 0.45) |
| Chile | 186 (110 to 293) | 296 (178 to 465) | 0.59 (0.45 to 0.79) | 1.4 (0.83 to 2.2) | 1.63 (0.98 to 2.55) | 0.42 (0.22 to 0.62) | 1.46 (0.88 to 2.28) | 1.56 (0.92 to 2.47) | 0.2 (0 to 0.41) |
| China | 11382 (7169 to 17280) | 22806 (15051 to 33101) | 1 (0.81 to 1.2) | 0.96 (0.61 to 1.46) | 1.6 (1.06 to 2.33) | 1.22 (0.78 to 1.67) | 1.12 (0.72 to 1.67) | 1.33 (0.87 to 1.97) | 0.03 (-0.38 to 0.43) |
| Colombia | 398 (238 to 618) | 487 (301 to 733) | 0.22 (0.1 to 0.37) | 1.22 (0.73 to 1.9) | 1.02 (0.63 to 1.53) | -0.8 (-0.91 to -0.69) | 1.33 (0.81 to 2.03) | 1.01 (0.62 to 1.54) | -1.12 (-1.23 to -1.01) |
| Comoros | 5 (3 to 8) | 9 (5 to 13) | 0.74 (0.54 to 0.97) | 1.07 (0.67 to 1.62) | 1.22 (0.76 to 1.84) | 0.32 (0.18 to 0.45) | 1.42 (0.89 to 2.09) | 1.4 (0.88 to 2.07) | -0.17 (-0.29 to -0.06) |
| Congo | 12 (7 to 19) | 26 (16 to 40) | 1.15 (1.04 to 1.29) | 0.49 (0.3 to 0.77) | 0.49 (0.3 to 0.76) | 0.01 (-0.11 to 0.13) | 0.62 (0.39 to 0.94) | 0.59 (0.37 to 0.88) | -0.18 (-0.28 to -0.08) |
| Cook Islands | 0 (0 to 0) | 0 (0 to 0) | 0.33 (0.12 to 0.6) | 1 (0.64 to 1.51) | 1.4 (0.87 to 2.08) | 0.95 (0.31 to 1.59) | 1.17 (0.76 to 1.72) | 1.25 (0.78 to 1.86) | -0.06 (-0.71 to 0.6) |
| Costa Rica | 30 (18 to 49) | 51 (31 to 79) | 0.67 (0.52 to 0.87) | 1 (0.58 to 1.6) | 1.08 (0.66 to 1.67) | 0.27 (0.15 to 0.39) | 1.08 (0.65 to 1.66) | 1.08 (0.64 to 1.69) | 0.02 (-0.08 to 0.12) |
| Côte d'Ivoire | 94 (58 to 145) | 212 (131 to 323) | 1.27 (1.06 to 1.53) | 0.77 (0.47 to 1.19) | 0.81 (0.5 to 1.23) | 0.16 (0.06 to 0.26) | 1.16 (0.75 to 1.71) | 1.16 (0.73 to 1.72) | -0.05 (-0.17 to 0.06) |
| Croatia | 139 (85 to 219) | 134 (84 to 200) | -0.03 (-0.17 to 0.13) | 2.84 (1.74 to 4.47) | 3.16 (1.99 to 4.72) | 0.62 (0.54 to 0.7) | 2.69 (1.64 to 4.26) | 2.57 (1.53 to 4.09) | 0.08 (-0.01 to 0.17) |
| Cuba | 115 (72 to 174) | 171 (109 to 249) | 0.49 (0.32 to 0.69) | 1.06 (0.66 to 1.61) | 1.51 (0.96 to 2.2) | 1.25 (1.17 to 1.33) | 1.07 (0.67 to 1.61) | 1.25 (0.78 to 1.9) | 0.57 (0.52 to 0.63) |
| Cyprus | 12 (7 to 18) | 23 (14 to 36) | 0.92 (0.74 to 1.16) | 1.52 (0.89 to 2.36) | 1.73 (1.04 to 2.73) | 0.45 (0.38 to 0.51) | 1.55 (0.91 to 2.43) | 1.67 (0.96 to 2.68) | 0.26 (0.21 to 0.32) |
| Czechia | 323 (196 to 490) | 313 (195 to 468) | -0.03 (-0.14 to 0.1) | 3.13 (1.91 to 4.76) | 2.94 (1.83 to 4.4) | -0.12 (-0.19 to -0.05) | 2.91 (1.77 to 4.52) | 2.58 (1.54 to 4.05) | -0.23 (-0.3 to -0.15) |
| Democratic People's Republic of Korea | 100 (60 to 154) | 130 (83 to 193) | 0.3 (0.17 to 0.44) | 0.48 (0.28 to 0.73) | 0.5 (0.31 to 0.73) | 0.26 (0.04 to 0.48) | 0.52 (0.31 to 0.78) | 0.45 (0.28 to 0.67) | -0.36 (-0.55 to -0.18) |
| Democratic Republic of the Congo | 189 (115 to 294) | 432 (260 to 672) | 1.28 (1.18 to 1.38) | 0.49 (0.3 to 0.76) | 0.49 (0.3 to 0.77) | -0.02 (-0.16 to 0.12) | 0.61 (0.38 to 0.92) | 0.61 (0.39 to 0.92) | -0.02 (-0.13 to 0.09) |
| Denmark | 104 (65 to 159) | 107 (66 to 165) | 0.03 (-0.09 to 0.17) | 2.02 (1.27 to 3.09) | 1.85 (1.13 to 2.85) | -0.55 (-0.66 to -0.43) | 1.74 (1.05 to 2.74) | 1.62 (0.93 to 2.63) | -0.38 (-0.48 to -0.28) |
| Djibouti | 4 (3 to 7) | 12 (8 to 19) | 1.87 (1.59 to 2.22) | 0.89 (0.56 to 1.37) | 1.03 (0.64 to 1.56) | 0.32 (0.1 to 0.53) | 1.29 (0.82 to 1.91) | 1.3 (0.82 to 1.9) | -0.1 (-0.26 to 0.07) |
| Dominica | 1 (0 to 1) | 1 (0 to 1) | 0.2 (0.06 to 0.44) | 0.74 (0.46 to 1.12) | 0.96 (0.61 to 1.42) | 1.14 (0.79 to 1.49) | 0.75 (0.47 to 1.12) | 0.9 (0.57 to 1.36) | 0.86 (0.54 to 1.19) |
| Dominican Republic | 50 (31 to 78) | 104 (64 to 160) | 1.06 (0.91 to 1.26) | 0.7 (0.43 to 1.09) | 0.96 (0.59 to 1.47) | 1.12 (0.95 to 1.3) | 0.77 (0.48 to 1.15) | 0.97 (0.61 to 1.49) | 0.85 (0.69 to 1.02) |
| Ecuador | 87 (53 to 136) | 174 (107 to 270) | 0.98 (0.84 to 1.13) | 0.87 (0.53 to 1.36) | 0.99 (0.61 to 1.54) | 0.36 (0.23 to 0.5) | 0.97 (0.61 to 1.46) | 1.01 (0.63 to 1.57) | 0.1 (-0.03 to 0.22) |
| Egypt | 349 (212 to 549) | 695 (413 to 1102) | 0.99 (0.89 to 1.1) | 0.63 (0.38 to 0.99) | 0.7 (0.42 to 1.11) | 0.33 (0.19 to 0.48) | 0.69 (0.43 to 1.06) | 0.75 (0.45 to 1.16) | 0.24 (0.11 to 0.38) |
| El Salvador | 52 (30 to 82) | 67 (39 to 105) | 0.28 (0.18 to 0.42) | 0.99 (0.58 to 1.55) | 1.06 (0.63 to 1.67) | 0.19 (-0.03 to 0.41) | 1.06 (0.64 to 1.6) | 1.07 (0.63 to 1.68) | -0.06 (-0.27 to 0.15) |
| Equatorial Guinea | 2 (1 to 3) | 7 (4 to 11) | 2.2 (1.98 to 2.43) | 0.5 (0.3 to 0.77) | 0.48 (0.29 to 0.74) | -0.04 (-0.18 to 0.09) | 0.61 (0.38 to 0.9) | 0.6 (0.38 to 0.91) | 0.02 (-0.07 to 0.11) |
| Eritrea | 26 (16 to 41) | 64 (40 to 97) | 1.45 (1.25 to 1.69) | 0.87 (0.54 to 1.36) | 0.95 (0.59 to 1.45) | 0.27 (0.18 to 0.35) | 1.25 (0.79 to 1.84) | 1.29 (0.83 to 1.95) | 0.06 (0.01 to 0.1) |
| Estonia | 47 (29 to 73) | 31 (19 to 46) | -0.35 (-0.43 to -0.26) | 3.02 (1.87 to 4.67) | 2.33 (1.45 to 3.52) | -1.09 (-1.21 to -0.98) | 2.82 (1.72 to 4.43) | 2.02 (1.19 to 3.22) | -1.34 (-1.44 to -1.23) |
| Eswatini | 6 (4 to 9) | 10 (6 to 15) | 0.65 (0.52 to 0.81) | 0.74 (0.46 to 1.17) | 0.86 (0.55 to 1.32) | 0.59 (0.53 to 0.64) | 0.98 (0.62 to 1.46) | 1.04 (0.68 to 1.56) | 0.26 (0.17 to 0.36) |
| Ethiopia | 353 (215 to 547) | 653 (393 to 1031) | 0.85 (0.75 to 0.96) | 0.69 (0.42 to 1.06) | 0.61 (0.37 to 0.96) | -0.49 (-0.72 to -0.26) | 0.9 (0.57 to 1.33) | 0.75 (0.47 to 1.12) | -0.73 (-0.91 to -0.55) |
| Fiji | 4 (2 to 6) | 6 (3 to 9) | 0.43 (0.36 to 0.51) | 0.51 (0.3 to 0.8) | 0.6 (0.36 to 0.94) | 0.58 (0.36 to 0.79) | 0.58 (0.36 to 0.88) | 0.63 (0.38 to 0.97) | 0.25 (0.04 to 0.46) |
| Finland | 130 (78 to 202) | 159 (99 to 244) | 0.23 (0.09 to 0.39) | 2.6 (1.56 to 4.02) | 2.88 (1.78 to 4.41) | 0.39 (0.06 to 0.73) | 2.43 (1.39 to 3.92) | 2.39 (1.33 to 3.87) | 0.01 (-0.34 to 0.36) |
| France | 1233 (750 to 1908) | 1597 (997 to 2404) | 0.3 (0.13 to 0.49) | 2.13 (1.3 to 3.3) | 2.41 (1.51 to 3.63) | 0.34 (0.31 to 0.37) | 1.93 (1.13 to 3.06) | 1.89 (1.1 to 3) | -0.15 (-0.18 to -0.11) |
| Gabon | 6 (3 to 9) | 10 (6 to 15) | 0.75 (0.66 to 0.85) | 0.57 (0.35 to 0.87) | 0.57 (0.35 to 0.87) | -0.05 (-0.13 to 0.04) | 0.68 (0.42 to 1.02) | 0.66 (0.41 to 0.99) | -0.15 (-0.21 to -0.1) |
| Gambia | 7 (4 to 11) | 18 (11 to 27) | 1.48 (1.28 to 1.73) | 0.71 (0.44 to 1.1) | 0.78 (0.5 to 1.2) | 0.4 (0.31 to 0.48) | 1.02 (0.64 to 1.51) | 1.09 (0.7 to 1.59) | 0.28 (0.21 to 0.34) |
| Georgia | 95 (57 to 151) | 72 (43 to 112) | -0.25 (-0.34 to -0.13) | 1.73 (1.03 to 2.75) | 1.96 (1.18 to 3.07) | 0.67 (0.42 to 0.92) | 1.7 (1.01 to 2.7) | 1.83 (1.07 to 2.97) | 0.46 (0.2 to 0.71) |
| Germany | 1409 (850 to 2162) | 1719 (1061 to 2548) | 0.22 (0.06 to 0.42) | 1.76 (1.06 to 2.7) | 2.02 (1.25 to 3) | 0.38 (0.25 to 0.51) | 1.6 (0.92 to 2.57) | 1.64 (0.93 to 2.62) | -0.04 (-0.16 to 0.08) |
| Ghana | 120 (75 to 183) | 304 (195 to 461) | 1.53 (1.26 to 1.85) | 0.8 (0.5 to 1.22) | 0.97 (0.62 to 1.46) | 0.67 (0.61 to 0.74) | 1.13 (0.72 to 1.66) | 1.27 (0.81 to 1.86) | 0.44 (0.38 to 0.49) |
| Greece | 175 (106 to 273) | 175 (108 to 269) | 0 (-0.14 to 0.17) | 1.69 (1.02 to 2.62) | 1.69 (1.04 to 2.6) | -0.12 (-0.17 to -0.07) | 1.61 (0.94 to 2.55) | 1.5 (0.84 to 2.46) | -0.35 (-0.41 to -0.3) |
| Greenland | 2 (1 to 3) | 2 (1 to 3) | 0.03 (-0.1 to 0.19) | 3.51 (2.22 to 5.39) | 3.57 (2.33 to 5.24) | -0.04 (-0.16 to 0.08) | 4.3 (2.79 to 6.36) | 3.33 (2.19 to 4.89) | -1.03 (-1.09 to -0.97) |
| Grenada | 1 (0 to 1) | 1 (1 to 2) | 0.52 (0.38 to 0.69) | 0.79 (0.49 to 1.24) | 1 (0.62 to 1.54) | 0.82 (0.64 to 0.99) | 0.83 (0.51 to 1.29) | 0.99 (0.61 to 1.55) | 0.62 (0.45 to 0.79) |
| Guam | 1 (1 to 2) | 2 (1 to 3) | 0.58 (0.39 to 0.83) | 0.9 (0.55 to 1.38) | 1.14 (0.72 to 1.71) | 0.76 (0.66 to 0.85) | 1.05 (0.65 to 1.6) | 1.1 (0.69 to 1.65) | 0.05 (-0.04 to 0.14) |
| Guatemala | 84 (49 to 135) | 198 (115 to 321) | 1.34 (1.15 to 1.57) | 1.06 (0.61 to 1.7) | 1.11 (0.65 to 1.81) | 0.2 (0.11 to 0.28) | 1.15 (0.7 to 1.76) | 1.19 (0.71 to 1.87) | 0.14 (0.07 to 0.21) |
| Guinea | 52 (33 to 79) | 104 (66 to 158) | 0.99 (0.81 to 1.21) | 0.84 (0.53 to 1.28) | 0.82 (0.52 to 1.25) | -0.04 (-0.14 to 0.06) | 1.09 (0.7 to 1.57) | 1.17 (0.75 to 1.73) | 0.32 (0.23 to 0.41) |
| Guinea-Bissau | 9 (6 to 14) | 16 (10 to 25) | 0.82 (0.66 to 1.01) | 0.89 (0.55 to 1.39) | 0.86 (0.54 to 1.31) | -0.15 (-0.22 to -0.08) | 1.28 (0.82 to 1.89) | 1.23 (0.79 to 1.78) | -0.12 (-0.16 to -0.08) |
| Guyana | 7 (4 to 11) | 8 (5 to 13) | 0.2 (0.11 to 0.33) | 0.89 (0.54 to 1.38) | 1.06 (0.65 to 1.65) | 0.52 (0.45 to 0.58) | 0.99 (0.63 to 1.5) | 1.11 (0.69 to 1.69) | 0.24 (0.17 to 0.31) |
| Haiti | 49 (29 to 75) | 191 (104 to 344) | 2.9 (1.36 to 6.48) | 0.77 (0.46 to 1.19) | 1.54 (0.84 to 2.78) | 3.89 (1.6 to 6.23) | 0.86 (0.53 to 1.32) | 1.82 (0.97 to 3.39) | 4.13 (1.9 to 6.41) |
| Honduras | 43 (26 to 70) | 95 (57 to 150) | 1.2 (1 to 1.5) | 0.92 (0.55 to 1.48) | 0.97 (0.58 to 1.53) | -0.44 (-1.65 to 0.77) | 0.97 (0.6 to 1.52) | 1.04 (0.63 to 1.6) | -0.35 (-1.48 to 0.8) |
| Hungary | 335 (209 to 502) | 275 (173 to 424) | -0.18 (-0.27 to -0.06) | 3.22 (2.01 to 4.83) | 2.84 (1.79 to 4.38) | -0.72 (-0.83 to -0.61) | 2.9 (1.79 to 4.43) | 2.44 (1.45 to 3.93) | -0.87 (-0.98 to -0.75) |
| Iceland | 4 (2 to 7) | 6 (4 to 10) | 0.49 (0.35 to 0.68) | 1.63 (0.94 to 2.59) | 1.79 (1.06 to 2.76) | 0.19 (0.09 to 0.29) | 1.59 (0.92 to 2.53) | 1.63 (0.93 to 2.65) | -0.04 (-0.13 to 0.05) |
| India | 9472 (5655 to 14812) | 18004 (11161 to 26889) | 0.9 (0.76 to 1.04) | 1.11 (0.66 to 1.73) | 1.29 (0.8 to 1.93) | 0.48 (0.39 to 0.57) | 1.38 (0.86 to 2.08) | 1.41 (0.88 to 2.09) | 0.03 (-0.07 to 0.12) |
| Indonesia | 2173 (1381 to 3340) | 3120 (2032 to 4659) | 0.44 (0.35 to 0.55) | 1.17 (0.74 to 1.8) | 1.2 (0.78 to 1.8) | 0 (-0.17 to 0.17) | 1.46 (0.95 to 2.17) | 1.3 (0.86 to 1.91) | -0.51 (-0.66 to -0.36) |
| Iran (Islamic Republic of) | 899 (494 to 1453) | 812 (517 to 1232) | -0.1 (-0.33 to 0.13) | 1.54 (0.84 to 2.48) | 0.96 (0.61 to 1.46) | -0.54 (-0.91 to -0.17) | 1.78 (0.99 to 2.86) | 0.97 (0.62 to 1.46) | -1.07 (-1.43 to -0.72) |
| Iraq | 184 (107 to 299) | 430 (253 to 694) | 1.34 (1.18 to 1.51) | 1.04 (0.61 to 1.7) | 1.02 (0.6 to 1.65) | -0.12 (-0.18 to -0.06) | 1.18 (0.72 to 1.86) | 1.07 (0.65 to 1.68) | -0.42 (-0.46 to -0.38) |
| Ireland | 57 (33 to 89) | 84 (51 to 132) | 0.48 (0.34 to 0.66) | 1.58 (0.92 to 2.48) | 1.72 (1.03 to 2.69) | 0.16 (0.07 to 0.26) | 1.57 (0.91 to 2.49) | 1.63 (0.92 to 2.64) | 0.03 (-0.07 to 0.13) |
| Israel | 68 (40 to 109) | 138 (80 to 221) | 1.03 (0.84 to 1.23) | 1.37 (0.8 to 2.2) | 1.48 (0.86 to 2.37) | 0.26 (0.19 to 0.34) | 1.38 (0.81 to 2.19) | 1.44 (0.82 to 2.32) | 0.15 (0.07 to 0.22) |
| Italy | 1085 (665 to 1648) | 1072 (677 to 1593) | -0.01 (-0.08 to 0.06) | 1.91 (1.17 to 2.9) | 1.78 (1.12 to 2.64) | -1.06 (-1.39 to -0.73) | 1.71 (1.02 to 2.67) | 1.39 (0.81 to 2.24) | -1.5 (-1.83 to -1.15) |
| Jamaica | 19 (12 to 30) | 25 (15 to 39) | 0.3 (0.2 to 0.41) | 0.82 (0.49 to 1.27) | 0.89 (0.55 to 1.38) | 0.18 (0.1 to 0.27) | 0.84 (0.51 to 1.31) | 0.87 (0.54 to 1.37) | 0 (-0.09 to 0.09) |
| Japan | 2940 (1870 to 4484) | 3752 (2473 to 5418) | 0.28 (0.17 to 0.39) | 2.34 (1.49 to 3.56) | 2.94 (1.94 to 4.24) | 0.74 (0.66 to 0.82) | 2.14 (1.37 to 3.29) | 2.02 (1.25 to 3.09) | -0.27 (-0.38 to -0.17) |
| Jordan | 27 (16 to 44) | 88 (51 to 144) | 2.28 (2.09 to 2.47) | 0.71 (0.42 to 1.16) | 0.76 (0.44 to 1.24) | 0.05 (-0.05 to 0.15) | 0.8 (0.49 to 1.25) | 0.8 (0.48 to 1.26) | -0.16 (-0.25 to -0.06) |
| Kazakhstan | 263 (154 to 415) | 292 (175 to 455) | 0.11 (0 to 0.24) | 1.61 (0.94 to 2.53) | 1.59 (0.95 to 2.47) | 0.17 (0.05 to 0.28) | 1.65 (0.99 to 2.56) | 1.59 (0.95 to 2.5) | 0.1 (0 to 0.19) |
| Kenya | 137 (82 to 218) | 312 (190 to 484) | 1.28 (1.2 to 1.35) | 0.59 (0.36 to 0.94) | 0.62 (0.38 to 0.96) | 0.15 (0.02 to 0.27) | 0.75 (0.47 to 1.12) | 0.75 (0.47 to 1.13) | -0.02 (-0.11 to 0.06) |
| Kiribati | 0 (0 to 0) | 1 (0 to 1) | 0.82 (0.73 to 0.9) | 0.42 (0.25 to 0.66) | 0.48 (0.28 to 0.76) | 0.45 (0.26 to 0.65) | 0.49 (0.31 to 0.74) | 0.53 (0.32 to 0.81) | 0.26 (0.08 to 0.43) |
| Kuwait | 16 (9 to 26) | 44 (26 to 69) | 1.71 (1.56 to 1.89) | 0.92 (0.53 to 1.5) | 0.99 (0.58 to 1.57) | 0.19 (0.08 to 0.29) | 0.97 (0.59 to 1.52) | 0.99 (0.58 to 1.56) | -0.04 (-0.12 to 0.04) |
| Kyrgyzstan | 64 (37 to 102) | 77 (45 to 122) | 0.2 (0.1 to 0.32) | 1.43 (0.83 to 2.3) | 1.17 (0.69 to 1.87) | -0.84 (-0.99 to -0.7) | 1.51 (0.89 to 2.41) | 1.21 (0.72 to 1.89) | -0.89 (-1.04 to -0.73) |
| Lao People's Democratic Republic | 25 (15 to 38) | 43 (26 to 67) | 0.76 (0.64 to 0.89) | 0.59 (0.36 to 0.92) | 0.61 (0.37 to 0.94) | -0.06 (-0.17 to 0.05) | 0.71 (0.44 to 1.07) | 0.68 (0.42 to 1.03) | -0.32 (-0.41 to -0.23) |
| Latvia | 90 (56 to 136) | 47 (29 to 73) | -0.47 (-0.54 to -0.39) | 3.4 (2.1 to 5.13) | 2.48 (1.53 to 3.83) | -1.26 (-1.42 to -1.11) | 3.11 (1.88 to 4.78) | 2.08 (1.23 to 3.32) | -1.58 (-1.72 to -1.44) |
| Lebanon | 26 (16 to 42) | 46 (27 to 72) | 0.73 (0.58 to 0.88) | 0.8 (0.48 to 1.27) | 0.88 (0.52 to 1.39) | 0.26 (0.07 to 0.45) | 0.86 (0.52 to 1.33) | 0.88 (0.52 to 1.39) | 0 (-0.22 to 0.23) |
| Lesotho | 15 (9 to 23) | 20 (13 to 31) | 0.39 (0.27 to 0.53) | 0.81 (0.51 to 1.25) | 0.97 (0.62 to 1.47) | 0.75 (0.66 to 0.83) | 0.97 (0.61 to 1.47) | 1.12 (0.73 to 1.67) | 0.61 (0.55 to 0.68) |
| Liberia | 16 (10 to 24) | 35 (22 to 54) | 1.23 (1.03 to 1.43) | 0.8 (0.51 to 1.2) | 0.73 (0.46 to 1.13) | -0.35 (-0.54 to -0.16) | 1.01 (0.66 to 1.49) | 1 (0.63 to 1.48) | -0.02 (-0.11 to 0.08) |
| Libya | 33 (20 to 53) | 66 (40 to 102) | 0.98 (0.81 to 1.19) | 0.79 (0.47 to 1.26) | 0.98 (0.59 to 1.52) | 1.15 (0.71 to 1.59) | 0.92 (0.56 to 1.42) | 0.97 (0.58 to 1.5) | 0.54 (0.15 to 0.93) |
| Lithuania | 113 (69 to 170) | 78 (48 to 114) | -0.31 (-0.4 to -0.2) | 3.06 (1.89 to 4.64) | 2.79 (1.72 to 4.09) | -0.24 (-0.35 to -0.12) | 2.86 (1.74 to 4.36) | 2.25 (1.35 to 3.49) | -0.8 (-0.9 to -0.69) |
| Luxembourg | 8 (5 to 12) | 12 (7 to 19) | 0.55 (0.38 to 0.74) | 2.08 (1.26 to 3.25) | 1.98 (1.21 to 3.1) | -0.33 (-0.38 to -0.28) | 1.94 (1.14 to 3.09) | 1.77 (1.02 to 2.87) | -0.5 (-0.56 to -0.44) |
| Madagascar | 117 (73 to 179) | 253 (157 to 386) | 1.16 (0.97 to 1.36) | 0.98 (0.61 to 1.5) | 0.95 (0.59 to 1.45) | -0.18 (-0.34 to -0.03) | 1.28 (0.81 to 1.9) | 1.23 (0.78 to 1.82) | -0.21 (-0.3 to -0.11) |
| Malawi | 78 (48 to 123) | 158 (99 to 247) | 1.02 (0.88 to 1.18) | 0.82 (0.51 to 1.29) | 0.86 (0.54 to 1.34) | -0.01 (-0.15 to 0.12) | 1.12 (0.7 to 1.67) | 1.14 (0.74 to 1.71) | -0.04 (-0.14 to 0.06) |
| Malaysia | 111 (69 to 171) | 240 (146 to 369) | 1.16 (1 to 1.35) | 0.63 (0.39 to 0.97) | 0.77 (0.47 to 1.18) | 0.54 (0.38 to 0.69) | 0.74 (0.47 to 1.13) | 0.8 (0.49 to 1.23) | 0.07 (-0.08 to 0.23) |
| Maldives | 1 (1 to 2) | 3 (2 to 5) | 1.72 (1.46 to 2.06) | 0.54 (0.32 to 0.87) | 0.65 (0.4 to 1.01) | 0.62 (-0.02 to 1.26) | 0.67 (0.41 to 1.01) | 0.7 (0.43 to 1.07) | 0.13 (-0.43 to 0.7) |
| Mali | 69 (44 to 107) | 165 (105 to 257) | 1.38 (1.16 to 1.62) | 0.8 (0.5 to 1.24) | 0.75 (0.48 to 1.17) | -0.31 (-0.4 to -0.23) | 1.09 (0.7 to 1.59) | 1.11 (0.72 to 1.64) | -0.01 (-0.08 to 0.05) |
| Malta | 6 (4 to 10) | 9 (5 to 13) | 0.44 (0.27 to 0.68) | 1.65 (0.98 to 2.67) | 2.01 (1.22 to 3.07) | 0.91 (0.84 to 0.99) | 1.66 (0.96 to 2.71) | 1.8 (1.02 to 2.93) | 0.61 (0.51 to 0.7) |
| Marshall Islands | 0 (0 to 0) | 0 (0 to 1) | 0.57 (0.48 to 0.67) | 0.48 (0.28 to 0.77) | 0.6 (0.36 to 0.95) | 0.82 (0.74 to 0.91) | 0.62 (0.39 to 0.95) | 0.67 (0.42 to 1.02) | 0.27 (0.19 to 0.34) |
| Mauritania | 20 (12 to 30) | 36 (23 to 55) | 0.85 (0.65 to 1.06) | 0.95 (0.59 to 1.45) | 0.91 (0.57 to 1.36) | -0.16 (-0.36 to 0.03) | 1.29 (0.82 to 1.89) | 1.19 (0.76 to 1.73) | -0.26 (-0.44 to -0.07) |
| Mauritius | 10 (6 to 15) | 17 (11 to 25) | 0.74 (0.47 to 1.09) | 0.88 (0.55 to 1.34) | 1.32 (0.83 to 1.95) | 1.44 (1.38 to 1.49) | 0.97 (0.61 to 1.46) | 1.17 (0.73 to 1.74) | 0.64 (0.59 to 0.7) |
| Mexico | 1494 (923 to 2343) | 2099 (1342 to 3166) | 0.4 (0.31 to 0.51) | 1.75 (1.08 to 2.74) | 1.68 (1.07 to 2.53) | 1.56 (1.07 to 2.06) | 2.14 (1.38 to 3.22) | 1.72 (1.1 to 2.58) | 0.74 (0.31 to 1.18) |
| Micronesia (Federated States of) | 1 (0 to 1) | 1 (0 to 1) | 0.28 (0.19 to 0.38) | 0.51 (0.3 to 0.8) | 0.66 (0.4 to 1.03) | 0.97 (0.44 to 1.49) | 0.64 (0.39 to 0.96) | 0.73 (0.45 to 1.12) | 0.5 (0.05 to 0.95) |
| Monaco | 1 (1 to 1) | 1 (1 to 2) | 0.47 (0.26 to 0.71) | 3.07 (2.02 to 4.53) | 3.64 (2.38 to 5.24) | 0.62 (0.58 to 0.66) | 2.22 (1.4 to 3.42) | 2.52 (1.55 to 3.89) | 0.48 (0.42 to 0.54) |
| Mongolia | 27 (16 to 43) | 53 (31 to 84) | 0.95 (0.78 to 1.17) | 1.26 (0.73 to 2.01) | 1.56 (0.92 to 2.48) | 1.04 (0.91 to 1.16) | 1.41 (0.84 to 2.21) | 1.61 (0.96 to 2.51) | 0.69 (0.61 to 0.77) |
| Montenegro | 15 (9 to 24) | 16 (10 to 25) | 0.07 (-0.05 to 0.21) | 2.39 (1.44 to 3.78) | 2.57 (1.56 to 4.02) | 0.23 (0.16 to 0.3) | 2.37 (1.43 to 3.76) | 2.4 (1.41 to 3.81) | 0 (-0.08 to 0.07) |
| Morocco | 228 (135 to 369) | 361 (216 to 568) | 0.58 (0.47 to 0.71) | 0.9 (0.53 to 1.46) | 1 (0.6 to 1.58) | 0.22 (0.13 to 0.31) | 0.98 (0.59 to 1.53) | 1.02 (0.61 to 1.61) | 0.01 (-0.08 to 0.09) |
| Mozambique | 133 (83 to 205) | 326 (199 to 502) | 1.45 (1.22 to 1.72) | 1.02 (0.64 to 1.57) | 1.1 (0.68 to 1.7) | 0.17 (0.01 to 0.33) | 1.37 (0.86 to 1.99) | 1.57 (1.02 to 2.3) | 0.45 (0.32 to 0.58) |
| Myanmar | 319 (190 to 507) | 521 (327 to 765) | 0.63 (0.37 to 1.16) | 0.78 (0.46 to 1.23) | 0.95 (0.6 to 1.4) | 1.33 (0.11 to 2.56) | 0.87 (0.53 to 1.34) | 1 (0.64 to 1.48) | 1.15 (-0.01 to 2.33) |
| Namibia | 12 (7 to 18) | 22 (14 to 33) | 0.86 (0.7 to 1.07) | 0.84 (0.53 to 1.3) | 0.92 (0.58 to 1.38) | 0.22 (0.15 to 0.3) | 1.06 (0.68 to 1.56) | 1.08 (0.68 to 1.59) | -0.03 (-0.13 to 0.08) |
| Nauru | 0 (0 to 0) | 0 (0 to 0) | 0.22 (0.09 to 0.36) | 0.76 (0.47 to 1.16) | 0.9 (0.55 to 1.39) | 0.51 (0.44 to 0.58) | 1.03 (0.65 to 1.53) | 1.17 (0.73 to 1.74) | 0.39 (0.34 to 0.45) |
| Nepal | 178 (103 to 287) | 335 (200 to 525) | 0.88 (0.74 to 1.1) | 0.91 (0.53 to 1.47) | 1.1 (0.66 to 1.73) | 0.68 (0.26 to 1.11) | 1.11 (0.68 to 1.72) | 1.2 (0.73 to 1.86) | 0.27 (-0.1 to 0.64) |
| Netherlands | 205 (125 to 318) | 321 (205 to 485) | 0.57 (0.38 to 0.81) | 1.37 (0.84 to 2.13) | 1.87 (1.2 to 2.83) | 0.9 (0.53 to 1.28) | 1.29 (0.77 to 2.05) | 1.46 (0.86 to 2.3) | 0.23 (-0.04 to 0.5) |
| New Zealand | 105 (64 to 167) | 152 (94 to 235) | 0.45 (0.32 to 0.62) | 3.07 (1.86 to 4.88) | 3.39 (2.1 to 5.23) | 0.31 (0.26 to 0.37) | 3.04 (1.82 to 4.83) | 3.15 (1.86 to 5.07) | 0.09 (0.02 to 0.17) |
| Nicaragua | 34 (19 to 55) | 56 (33 to 89) | 0.68 (0.57 to 0.82) | 0.86 (0.49 to 1.41) | 0.86 (0.51 to 1.36) | -0.23 (-0.67 to 0.22) | 0.91 (0.54 to 1.43) | 0.91 (0.55 to 1.41) | -0.22 (-0.73 to 0.29) |
| Niger | 61 (38 to 95) | 166 (105 to 260) | 1.72 (1.53 to 1.92) | 0.76 (0.47 to 1.18) | 0.71 (0.45 to 1.11) | -0.32 (-0.42 to -0.21) | 1.12 (0.73 to 1.68) | 1.11 (0.72 to 1.65) | -0.04 (-0.13 to 0.05) |
| Nigeria | 733 (469 to 1125) | 1656 (1059 to 2564) | 1.26 (1.2 to 1.33) | 0.81 (0.52 to 1.25) | 0.77 (0.49 to 1.19) | -0.16 (-0.29 to -0.02) | 1.07 (0.71 to 1.59) | 1.07 (0.71 to 1.58) | 0.06 (-0.05 to 0.16) |
| Niue | 0 (0 to 0) | 0 (0 to 0) | -0.03 (-0.16 to 0.14) | 0.93 (0.59 to 1.4) | 1.26 (0.81 to 1.88) | 1.12 (0.72 to 1.51) | 0.98 (0.62 to 1.47) | 1.15 (0.73 to 1.72) | 0.58 (0.17 to 0.98) |
| North Macedonia | 43 (26 to 69) | 48 (30 to 75) | 0.13 (-0.03 to 0.31) | 2.12 (1.3 to 3.41) | 2.25 (1.37 to 3.49) | 0.18 (0.1 to 0.26) | 2.12 (1.3 to 3.41) | 2.16 (1.29 to 3.45) | 0.03 (-0.06 to 0.12) |
| Northern Mariana Islands | 0 (0 to 1) | 1 (0 to 1) | 0.26 (0.05 to 0.53) | 1.09 (0.67 to 1.68) | 1.46 (0.91 to 2.16) | 0.9 (0.7 to 1.1) | 1.36 (0.86 to 2.02) | 1.41 (0.89 to 2.09) | -0.03 (-0.13 to 0.07) |
| Norway | 154 (100 to 225) | 207 (138 to 299) | 0.34 (0.28 to 0.42) | 3.63 (2.35 to 5.3) | 3.88 (2.57 to 5.59) | 0.1 (0.05 to 0.15) | 2.88 (1.86 to 4.3) | 2.82 (1.8 to 4.22) | -0.18 (-0.25 to -0.12) |
| Oman | 19 (11 to 29) | 49 (29 to 79) | 1.62 (1.41 to 1.86) | 0.95 (0.57 to 1.49) | 1.06 (0.63 to 1.72) | 0.37 (0.31 to 0.44) | 1.16 (0.71 to 1.78) | 1.14 (0.69 to 1.78) | -0.15 (-0.23 to -0.08) |
| Pakistan | 623 (383 to 946) | 1576 (974 to 2376) | 1.53 (1.38 to 1.69) | 0.55 (0.34 to 0.84) | 0.7 (0.43 to 1.06) | 0.9 (0.62 to 1.19) | 0.69 (0.43 to 1.02) | 0.84 (0.54 to 1.24) | 0.81 (0.58 to 1.05) |
| Palau | 0 (0 to 0) | 0 (0 to 1) | 0.62 (0.39 to 0.96) | 1.38 (0.85 to 2.15) | 1.91 (1.22 to 2.82) | 0.98 (0.94 to 1.03) | 1.63 (1.03 to 2.48) | 1.81 (1.16 to 2.68) | 0.25 (0.21 to 0.28) |
| Palestine | 18 (11 to 30) | 44 (26 to 70) | 1.41 (1.01 to 1.68) | 0.87 (0.51 to 1.43) | 0.88 (0.52 to 1.4) | -0.03 (-0.27 to 0.21) | 0.99 (0.61 to 1.61) | 0.96 (0.59 to 1.49) | -0.21 (-0.41 to 0) |
| Panama | 23 (13 to 36) | 40 (24 to 62) | 0.77 (0.66 to 0.91) | 0.95 (0.56 to 1.5) | 0.96 (0.58 to 1.5) | -0.01 (-0.1 to 0.08) | 1 (0.6 to 1.56) | 0.96 (0.58 to 1.51) | -0.19 (-0.28 to -0.1) |
| Papua New Guinea | 21 (12 to 35) | 67 (39 to 107) | 2.11 (1.92 to 2.36) | 0.53 (0.3 to 0.84) | 0.68 (0.4 to 1.09) | 0.44 (-0.29 to 1.17) | 0.66 (0.41 to 1) | 0.84 (0.51 to 1.28) | 0.45 (-0.18 to 1.09) |
| Paraguay | 40 (23 to 65) | 71 (42 to 112) | 0.77 (0.65 to 0.93) | 0.99 (0.57 to 1.61) | 1.02 (0.61 to 1.62) | 0.11 (0.01 to 0.21) | 1.06 (0.63 to 1.66) | 1.04 (0.62 to 1.64) | -0.07 (-0.17 to 0.03) |
| Peru | 176 (108 to 274) | 297 (182 to 458) | 0.68 (0.54 to 0.83) | 0.81 (0.5 to 1.26) | 0.87 (0.54 to 1.35) | 0.29 (0.22 to 0.36) | 0.87 (0.55 to 1.34) | 0.88 (0.54 to 1.35) | 0.02 (-0.04 to 0.08) |
| Philippines | 422 (251 to 653) | 758 (460 to 1140) | 0.8 (0.6 to 0.94) | 0.67 (0.4 to 1.03) | 0.68 (0.41 to 1.02) | 0.16 (-0.18 to 0.49) | 0.78 (0.48 to 1.16) | 0.74 (0.46 to 1.11) | -0.06 (-0.35 to 0.23) |
| Poland | 1017 (624 to 1597) | 1070 (676 to 1619) | 0.05 (-0.01 to 0.13) | 2.66 (1.64 to 4.19) | 2.78 (1.76 to 4.21) | 0.15 (0.07 to 0.22) | 2.59 (1.58 to 4.09) | 2.42 (1.46 to 3.8) | -0.28 (-0.38 to -0.18) |
| Portugal | 156 (96 to 239) | 147 (90 to 224) | -0.06 (-0.19 to 0.1) | 1.54 (0.94 to 2.36) | 1.38 (0.85 to 2.1) | -0.74 (-0.84 to -0.63) | 1.46 (0.88 to 2.28) | 1.1 (0.65 to 1.75) | -1.3 (-1.41 to -1.19) |
| Puerto Rico | 54 (35 to 81) | 76 (49 to 111) | 0.39 (0.17 to 0.68) | 1.51 (0.96 to 2.23) | 2.15 (1.39 to 3.14) | 1.4 (1.18 to 1.63) | 1.52 (0.96 to 2.26) | 1.64 (1.04 to 2.48) | 0.49 (0.22 to 0.76) |
| Qatar | 6 (3 to 9) | 38 (22 to 62) | 5.56 (4.98 to 6.16) | 1.3 (0.76 to 2.13) | 1.32 (0.77 to 2.17) | 0.35 (0.17 to 0.52) | 1.32 (0.78 to 2.05) | 1.27 (0.76 to 2.02) | -0.05 (-0.16 to 0.05) |
| Republic of Korea | 789 (463 to 1271) | 1035 (631 to 1598) | 0.31 (0.16 to 0.52) | 1.78 (1.05 to 2.87) | 1.94 (1.18 to 2.99) | 0.04 (-0.05 to 0.14) | 1.85 (1.11 to 2.95) | 1.7 (1.03 to 2.7) | -0.54 (-0.62 to -0.46) |
| Republic of Moldova | 100 (62 to 155) | 72 (45 to 112) | -0.28 (-0.37 to -0.17) | 2.25 (1.39 to 3.48) | 1.95 (1.22 to 3.04) | -0.54 (-0.63 to -0.45) | 2.25 (1.39 to 3.45) | 1.77 (1.07 to 2.83) | -0.9 (-1.02 to -0.78) |
| Romania | 667 (403 to 1054) | 499 (304 to 764) | -0.25 (-0.34 to -0.13) | 2.85 (1.72 to 4.5) | 2.6 (1.58 to 3.97) | -0.5 (-0.62 to -0.37) | 2.76 (1.64 to 4.35) | 2.33 (1.36 to 3.72) | -0.75 (-0.88 to -0.62) |
| Russian Federation | 4328 (2668 to 6617) | 3794 (2373 to 5721) | -0.12 (-0.17 to -0.07) | 2.87 (1.77 to 4.38) | 2.59 (1.62 to 3.9) | -0.28 (-0.58 to 0.02) | 2.73 (1.68 to 4.21) | 2.28 (1.39 to 3.55) | -0.59 (-0.84 to -0.35) |
| Rwanda | 74 (46 to 112) | 125 (78 to 193) | 0.68 (0.54 to 0.85) | 1.04 (0.64 to 1.56) | 0.98 (0.61 to 1.52) | -0.32 (-0.51 to -0.12) | 1.47 (0.94 to 2.15) | 1.28 (0.81 to 1.87) | -0.62 (-0.76 to -0.49) |
| Saint Kitts and Nevis | 1 (0 to 1) | 1 (1 to 1) | 0.67 (0.47 to 0.95) | 1.31 (0.81 to 2.01) | 1.52 (0.94 to 2.27) | 0.35 (0.24 to 0.47) | 1.41 (0.88 to 2.16) | 1.49 (0.93 to 2.25) | 0.08 (-0.02 to 0.18) |
| Saint Lucia | 1 (1 to 2) | 2 (1 to 2) | 0.61 (0.48 to 0.79) | 0.73 (0.45 to 1.13) | 0.93 (0.58 to 1.4) | 0.85 (0.75 to 0.95) | 0.79 (0.49 to 1.19) | 0.89 (0.55 to 1.38) | 0.42 (0.32 to 0.52) |
| Saint Vincent and the Grenadines | 1 (0 to 1) | 1 (1 to 2) | 0.31 (0.18 to 0.47) | 0.75 (0.45 to 1.17) | 0.95 (0.59 to 1.46) | 0.85 (0.71 to 0.99) | 0.8 (0.49 to 1.22) | 0.92 (0.57 to 1.42) | 0.49 (0.35 to 0.62) |
| Samoa | 1 (1 to 2) | 2 (1 to 2) | 0.58 (0.35 to 0.87) | 0.59 (0.35 to 0.93) | 0.72 (0.43 to 1.09) | 1.29 (0.23 to 2.37) | 0.68 (0.42 to 1.04) | 0.8 (0.49 to 1.2) | 1.12 (0.14 to 2.12) |
| San Marino | 1 (0 to 1) | 1 (1 to 2) | 0.8 (0.58 to 1.07) | 2.55 (1.64 to 3.88) | 3.27 (2.04 to 4.83) | 0.88 (0.83 to 0.94) | 2.33 (1.46 to 3.59) | 2.54 (1.5 to 3.95) | 0.32 (0.25 to 0.39) |
| Sao Tome and Principe | 1 (1 to 2) | 2 (1 to 3) | 0.91 (0.68 to 1.14) | 0.93 (0.59 to 1.44) | 1.05 (0.66 to 1.63) | 0.36 (0.28 to 0.45) | 1.19 (0.77 to 1.74) | 1.37 (0.85 to 2.07) | 0.49 (0.44 to 0.54) |
| Saudi Arabia | 252 (143 to 426) | 808 (452 to 1367) | 2.21 (1.79 to 2.68) | 1.57 (0.89 to 2.65) | 2.26 (1.26 to 3.83) | 1.41 (1.29 to 1.54) | 1.8 (1.07 to 2.83) | 2.21 (1.28 to 3.6) | 0.82 (0.69 to 0.95) |
| Senegal | 57 (36 to 89) | 121 (76 to 187) | 1.12 (0.95 to 1.3) | 0.75 (0.47 to 1.17) | 0.8 (0.5 to 1.24) | 0.24 (0.16 to 0.33) | 1.05 (0.69 to 1.55) | 1.08 (0.68 to 1.56) | 0.12 (0.06 to 0.18) |
| Serbia | 205 (127 to 319) | 209 (130 to 321) | 0.02 (-0.12 to 0.18) | 2.19 (1.35 to 3.39) | 2.39 (1.49 to 3.67) | 0.36 (0.26 to 0.46) | 2.12 (1.31 to 3.36) | 2.22 (1.33 to 3.52) | 0.16 (0.05 to 0.28) |
| Seychelles | 1 (0 to 1) | 1 (1 to 2) | 0.67 (0.47 to 0.92) | 1.01 (0.65 to 1.52) | 1.21 (0.78 to 1.83) | 0.49 (0.36 to 0.62) | 1.12 (0.72 to 1.65) | 1.15 (0.73 to 1.72) | -0.03 (-0.15 to 0.09) |
| Sierra Leone | 30 (19 to 46) | 66 (42 to 100) | 1.2 (0.99 to 1.43) | 0.82 (0.52 to 1.27) | 0.8 (0.51 to 1.21) | -0.06 (-0.38 to 0.26) | 1.06 (0.68 to 1.55) | 1.12 (0.73 to 1.66) | 0.36 (0.12 to 0.59) |
| Singapore | 46 (27 to 74) | 85 (52 to 132) | 0.84 (0.66 to 1.11) | 1.51 (0.89 to 2.42) | 1.49 (0.92 to 2.34) | -0.2 (-0.28 to -0.12) | 1.51 (0.89 to 2.38) | 1.42 (0.83 to 2.28) | -0.35 (-0.43 to -0.27) |
| Slovakia | 152 (93 to 239) | 164 (100 to 253) | 0.08 (-0.04 to 0.22) | 2.88 (1.76 to 4.53) | 3.02 (1.83 to 4.65) | 0.12 (0.01 to 0.22) | 2.8 (1.69 to 4.4) | 2.68 (1.55 to 4.26) | -0.2 (-0.31 to -0.09) |
| Slovenia | 66 (41 to 102) | 73 (46 to 110) | 0.11 (-0.02 to 0.29) | 3.34 (2.06 to 5.15) | 3.54 (2.22 to 5.32) | 0.52 (0.39 to 0.66) | 3.14 (1.93 to 4.93) | 2.9 (1.7 to 4.61) | 0.11 (-0.05 to 0.27) |
| Solomon Islands | 2 (1 to 4) | 5 (3 to 9) | 1.33 (1.17 to 1.5) | 0.67 (0.38 to 1.07) | 0.81 (0.46 to 1.32) | 0.68 (0.47 to 0.89) | 0.88 (0.54 to 1.33) | 1 (0.59 to 1.57) | 0.45 (0.28 to 0.63) |
| Somalia | 62 (38 to 95) | 180 (112 to 279) | 1.92 (1.68 to 2.17) | 0.86 (0.53 to 1.33) | 0.88 (0.55 to 1.37) | 0.19 (-0.03 to 0.41) | 1.21 (0.76 to 1.82) | 1.25 (0.8 to 1.85) | 0.18 (0.02 to 0.35) |
| South Africa | 388 (248 to 589) | 536 (343 to 801) | 0.38 (0.29 to 0.48) | 1.05 (0.67 to 1.6) | 0.96 (0.62 to 1.44) | -0.14 (-0.3 to 0.02) | 1.25 (0.81 to 1.84) | 1.02 (0.66 to 1.52) | -0.52 (-0.69 to -0.34) |
| South Sudan | 57 (35 to 87) | 88 (56 to 136) | 0.55 (0.43 to 0.7) | 0.97 (0.6 to 1.48) | 0.95 (0.6 to 1.47) | 0.17 (-0.11 to 0.46) | 1.32 (0.86 to 1.93) | 1.27 (0.83 to 1.87) | 0.07 (-0.12 to 0.26) |
| Spain | 611 (369 to 964) | 812 (498 to 1242) | 0.33 (0.16 to 0.55) | 1.57 (0.95 to 2.49) | 1.76 (1.08 to 2.7) | 0.16 (0.08 to 0.24) | 1.49 (0.87 to 2.39) | 1.53 (0.89 to 2.49) | -0.07 (-0.13 to 0) |
| Sri Lanka | 137 (85 to 212) | 221 (138 to 330) | 0.61 (0.38 to 0.99) | 0.8 (0.49 to 1.23) | 1.01 (0.63 to 1.51) | 0.73 (-0.17 to 1.63) | 0.89 (0.56 to 1.33) | 0.97 (0.61 to 1.45) | 0.27 (-0.61 to 1.15) |
| Sudan | 208 (128 to 325) | 434 (267 to 665) | 1.09 (0.88 to 1.33) | 1.03 (0.63 to 1.61) | 1.06 (0.65 to 1.63) | 0.02 (-0.1 to 0.15) | 1.26 (0.8 to 1.96) | 1.27 (0.79 to 1.93) | -0.05 (-0.14 to 0.04) |
| Suriname | 3 (2 to 5) | 5 (3 to 8) | 0.73 (0.52 to 0.93) | 0.78 (0.48 to 1.22) | 0.9 (0.55 to 1.35) | 0.54 (0.48 to 0.59) | 0.82 (0.51 to 1.27) | 0.89 (0.55 to 1.34) | 0.3 (0.27 to 0.34) |
| Sweden | 303 (198 to 449) | 408 (268 to 584) | 0.34 (0.2 to 0.5) | 3.53 (2.3 to 5.23) | 3.99 (2.62 to 5.71) | 0.4 (0.35 to 0.44) | 2.75 (1.73 to 4.11) | 2.78 (1.73 to 4.21) | -0.02 (-0.11 to 0.07) |
| Switzerland | 184 (111 to 286) | 218 (133 to 335) | 0.18 (0.03 to 0.36) | 2.68 (1.62 to 4.16) | 2.48 (1.51 to 3.82) | -0.67 (-0.82 to -0.52) | 2.38 (1.37 to 3.82) | 2.05 (1.16 to 3.32) | -0.93 (-1.12 to -0.73) |
| Syrian Arab Republic | 90 (51 to 147) | 134 (77 to 216) | 0.49 (0.38 to 0.63) | 0.7 (0.4 to 1.14) | 0.92 (0.53 to 1.49) | 0.96 (0.73 to 1.19) | 0.77 (0.46 to 1.22) | 0.95 (0.56 to 1.51) | 0.69 (0.45 to 0.94) |
| Taiwan (Province of China) | 146 (86 to 231) | 165 (105 to 243) | 0.13 (-0.04 to 0.35) | 0.71 (0.42 to 1.13) | 0.7 (0.44 to 1.03) | -0.45 (-0.68 to -0.21) | 0.77 (0.46 to 1.19) | 0.54 (0.34 to 0.82) | -1.57 (-1.82 to -1.33) |
| Tajikistan | 69 (39 to 111) | 104 (61 to 169) | 0.52 (0.41 to 0.65) | 1.28 (0.72 to 2.07) | 1.1 (0.64 to 1.78) | -0.72 (-0.95 to -0.49) | 1.4 (0.83 to 2.22) | 1.16 (0.7 to 1.84) | -0.85 (-1.08 to -0.61) |
| Thailand | 455 (280 to 703) | 651 (407 to 979) | 0.43 (0.26 to 0.66) | 0.8 (0.49 to 1.24) | 0.93 (0.58 to 1.4) | 0.31 (0.19 to 0.44) | 0.88 (0.55 to 1.32) | 0.81 (0.5 to 1.24) | -0.48 (-0.61 to -0.34) |
| Timor-Leste | 4 (2 to 6) | 8 (4 to 12) | 0.9 (0.76 to 1.03) | 0.5 (0.31 to 0.8) | 0.56 (0.34 to 0.88) | 0.35 (0.21 to 0.5) | 0.61 (0.38 to 0.93) | 0.65 (0.4 to 0.98) | 0.13 (0.01 to 0.26) |
| Togo | 29 (18 to 45) | 69 (44 to 104) | 1.37 (1.17 to 1.63) | 0.79 (0.5 to 1.23) | 0.87 (0.55 to 1.32) | 0.34 (0.24 to 0.45) | 1.15 (0.74 to 1.68) | 1.16 (0.74 to 1.69) | 0.06 (-0.01 to 0.14) |
| Tokelau | 0 (0 to 0) | 0 (0 to 0) | 0.04 (-0.09 to 0.19) | 0.79 (0.49 to 1.18) | 0.98 (0.62 to 1.5) | 0.82 (0.72 to 0.93) | 0.91 (0.57 to 1.36) | 1.04 (0.66 to 1.57) | 0.45 (0.35 to 0.55) |
| Tonga | 0 (0 to 1) | 1 (0 to 1) | 0.21 (0.13 to 0.3) | 0.5 (0.3 to 0.8) | 0.58 (0.34 to 0.92) | 0.53 (0.25 to 0.8) | 0.58 (0.36 to 0.89) | 0.62 (0.38 to 0.98) | 0.24 (-0.02 to 0.5) |
| Trinidad and Tobago | 9 (5 to 14) | 12 (8 to 18) | 0.38 (0.26 to 0.53) | 0.73 (0.45 to 1.12) | 0.87 (0.54 to 1.32) | 0.74 (0.67 to 0.81) | 0.77 (0.48 to 1.17) | 0.83 (0.52 to 1.28) | 0.36 (0.3 to 0.42) |
| Tunisia | 68 (41 to 108) | 108 (65 to 167) | 0.57 (0.44 to 0.73) | 0.81 (0.48 to 1.28) | 0.93 (0.56 to 1.44) | 0.51 (0.44 to 0.58) | 0.88 (0.54 to 1.36) | 0.91 (0.55 to 1.42) | 0.13 (0.06 to 0.2) |
| Turkey | 439 (265 to 701) | 791 (483 to 1236) | 0.8 (0.63 to 1.02) | 0.73 (0.44 to 1.17) | 0.97 (0.59 to 1.52) | 0.92 (0.35 to 1.49) | 0.79 (0.48 to 1.23) | 0.94 (0.57 to 1.48) | 0.57 (0.03 to 1.1) |
| Turkmenistan | 41 (24 to 66) | 61 (36 to 96) | 0.48 (0.38 to 0.62) | 1.11 (0.65 to 1.78) | 1.2 (0.71 to 1.89) | 0.33 (0.24 to 0.43) | 1.19 (0.72 to 1.87) | 1.22 (0.72 to 1.92) | 0.16 (0.03 to 0.28) |
| Tuvalu | 0 (0 to 0) | 0 (0 to 0) | 0.54 (0.36 to 0.74) | 0.79 (0.49 to 1.2) | 0.96 (0.59 to 1.45) | 0.6 (0.51 to 0.7) | 0.9 (0.57 to 1.36) | 1.03 (0.64 to 1.55) | 0.4 (0.3 to 0.49) |
| Uganda | 145 (90 to 224) | 356 (221 to 559) | 1.45 (1.28 to 1.65) | 0.84 (0.52 to 1.3) | 0.87 (0.54 to 1.36) | 0.09 (-0.06 to 0.23) | 1.16 (0.74 to 1.72) | 1.19 (0.75 to 1.75) | 0.05 (-0.03 to 0.13) |
| Ukraine | 1558 (944 to 2442) | 1153 (711 to 1758) | -0.26 (-0.36 to -0.13) | 2.96 (1.79 to 4.64) | 2.62 (1.62 to 3.99) | -0.75 (-0.92 to -0.59) | 2.77 (1.65 to 4.37) | 2.32 (1.36 to 3.67) | -0.96 (-1.16 to -0.77) |
| United Arab Emirates | 18 (11 to 29) | 101 (61 to 159) | 4.48 (4.08 to 4.92) | 0.98 (0.58 to 1.57) | 1.09 (0.66 to 1.72) | 0.23 (0.14 to 0.32) | 1.09 (0.66 to 1.69) | 1.05 (0.63 to 1.63) | -0.24 (-0.32 to -0.16) |
| United Kingdom | 1027 (637 to 1566) | 1347 (845 to 2040) | 0.31 (0.26 to 0.37) | 1.79 (1.11 to 2.73) | 2 (1.26 to 3.03) | 0.39 (0.32 to 0.46) | 1.65 (1 to 2.62) | 1.69 (1.01 to 2.68) | 0.06 (-0.02 to 0.14) |
| United Republic of Tanzania | 231 (141 to 359) | 516 (320 to 806) | 1.24 (1.07 to 1.4) | 0.89 (0.54 to 1.39) | 0.91 (0.56 to 1.42) | 0.08 (-0.05 to 0.2) | 1.19 (0.76 to 1.75) | 1.19 (0.77 to 1.8) | 0 (-0.1 to 0.09) |
| United States of America | 7645 (4962 to 11361) | 11628 (7726 to 16678) | 0.52 (0.42 to 0.62) | 3.01 (1.96 to 4.48) | 3.55 (2.36 to 5.09) | 0.29 (0 to 0.59) | 2.73 (1.76 to 4.12) | 2.65 (1.75 to 3.96) | -0.47 (-0.77 to -0.16) |
| United States Virgin Islands | 1 (1 to 2) | 2 (1 to 2) | 0.28 (0.08 to 0.54) | 1.24 (0.77 to 1.9) | 1.62 (1.03 to 2.38) | 0.84 (0.74 to 0.93) | 1.32 (0.83 to 2.01) | 1.38 (0.86 to 2.06) | 0.04 (-0.06 to 0.15) |
| Uruguay | 58 (35 to 92) | 61 (37 to 96) | 0.06 (-0.06 to 0.21) | 1.85 (1.11 to 2.93) | 1.79 (1.08 to 2.8) | -0.28 (-0.37 to -0.19) | 1.81 (1.07 to 2.89) | 1.65 (0.98 to 2.64) | -0.47 (-0.56 to -0.37) |
| Uzbekistan | 260 (148 to 423) | 415 (242 to 664) | 0.6 (0.47 to 0.74) | 1.24 (0.71 to 2.02) | 1.23 (0.72 to 1.97) | -0.16 (-0.24 to -0.08) | 1.31 (0.79 to 2.09) | 1.27 (0.76 to 2.02) | -0.23 (-0.29 to -0.16) |
| Vanuatu | 1 (0 to 1) | 2 (1 to 3) | 1.34 (1.19 to 1.53) | 0.5 (0.3 to 0.79) | 0.6 (0.36 to 0.92) | 0.14 (-0.82 to 1.11) | 0.62 (0.39 to 0.93) | 0.71 (0.44 to 1.04) | -0.03 (-0.88 to 0.84) |
| Venezuela (Bolivarian Republic of) | 210 (124 to 338) | 324 (195 to 508) | 0.54 (0.4 to 0.76) | 1.11 (0.66 to 1.8) | 1.15 (0.7 to 1.81) | -0.11 (-0.86 to 0.66) | 1.18 (0.72 to 1.85) | 1.16 (0.7 to 1.84) | -0.27 (-0.98 to 0.45) |
| Viet Nam | 438 (265 to 691) | 846 (536 to 1277) | 0.93 (0.74 to 1.15) | 0.64 (0.39 to 1.02) | 0.88 (0.56 to 1.32) | 1.04 (0.91 to 1.17) | 0.76 (0.48 to 1.16) | 0.88 (0.56 to 1.34) | 0.45 (0.34 to 0.56) |
| Yemen | 98 (58 to 159) | 229 (132 to 367) | 1.33 (1.18 to 1.48) | 0.71 (0.42 to 1.16) | 0.73 (0.42 to 1.17) | 0.06 (-0.03 to 0.15) | 0.87 (0.53 to 1.34) | 0.83 (0.5 to 1.28) | -0.13 (-0.19 to -0.07) |
| Zambia | 64 (40 to 98) | 155 (96 to 239) | 1.43 (1.26 to 1.6) | 0.8 (0.5 to 1.24) | 0.85 (0.53 to 1.31) | 0.11 (-0.02 to 0.24) | 1.15 (0.74 to 1.71) | 1.18 (0.76 to 1.75) | 0.02 (-0.06 to 0.1) |
| Zimbabwe | 75 (47 to 115) | 124 (77 to 193) | 0.66 (0.47 to 0.94) | 0.73 (0.45 to 1.11) | 0.83 (0.51 to 1.29) | 0.11 (-0.05 to 0.28) | 0.99 (0.64 to 1.44) | 1.05 (0.67 to 1.58) | -0.06 (-0.21 to 0.08) |

YLDs, years lived with disability; CR, crude rate; ASR, age-standardized rate; EAPC, estimated annual percentage change; UI, uncertainty interval; CI, confidence interval.
